# Recent Advancement in Drug Design and Discovery of Pyrazole Biomolecules as Cancer and Inflammation Therapeutics

**DOI:** 10.3390/molecules27248708

**Published:** 2022-12-08

**Authors:** Md. Jahangir Alam, Ozair Alam, Mohd. Javed Naim, Farah Nawaz, Ajay Manaithiya, Mohd Imran, Hamdy Khamees Thabet, Sultan Alshehri, Mohammed M. Ghoneim, Prawez Alam, Faiyaz Shakeel

**Affiliations:** 1 Medicinal Chemistry and Molecular Modelling Lab, Department of Pharmaceutical Chemistry, School of Pharmaceutical Education and Research, Jamia Hamdard, New Delhi 110062, India; 2 Department of Pharmaceutical Chemistry, Faculty of Pharmacy, Tishk International University, Erbil 44001, Kurdistan Region, Iraq; 3 Department of Pharmaceutical Chemistry, Faculty of Pharmacy, Northern Border University, Rafha 91911, Saudi Arabia; 4 Department of Chemistry, Faculty of Arts and Sciences, Northern Border University, Rafha 91911, Saudi Arabia; 5 Department of Pharmaceutical Sciences, College of Pharmacy, AlMaarefa University, Ad Diriyah 13713, Saudi Arabia; 6 Department of Pharmacy Practice, College of Pharmacy, AlMaarefa University, Ad Diriyah 13713, Saudi Arabia; 7 Department of Pharmacognosy, College of Pharmacy, Prince Sattam Bin Abdulaziz University, Al-Kharj 11942, Saudi Arabia; 8 Department of Pharmaceutics, College of Pharmacy, King Saud University, Riyadh 11451, Saudi Arabia

**Keywords:** pyrazole, biomolecules, anticancer, anti-inflammatory

## Abstract

Pyrazole, an important pharmacophore and a privileged scaffold of immense significance, is a five-membered heterocyclic moiety with an extensive therapeutic profile, viz., anti-inflammatory, anti-microbial, anti-anxiety, anticancer, analgesic, antipyretic, etc. Due to the expansion of pyrazolecent red pharmacological molecules at a quicker pace, there is an urgent need to put emphasis on recent literature with hitherto available information to recognize the status of this scaffold for pharmaceutical research. The reported potential pyrazole-containing compounds are highlighted in the manuscript for the treatment of cancer and inflammation, and the results are mentioned in % inhibition of inflammation, % growth inhibition, IC_50_, etc. Pyrazole is an important heterocyclic moiety with a strong pharmacological profile, which may act as an important pharmacophore for the drug discovery process. In the struggle to cultivate suitable anti-inflammatory and anticancer agents, chemists have now focused on pyrazole biomolecules. This review conceals the recent expansion of pyrazole biomolecules as anti-inflammatory and anticancer agents with an aim to provide better correlation among different research going around the world.

## 1. Introduction

**Scheme 1:**
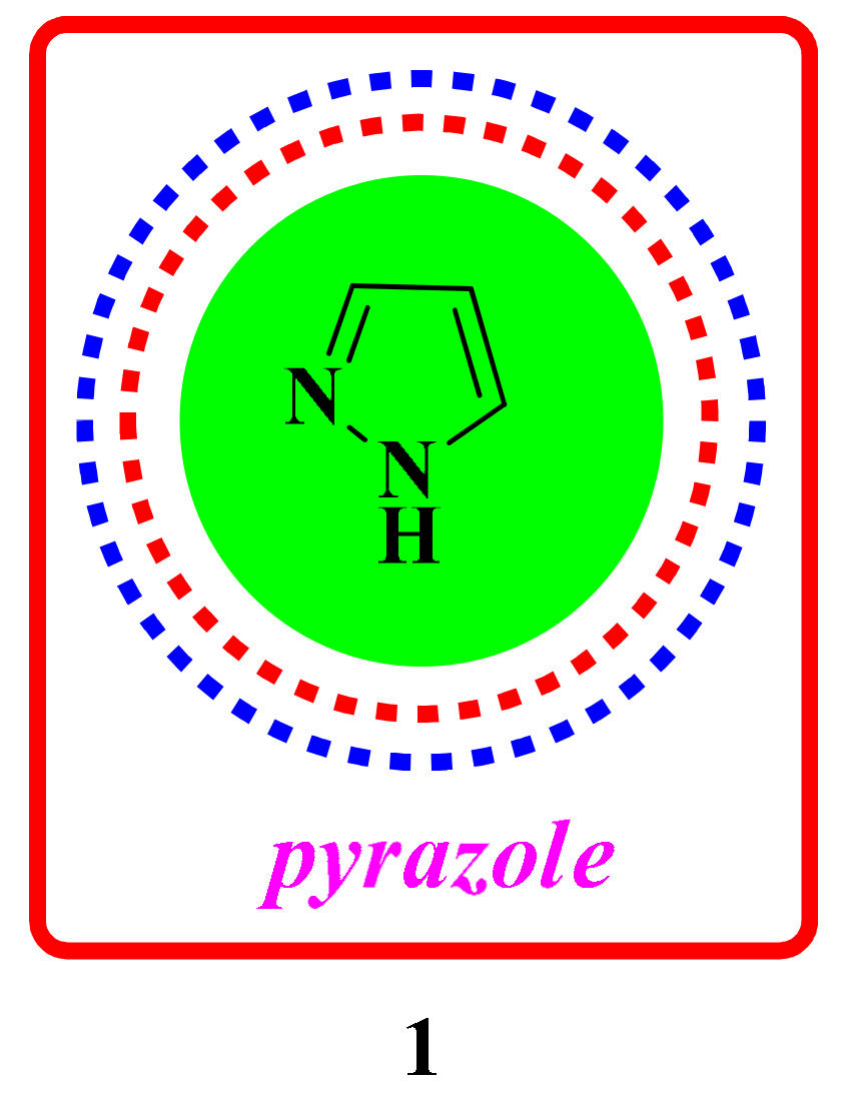
Five membered pyrazole moiety.

The pyrazole moiety is a heterocyclic ring system (five membered) with 3 C and 2 N in adjacent sites. In the year 1959, 1-pyrazolyl-alanine (natural pyrazole) was obtained from watermelons seeds [1,2]. Pyrazole compounds have an extensive past of applications, being used as herbicides, agrochemicals, and as active pharmaceutical agents. The current scenario of pyrazole moiety (Scheme 1) as a selective COX-2 inhibitor and anticancer agents has additionally emphasized their significance in pharmaceutical chemistry [3].

**Figure 1:**
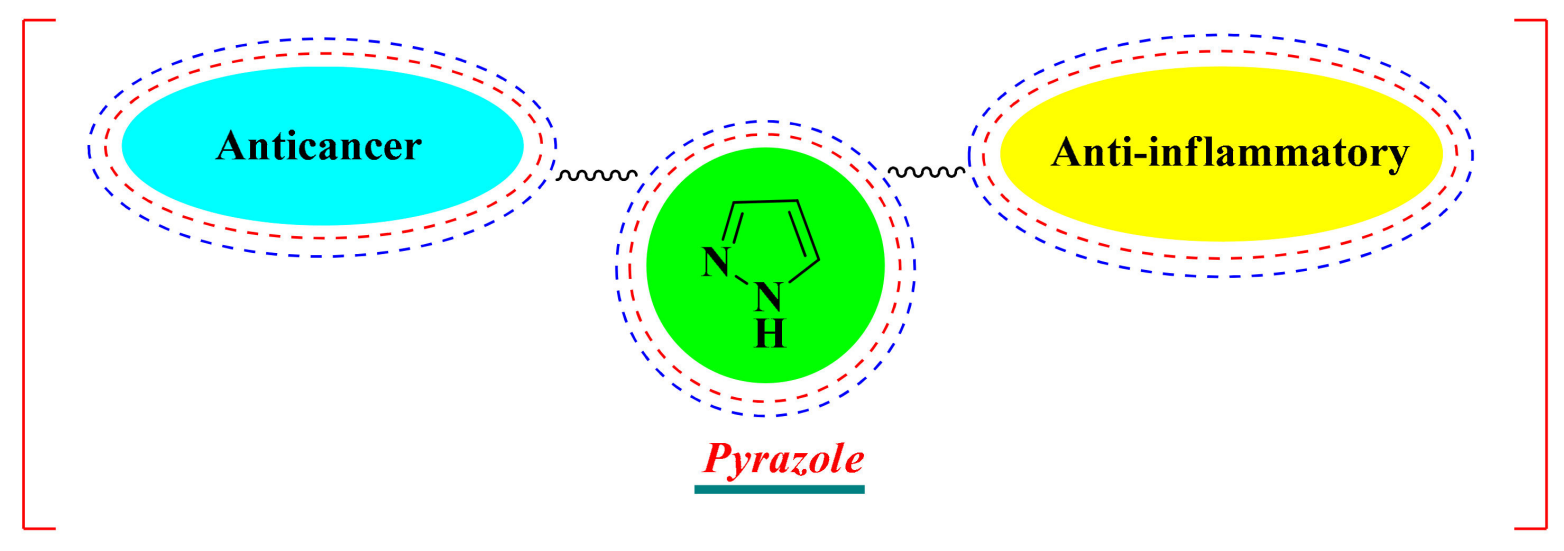
Pharmacological profile of pyrazole.

**Figure 2:**
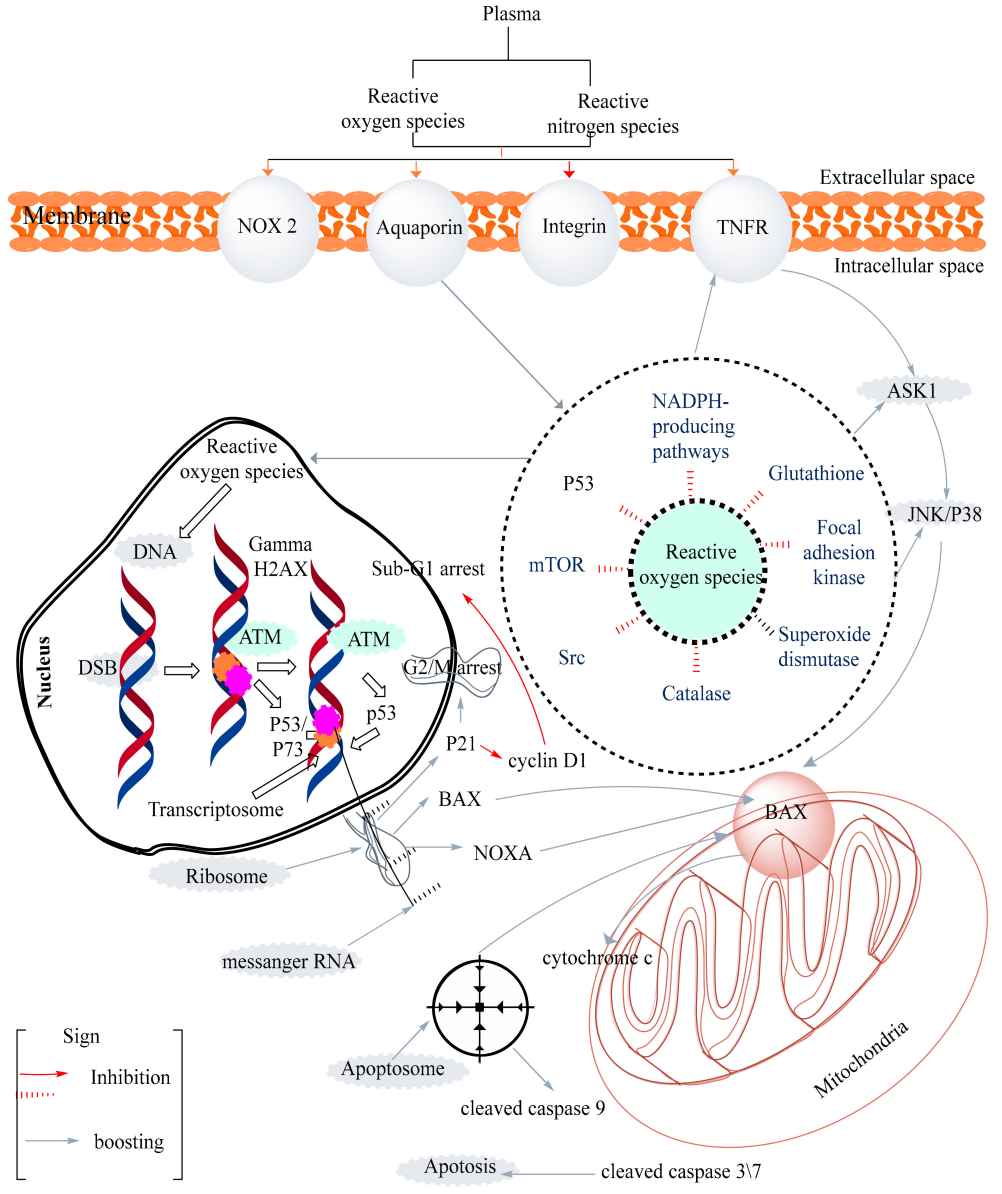
The mechanism of action of the anticancer activity pathway.

**Figure 3:**
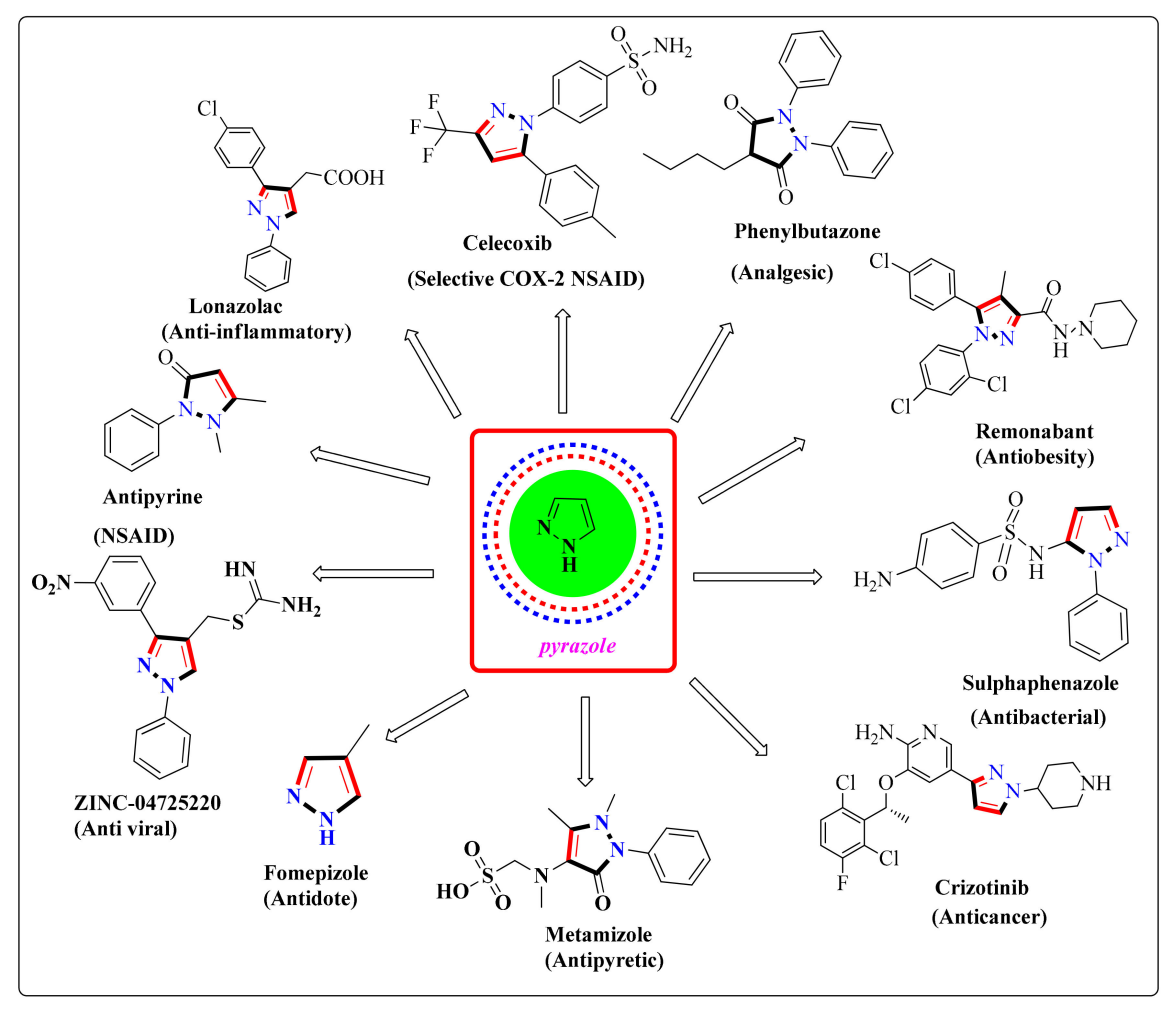
The pyrazole scaffold, exhibiting various pharmacological activities.

An organized observation of these groups of heterocyclic molecules exhibited that pyrazole-bearing therapeutic agents play an essential part in pharmaceutical sciences. The dominance of the pyrazole moiety in therapeutically potential lead compounds has encouraged the necessity for sophisticated and well-organized methods to prepare these heterocyclic compounds. The pyrazole moiety is shown to possess a broad range of biological profiles in the literature, mainly anticancer, anti-inflammatory, analgesic, antioxidant, anti-convulsant, anti-microbial, anti-mycobacterial, anti-amoebic, anti-depressant, hypotensive, and ACE inhibitors [4]. Several pyrazole-containing moieties have previously found their therapeutic application clinically, as NSAIDs such as celecoxib, lonazolac, tepoxaline, antipyrine (analgesic and antipyretic), phenylbutazone (in rheumatoid arthritis, osteoarthritis), novalgine, deracoxib, difenamizole, mepirizole, SC-560, crizotinib, pyrazofurin, and many more are already in the market [5]. Such drugs are listed in Table 1. In this updated review, our chief intention is to accentuate the anti-inflammatory and anticancer profiles displayed by the pyrazole moiety (Figure 1). The mechanism of action of anticancer activity are given in Figure 2. The different kinds of pharmacological activities of various compounds containing the pyrazole moiety are summarized in Figure 3.

## 2. Pyrazole Biomolecules as Cancer Therapeutics

Cancer is an uncontrolled abnormal cell growth [20] and a severe life-threatening condition globally [21,22]. It is a class of several illnesses [23] that are initiated to be deadly, followed by cardiovascular disorders in mortality and morbidity [24,25]. Although huge innovative and operative elucidations to cancer have been made, and quite clear margins in the management of cancer are exhibited, hereafter, it is estimated as the principle cause of mortality in the upcoming generation [26]. Presently, pharmaceutical companies are putting large amounts of money into the innovation and advancement of potential and safe anticancer drugs with fewer side effects [27,28]. Therefore, 1,3,4-trisubstituted pyrazole derivatives were prepared and evaluated for cytotoxic potential by Abadi et al., *N*-(1-{1-[4-nitrophen]-3-phephenyl-1*H*-pyrazol-4-yl}methylene)-2-chlorobenzohydrazide (**2**) showed satisfactory potential for MCF7, SF-268, and NCI-H460 cell lines with GI_50_, TGI_50_, and LC_50_ values of 3.79, 12.50, and 42.30 µM [29] (Refer to Table 2 for the structure of the compound). Bouabdallah et al., reported concerning *N*,*N*-bis[(3, 5-dimethylpyrazol-1-yl) methyl]aniline (**3**) and screened against Hep-2 and P815. These derivatives exhibited significant cytotoxic potential (IC_50_ = 3.25 mg/mL and 17.82 mg/mL) against Hep-2 and P815 cancer cell lines [30] (Refer to Table 2 for the structure of the compound). Wei et al., described ethyl-1-(2-hydroxy-3-aroxypropyl)-3-aryl-1H-pyrazole-5-carboxylate derivatives, among which, compound **4** was found to be most potent in countering A549 growth (IC_50_ = 26 µM) [31] (Refer to Table 2 for the structure of the compound). Xia et al., prepared 1-arylmethyl-3-aryl-1*H-*pyrazole-5-carbohydrazide and screened for antitumor activity. Compound **5** displayed significant cell apoptosis and potent antitumor activity with growth inhibitory properties (IC_50 =_ 49.85 μM) [32]. Fan et al., carried out synthesis of 1-(2′-hydroxy-3′-aroxypropyl)-3-aryl-1*H-*pyrazole-5-carbohydrazide derivatives and evaluated cytotoxicity against A549 cell lines. Compounds **6** and **7** brought about A549 cell line autophagy without causing apoptosis and 1-(3-(4-chlorophenoxy)-2-hydroxypropyl)-3-(4-chlorophenyl)-1*H-*pyrazole-5-carbohydrazide showed maximum autophagy in NCIH460 cells lines with IC_50_ of 48 μM and 32 μM at 48 h [33]. Abdel-Aziz et al., synthesized and screened compound **8,** which exhibited significant potential for Hep-2 cancer cell lines with IC_50_ = 0.74 mg/mL [34]. Zheng et al., described a new class of (E)-1-(4-tert-butylbenzyl-N′-(1-(5-chloro-2-hydroxyphenyl)ethylidene)-3-(4-chlorophenyl)-1*H-*pyrazole-5-carbohydrazides among which compound **9** offered IC_50_ = 0.28 µM and caused A549 cell line apoptosis [35]. Zheng et al., described some Oxime-linked pyrazole derivatives. Compound **10** indicated the highest potential for the A549 cancer cell line with in vitro cytotoxicity (IC_50_ = 14.5 µM) [36] (Refer to Table 2 for the structure of the compound). Newer derivatives of 3-(Biphenyl-2-yl)-5-(4-methoxyphenyl)-1*H-*prazole were investigated by Shaw et al., against NCI-H460, SW620, OVCA, and AGS cell lines, in which compound **11** revealed GI_50_ of 0.67, 0.89, 0.73, and 0.79 µM [37] (Refer to Table 2 for the structure of the compound). Poly-substituted pyrazole derivatives having antitumor potential were prepared by Rostom et al. The amino cyano pyrazole **12** showed GI_50_ 0.36 µM, TGI 8.78 µM, and LC_50_ MG-MID values of 69.3 µM, whereas tricyclic-napthopyrazole-containing derivative **13** showed GI_50_ = 0.08 µM, TGI 30.9 µM, and MG-MID values of 93.3 µM [38] (Refer to Table 2 for the structure of the compound). Newer pyrazolic compounds were developed and assessed for antitumor activity by Insuasty et al., Compounds **14** and **15** appeared most active with GI_50_ values ranging between 0.04 to 11.4 µM against K-562, UO-31, SR, and HOP-92 cancer cell lines [39]. Nitulescu et al., prepared different substituted pyrazole derivatives in which compound **16** appeared potent with a mean log of GI_50_ = −5.75 [40]. Zhang et al., reported 3-(1*H-*indole-3-yl)-1*H-*pyrazole-5-carbohydrazide derivatives; where compound **17** and **18** exhibited important antiproliferative potential for HepG-2 with an IC_50_ of 0.71 μM, BT474 with an IC_50_ value of 1.39 μM, and BGC823 cell lines with an IC_50_ value of 0.71 μM compared to the standard 5-fluorouracil (5-FU). The results validated that both derivatives arrested cell cycle at S phase [41] (Refer to Table 2 for the structure of the compound).

El-Gamal et al., prepared 3-(3-chloro-5-hydroxyphenyl)-*N*-(2-(4-methylpiperazin-1-yl)ethyl)-4-(pyridine-4-yl)-1*H*-pyrazole-1-carboxamide derivatives, in which compound **19** was the most powerful anticancer agent for A375 cell lines with IC_50_ = 4.2 μM [42] (Refer to Table 3 for the structure of the compound). *N*-((1,3-diphenyl-1*H*-pyrazol-4-yl)methyl) aniline-containing derivatives were explored by Huang et al., who assessed them for anticancer potential and cyclin-dependent kinase-2 (CDK2) inhibitory properties in vitro, with an IC_50_ of 0.98 ± 0.06μM. Compound **20** reflected excellent anticancer potential against MCF-7 with IC_50_ values of 1.88 ± 0.11 and B16-F10 cancer cell lines with IC_50_ = 2.12 ± 0.15 μM [43]. Compound **21,** displaying significant anticancer efficacy for HCT116 (IC_50_ = 0.39 ± 0.06 μM) and MCF-7 (IC_50_ = 0.46 ± 0.04 μM) cell lines, was reported by Li et al., and displayed inhibition of Aurora-A kinase with IC_50_ = 0.16 ± 0.03 µM [44]. Novel derivatives of the pyrazole moiety were prepared by Mohareb and Kumar et al., in which compounds **22** and **23** were screened against the MCF7 cancer cell line with IC_50_ = 0.01 µM, NCI-H460 with IC_50_ = 0.03 µM and SF-268 with IC_50_ = 31.5 µM against doxorubicin as the standard drug [27,45] (Refer to Table 3 for the structure of the compound). Sun et al., prepared 1,3-diphenyl-N-(phenylcarbamothioyl)-1*H-*pyrazole-4-carboxamide derivatives and assessed for CDK inhibition potential. Compound **24** caused inhibition of CDK2 (IC_50_ = 25 nM) and arrest of Go/G1 phase in A549 cancer cell lines in a dose reliant fashion [46]. Inceler et al., evaluated 1, 3-diarylpyrazole containing derivatives for anticancer potential among which compound **25** displayed GI_50_ values of 25.2 ± 3.2 and 28.3 ± 1.53 µM, against Raji and HL60 cancer cell lines [47] (Refer to Table 3 for the structure of the compound). Gamal et al., prepared newer pyrazole derivatives of which compound **26** displayed potential anticancer results against A375 cell line [48]. Xu et al., evaluated *N*-arylpyrazoles on Bel-7402, HL-60, BGC-823 and KB among which compound **27** caused significant inhibition of Bel-7402 cells (1.5 times) as compared to established drug cisplatin [49]. Koca et al., prepared of 4-benzoyl-1,5-diphenyl-*N*-(substitutedphenylcarbamothioyl)-1*H*-pyrazole-3-carboxamide derivatives through one pot synthesis and assessed for anticancer potential on HepG2, Jurkat, DLD-1, human T cell lymphoblast cancer cell lines. Compound **28** exhibited significant potential to be considered for inhibition of kinase [21] (Refer to Table 3 for the structure of the compound). Zheng et al., developed pyrazole linked benzimidazole derivative **29** and evaluated on U937, K562, HT29 and A549 cancer cell lines, LoVo and in vitro inhibition of Aurora A/B kinase [50]. 5-phenyl-1*H-*pyrazol derivatives were prepared and assessed for BRAF (V600E) inhibition. Compound **30** displayed significant inhibition with an IC_50_ = 0.19 µM as reported by Dong et al.; anti-cancer assays showed many derivatives displaying maximum anticancer potential against WM266.4 and A375 with IC_50_ = 1.50 to 1.32 µM, i.e., equivalent to vemurafenib [51] (Refer to Table 3 for the structure of the compound).

Mohareb et al., assessed the antitumor potential accompanied with pyrazole-containing derivative 31 for NCI-H460, MCF-7, and SF268 cell lines [52] (Refer to Table 4 for the structure of the compound). Nakao et al., prepared newer 1-(1H)-5-methylbenzimidazol-2-yl)-4-benzyl-3-methyl-1*H-*pyrazol-5-ol derivatives, among which compound **32** exhibited potential PCA-1/ALKBH3 inhibitory activity as an anti-prostate-cancer molecule [53]. Xing et al., developed novel pyrazole hydrazide derivative **33** that exposed significant activity on B16-F10 and MCF-7 cancer cell lines with IC_50_ = 0.49 ± 0.07 µM and 0.57 ± 0.03 µM [54]. In 2014, Aydın et al., prepared pyrazole-biphenyl derivatives in which compound **34** reflected 69.95% inhibition and apoptosis of K-562 cells. [55] (Refer to Table 4 for the structure of the compound). Cankara et al., prepared pyrazole containing amide derivatives and determined the anticancer potential against Huh-7, HCT-116, and MCF-7 cell lines through a sulforhodamine B assay [56]. Compound **35** showed promising cytotoxicity against HCT-116, Huh-7, and MCF-7 cell lines with IC_50_ = 1.1 µM, 1.6 µM, and 3.3 µM with arresting cell cycle at the SubG1/G1 phase. Kumar et al. [57] synthesized pyrazole-arylethanone derivatives under green conditions and evaluated them for cytotoxicity on HepG2, HCT116, and CDK2 and EGFR cell lines. Compound **36** showed cytotoxicity to all cell lines except SKOV3. On CDK2 and EGFR cell lines, compound **36** and **37** presented a similar potential in comparison to carboplatin. Pyrazole carboxamide derivatives were prepared by Lu et al., under moderate reaction conditions and assessed for anticancer activity. DNA binding interaction and kinase inhibition were examined to understand the mechanisms [58] (Refer to Table 4 for the structure of the compound). The anticancer assessment displayed good inhibition against tHCT116 and HepG2 and decreased inhibition of kinase by compound **38** with a supreme binding affinity towards DNA (Kpym-5) = 1.06 × 10^5^ M^−1^). Zhang et al., reported pyrazole-5-carboxamide-containing derivatives and assessed them for anticancer potential against A549 cell lines [59]. Compound **39** yielded significant inhibition at a concentration of 10 µM. Yao et al., prepared pyrazole derivatives showing disubstitution at 1 and 3 and evaluated their anticancer potential against BGC823, Hela, MCF7, A549, Molt-4, U937, K562, and HT1080 [60] (Refer to Table 4 for the structure of the compound). Compound **40** presented acceptable selectivity and anticancer potential against class I and IIb HDAC isoforms. Wen et al., prepared pyrazole-containing derivatives and evaluated for histone deacetylase inhibition. Compound **41** confirmed noteworthy antitumor potential in a xenografted model of HCT-116 [61]. Zhao et al., developed the excellent pyrazole derivative **42,** which showed its anticancer potential with IC_50_ = 0.12 µM and 0.16 µM on WM 266.4 and MCF-7 cell lines [62] (Refer to Table 4 for the structure of the compound). Kamal et al., reported a novel series of pyrazole derivatives, among which compound **43** displayed noteworthy cytotoxicity [63]. Hu et al., synthesized some novel compounds, in which compound **44** showed considerable inhibition for BCR-Bal kinase (IC_50_ = 14.2 nM) [64] (Refer to Table 4 for the structure of the compound). Nitulescu et al., prepared novel pyrazole-containing derivatives and investigated them for anticancer potency. Compound **45** appeared as a potential anticancer agent [65] (Refer to Table 4 for the structure of the compound). Newer substituted pyrazole derivatives were prepared by Rai et al., and assessed for anticancer activity. Compound **46** exhibited equivalent anticancer potential compared to rug doxorubicin [24] (Refer to Table 4 for the structure of the compound). Nitulescu et al., performed ultrasound-assisted synthesis of pyrazole derivatives and evaluated them for anticancer potential. Compound **47** appeared as a strong and potential anticancer agent [66] (Refer to Table 4 for the structure of the compound).

Khloya et al., developed sulfonamide derivative **48,** which was found to cause better inhibition of the carbonic anhydrase enzyme against acetazolamide compared to the standard drug [67]. Tao et al., synthesized pyrazole derivatives in which compound **49** appeared to be the most significant anticancer compound against EGFR and HER-2 tyrosine kinase with IC_50_ values of 0.26 µM and 0.20 µM [68] (Refer to Table 5 for the structure of the compound). Reddy et al., designed some pyrazole-containing derivatives and evaluated them for anticancer activity. Compound **50** prompted significant inhibition against MCF-7, A549, and HeLa with IC_50_ values between 0.83–1.81 µM [69] (Refer to Table 5 for the structure of the compound). Pyrazole–oxindole conjugates were developed and evaluated for anticancer potential and tubulin polymerization inhibition by Kamal et al., among which compound **51** significantly inhibited tubulin assembly [70] (Refer to Table 5 for the structure of the compound). Shi et al., prepared novel pyrazole derivatives of which compound **52** appeared as a potential anticancer agent [71]. Ali et al., prepared imidazo[2,1-b] thiazoles-linked pyrazole derivatives and evaluated them for antitumor potency. In vitro results revealed the potential of compound **53** against renal UO-31 and CNS SNB-75 cell lines [72] (Refer to Table 5 for the structure of the compound). Reddy et al., designed and synthesized 1*H-*pyrazolo-[4,3-d]pyrimidin-7(6H)-ones and assessed it for anticancer potential. Compound **54** showed potential anticancer results against HeLa, CAKI-I, PC-3, MiaPaca-2, and A549 cancer cell lines [73] (Refer to Table 5 for the structure of the compound). Zhang et al., reported on pyrazolyl hydroxamic acid derivatives and assessed their potential against A549 cancer cell lines to determine their anti-proliferative activity, in which compound **55** appeared as the most potent compound [59]. Imidazo[1,2-b] pyrazole derivatives were prepared by Grosse et al., and evaluated through MTT assay. Compound **56** showed significant inhibition with IC_50_ below 5 µM against human and murine cancer cell lines [74]. Pirol et al., designed and prepared 5-(p-tolyl)-1-(quinolin-2-yl)pyrazole-3-carboxylic-acid-based amide derivatives and assessed them for anticancer potential. Compound **57** exhibited the most potential [56]. Jin et al., designed pyrazole-based 4-([1, 2, 4]triazolo[1,5-a]pyridine-6-yl)-5(3)-(6-methylpyridin-2-yl)imidazole derivatives and evaluated them for ALK5 inhibition, of which compound **58** yielded the most significant results [75]. Park et al., prepared trisubstituted pyrazole-containing derivatives and assessed them for ROS1 kinase inhibition. Compound **59** exhibited five times greater potential against crizotinib [76] (Refer to Table 5 for the structure of the compound). Wang et al., prepared 5-phenyl-1*H-*pyrazole analogues encompassing a niacinamide ring and assessed them for antiproliferative potential. Compound **60** reflected potential cytotoxicity with IC_50_ = 2.63 µM and 3.16 µM against WM 266.64 and A375 [77]. Kamal et al., developed 4b-amidopodophyllotoxin heteroaromatic conjugates and assessed them for anticancer potential. Compound **61** revealed excellent antitumor potential against A549 cell lines [78]. Novel pyrazole-linked carbothioamide derivatives were prepared by Zhu et al., and assessed for anticancer potential. Compound **62** exhibited the most cytotoxic potential with IC_50_ = 6.51 µM against Raji cell lines [79]. Zheng et al., developed pyrazole-linked benzimidazole conjugates and assessed them for Aurora A/B kinase inhibition potential and anticancer activity. Compound **63** displayed significant inhibition [50]. Compound **64** was prepared by Miyamoto et al., through hybridization and optimization of different imidazo[1,2-b]pyridazines, which caused the inhibition of VEGFR2 kinase with IC_50_ = 0.95 nM. Furthermore, it inhibited VEGF-induced proliferation of human umbilical vein endothelial cells with IC_50_ = 0.30 nM [80] (Refer to Table 5 for the structure of the compound).

According to the molecular modeling methodology, Bavetsias et al., reported on imidazo[4,5-b] pyridine linked pyrazole derivatives and assessed them for selective Aurora-A kinase inhibition. Compound **65** caused inhibition of Aurora-A kinase with IC_50_ = 0.067 µM [81] (Refer to Table 6 for the structure of the compound). Sun et al., synthesized pyrazole-linked thiourea derivatives and explored them for CDK2 inhibition. Compound **66** appeared to have the most potential as a CDK2 inhibitor with IC_50_ = 25 nM, as well as inhibited proliferation of H460, MCF-F, and A549 in the range of 0.75–4.21 µM [47]. Li et al., prepared *N*-1,3-triphenyl-1*H-*pyrazole-4-carboxamide derivatives and assessed them for anticancer potential and binding against Aurora-A kinase. Compound **67** showed significant inhibition for HCT116 (IC_50_ = 0.39 ± 0.06 µM) and MCF-7 (0.46 ± 0.04 µM) cell lines and IC_50_ = 00.16 ± 0.03 µM against Aurora-A kinase [44] (Refer to Table 6 for the structure of the compound). Liu et al., prepared pyrazole-linked peptidomimetics, in which compound **68** reflected the potential inhibition of A549 cell lines with IC_50_ = 36.12 µM [82]. Lv et al., synthesized 5-benzyl-2-phenylpyrazolo[1,5-a]pyrazin-4,6(5H, 7H)-dione derivatives, in which compound **69** selectively inhibited H322 cell lines, along with the induction of apoptosis [83] (Refer to Table 6 for the structure of the compound). Bai et al., developed novel 1-acyl-3-amino-1,4,5,6-tetrahydropyrrolo[3,4-c]pyrazole derivatives and assessed them for antitumor potential against the HCT-116 cancer cell line, in which compound **70** showed most the potent results and was further designated for future cytotoxicity estimation against many other cancer cell lines. This compound transcended (R)-roscovitine by 4–28 times in terms of its potency. Moreover, this compound was also screened against twelve kinases and its interaction with CDK5 and glycogen synthase kinase-3b [84] (Refer to Table 6 for the structure of the compound). Newer pyrazolo[3,4-d]pyrimide-containing derivatives were prepared by Huang et al., where compound **71** displayed cytotoxicity against NCI-H226 and NPC-TW01 cell lines with GI_50_ = 18–30 µM [85] (Refer to Table 6 for the structure of the compound). Strocchi et al., developed pyrazole analogues and assessed them against HCC and SNU449 cancer cell lines. Compound **72** appeared most potent with IC_50_ = 50–100 µM and effectively blocked cell cycle progression and induced apoptosis [86]. Metwally et al., reported pyrazole and pyrazolotriazine derivatives and tested them for anticancer potential, in which compound **73** exhibited significant results [87]. Novel farnesyltransferase inhibitors containing phenothiazine were developed by Baciu-Atudosie et al., and determined against NCI-60 cancer cell lines. Compound **74** revealed significant cytostatic activity against HCT-116, SKMEL-5, and LOX IMVI cancer cell lines [88]. Bondock et al., prepared hybrid 1,3,4-oxadiazole derivatives and assessed them for anticancer potential. Compound **75** appeared to be the most potent against four cell lines [89]. Deslandes et al., prepared garnulatimide- and isogranulatimide-containing pyrazole derivatives and evaluated their anticancer activity, in which compound **76** displayed the highest potential results with an IC_50_ value of 11 ± 4 µM [90] (Refer to Table 6 for the structure of the compound). Puthiyapurayil et al., prepared novel S-substituted-1,3,4-oxadiazole linked *N*-methyl-4-(trifluoromethyl)phenylpyrazole derivatives combinatorial library and evaluated them for cytotoxic activity through an MTT assay. Compound **77** showed potential anti-proliferative results for MCF-7 cell lines with IC_50_ = 15.54 µM [91] (Refer to Table 6 for the structure of the compound). El-borai et al., prepared pyrazolopyridine derivatives through microwave irradiation and evaluated them for antitumor activity, in which compound **78** showed the maximum potency [92] (Refer to Table 6 for the structure of the compound). Ferrocenyl pyrazole-linked chiral aminoethanol derivatives were reported by Shen et al., and assessed for H322 lung and A549 cancer cell line inhibition, in which compound **79** displayed the maximum anticancer potential [93]. Abd El Hamid et al., prepared novel pyrazolopyrimidine derivatives, where compound **80** yielded the most significant anticancer activity with an IC_50_ value of 7.5 nM [94]. Vujasinovic et al., prepared pyrazole derivatives from different routes, among which compound **81** revealed maximum potency with a significant safety profile [95] (Refer to Table 6 for the structure of the compound).

Yamamoto et al., synthesized newer pyrazole containing derivatives and investigated them for anticancer potential. Compound **82** showed most the potential antiproliferative results against a CRPC model of LNCap-hr cell lines [96] (Refer to Table 7 for the structure of the compound). Newly synthesized pyrazole-based imidazopyridine derivatives were determined for antitumor potential by Bavetsias et al., in which compound **83** caused the most significant Aurora kinases inhibition [97]. Hanan et al., prepared newer pyrazolopyrimidine derivatives and assessed them for inhibition of Janus Kinase. Compound **84** appeared to be the derivative with the most potential with an IC_50_ = 7.4 nM [98]. Newhouse et al., prepared several non-Oxime containing pyrazole derivatives and assessed their anticancer activity. Compound **85** demonstrated appreciable B-Raf kinase inhibition [99] (Refer to Table 7 for the structure of the compound). Newer pyrazole derivatives were prepared by Zheng et al., and evaluated for anticancer activity. Compound **86** potentially inhibited H322 carcinoma and A549 cancer cell lines [100]. Jin et al., prepared 1-substituted-3-(6-methylpyridin-2-yl)-4-([1,2,4]triazolo[1,5-a]pyridin-6-yl)pyrazole derivatives and assessed them for cytotoxic potential. Compound **87** appeared to be the derivative with the most potential with IC_50_ = 0.57 nM [101]. Pyrazole linked molecules were prepared by Maggio et al., and investigated for antiproliferative activity. Compound **88** appeared to be the most significant anticancer agent with growth inhibition (GI_50_) ranging from 14–18 µM against HL60 and K562 [102]. Christodoulou et al., prepared trisubstituted pyrazole derivatives and assessed them for anti-angiogenic potential. Compound **89** appeared to be the most potent with IC_50_ = 12 μM for 89a and 25 ± 1.4 μM for 89b and inhibited MCF-7 with IC_50_ = 26 ± 2.2 μM and Hela with IC_50_ = 37 ± 2.6 μM carcinoma cells in vitro [103]. Lv et al., prepared pyrazole derivatives and assessed them for EGFR kinase inhibition. Compound **90** appeared most potential with IC_50_ of 0.07 μM, in comparison to erlotinib [104] (Refer to Table 7 for the structure of the compound). Niculescu-Duvaz et al., reported novel 1-arylmethyl-3-aryl-1*H*-pyrazole-5-carbohydrazide derivatives and assessed them for A549 cell line inhibition and induction of apoptosis. Compound **91** was found to be the most potent with significant logP (3.12–4.94) values [105] (Refer to Table 7 for the structure of the compound). Zheng et al., synthesized 3-aryl-1-(4-tert-butylbenzyl)-1*H-*pyrazole-5-carbohydrazide hydrazone derivatives and assessed them for A549 cell inhibition. Compound **92** exhibited the maximum inhibition of lung cancer cell lines [35]. Newer 3, 4-disubstituted pyrazole derivatives were prepared by Lin et al., and assessed for cyclin-dependent kinase inhibitory and anticancer activity on human cancerous cells [106]. Compound **93** was found to be the most effective. A novel series of 7-phenyl-7*H-*pyrazolo[4,3-e]-[1,2,4,]triazolo[1,5-c]pyrimidine-2-thione derivatives was prepared by Ghorab et. al. and assessed for Ehrlich Ascites Carcinoma cell lines against doxorubicin. Compound **94** yielded the most potent results [107]. Radek et al., reported pyrazolo[4,3-d]pyrimidine derivatives and assessed them for CDK2 inhibition, in which compound **95** appeared to be the most potent [108]. Dawood et al., prepared 2-(4-(pyrazol-4-yl)thiazol-2-ylimino)-1,3,4-thiadiazole derivatives, where compound **96** appeared as the best potential antiproliferative agent [109]. Singla et al., prepared newer pyrazolo[3,4-d]pyrimidine linked 4-(1*H-*benzimidazol-2-yl)-phenylamine derivatives and assessed their antitumor potency against NCI-60 cancer cell lines. Compound **97** exhibited the best potential inhibition at 10 µM concentration with average GI_50_ values of 1.30 μM [110]. Krystof et al., prepared 4-arylazo-3,5-diamino-1*H-*pyrazole derivatives and investigated them for anticancer activity and CDK inhibition. Compound **98** appeared as the best potential derivative with significant selectivity and cellular effects [111] (Refer to Table 7 for the structure of the compound). Balbi et al., developed pyrazole scaffolds and assessed them against A2780 cells, A549 cells, and P388 cell lines. Compound **99** significantly bound to α- and β-tubulin and caused molecular distortion, leading to disassembly of the microtubules [112] (Refer to Table 7 for the structure of the compound).

Raquib et al., synthesized ethyl-4-(3-(aryl)-1-phenyl-1*H-*pyrazol-4-yl)-2-Oxo-6-(pyridin-3-yl)cyclohex-3-enecarboxylate derivatives and evaluated them for topoisomerase-IIa inhibition and anticancer potential against MCF-7, NCI-H460, HeLa, and HEK-293T. Compound **100** showed 70.82% topoisomerase-IIa inhibition (100 µM) and significant anticancer potential against cancer cell lines with IC_50_ values of 7.01 ± 0.60 µM (HeLa), 8.55 ± 0.35 µM (NCIH460), and 14.31 ± 0.90 µM (MCF-7) [113] (Refer to Table 8 for the structure of the compound). Wang et al., prepared indolyl-substituted 1,4,6,7-tetrahydropyrano [4,3-c] pyrazole derivatives and assessed them for anticancer activity by MTT assay on MCF-7, HGC-27, PC-3, and EC-109 cell lines. Compound **101** displayed three times more activity than 5-fluorouracil [114]. Minu et al., reported mono- and di-substituted 2,3,3a,4,5,6-hexahydrocyclopenta[c]pyrazole derivatives and assessed them for anticancer potential on MCF-7 and A589 cell lines. Compound **102** displayed the best potential results with IC_50_ = 15.6 µM and 19.8 µM against MCF-7 and A589 cell lines [115]. Hafez et al., synthesized [4-amino-3-(4-chlorophenyl)-1*H-*pyrazol-5-yl](3,5-dimethyl-1*H-*pyrazol-1-yl)-methanone derivatives and assessed them for anticancer potential. Compounds **103** showed greater anticancer potential activity than doxorubicin as the standard drug [116] (Refer to Table 8 for the structure of the compound). Lv et al., synthesized newer (E)-1,3-diphenyl-1*H-*pyrazole-containing derivatives bearing the O-benzyl Oxime group and evaluated them for immunosuppression. Compound **104** displayed the best potential inhibition with IC_50_ = 1.18 µM for lymph node cells and 0.28 µM for PI3K [117] (Refer to Table 8 for the structure of the compound). Reddy et al., developed (*N*-((1-benzyl-1*H-*1,2,3-triazol-4-yl)methyl)-1,3-diphenyl-1*H-*pyrazole-4-carboxamide derivatives and investigated them for CDK (I/II) inhibition. Compound **105** exhibited significant inhibition on MIAPaCa-2, MCF-7, and HeLa cell lines in the range of 0.13–0.7 µM (GI_50_) against nocodazole (0.81–0.95 µM) [118]. Yoon et al., reported a new and specific RET inhibitor as 5-aminopyrazole-4-carboxamide lead molecules to improve the metabolic stability of the pyrazolopyrimidines. Compound **106** revealed potent results against gatekeeper mutant (IC_50_ = 252 nM) and wild-type RET (IC_50_ = 44 nM) [119] (Refer to Table 8 for the structure of the compound). Thaher et al., reported 3-(4-fluorophenyl)-4-(pyridin-4-yl)-1-(aryl)-1*H-*pyrazol-5-amine as p38α inhibitors, of which compound **107** showed the maximum potential with IC_50_ in nanomolar range on B-Raf V600E, Src, B-Raf wt, VEGFR-2, and EGFRs, establishing it as an upright lead for novel anticancer potential [120]. Al-Suwaidan et al., prepared 1-(4-chlorophenyl)-4,4,4-trifluoro-2-(2-(4-methoxyphenyl)hydrazone)butane-1,3-dione derivatives and assessed them for in vitro antitumor activity. Compound **108** showed potential results with a GI_50_ value of 6.61 µM, TGI value of 42.66 µM, and LC50 value of 93.33 µM in comparison to 5-fluorouracil [121] (Refer to Table 8 for the structure of the compound). Pyrazoline-based hydroxamic acid derivatives were synthesized and assessed for anticancer potential. Compound **109** was recognized from a library of derivatives and showed a significant inhibitory effect against cancer cell types. When compared to side chains in the meta position, hydroxamate side chains in the ortho position exhibited greater biological activity. When compared to the reference drug Bestatin (IC_50_ > 500 μM against all cancer cell lines), compound **109** substituted with formamide demonstrated potent anti-proliferative activity (IC_50_ = 54.2 ± 4.9 μM, 116.5 ± 8.2 μM, 18.7 ± 1.6 μM, 156.0 ± 7.5 μM, 65.0 ± 2.8 μM, and 65.0 ± 2.8 μM against U93, PLC/PRF/5, K562, ES-2, PC-3, and HepG2). K562 was the most susceptible to pyrazoline-based compounds among all these cell lines. Additionally, compound **109** inhibited aminopeptidase N (APN) with IC_50_ = 0.16 ± 0.02 μM, which was higher than one order of scale lower than bestatin (IC_50_ = 9.4 ± 0.5 μM) [122] (Refer to Table 8 for the structure of the compound). Furthermore, 1,2,4-oxadiazole merged 1,2,3-triazole-pyrazole derivatives were prepared and tested by MTT assay against DU-145, PC3, MCF-7, and A549 cancer lines using etoposide as the standard drug. The anticancer activity of compound **110** (with the 3,5-dinitro group on the phenyl ring) was found to be the strongest, with IC_50_ = 0.01 ± 0.008 µM, 0.45 ± 0.023 µM, 0.081 ± 0.0012 µM, and 1.77 ± 0.33 µM against PC3, A549, MCF-7, and DU-145 cell lines. Compared to etoposide (IC_50_ = 1.36 ± 0.22 µM, 0.11 ± 0.066 µM, 0.13 ± 0.072 µM, and 1.50 ± 0.19 µM against A549, MCF-7, PC-3, and DU-145), the compound substituted with 4-nitro substituent on the phenyl ring connected to the oxadiazole skeleton showed decent anticancer activity (IC_50_ = 3.08 ± 0.135 µM, 1.97 ± 0.45 µM, 2.39 ± 1.56 µM, 2.11 ± 0.024 µM against A549, DU-145, PC-3, and MCF-7, respectively). Substitution of the 3,5-dinitro group with 4-chloro, 3,5-dimethoxy, or 4-methoxy group resulted in decreased activity; however, the replacement of the 3,5-dinitro group with a 4-Bromo, electron-rich group (3,4,5-trimethoxy) resulted in reasonable anticancer activity [123] (Refer to Table 8 for the structure of the compound). Pyrazoles incorporating thiazole derivatives were synthesized, and their bioactivity as a target for leukemia cancer was investigated. The most active compounds were pyrazole derivatives, with rising activity seen for pyrazole derivatives including a thiazole ring. Compound **111** with *N*-(3-methoxy-2-hydroxybenzal)-3-substituted(p-chlorophenyl)-4-cyano-5-oxopyrazol-1-thiocarboxamide against HL-60 was the most effective, with an IC_50_ = 1.35 µM, in comparison to Dox’s with IC_50_ = 2.02 µM. When compared with the untreated control, compound **111** enhanced the G2/M phase from 11.05% to 39.22%. Furthermore, in comparison to the untreated control, compound **111** can enhance the proportion of late apoptosis from 0.31 to 4.31%. With an IC_50_ value of 56.04 nM, compound **111** inhibited Topo II activity more effectively than etoposide (IC_50_ = 41.11 nM). The cytotoxic action of compound **111** is mostly attributable to its high DNA Topo II inhibition activity, according to this finding [124] (Refer to Table 8 for the structure of the compound). Novel pyrazole–pyrazoline-hybrid-containing derivatives were prepared and assessed as cancer-associated selective COX-2 inhibitors. Compound **112** indeed had a greater COX-2 selectivity index, as well as strong anticancer potential towards A549, SiHa, HepG2, and COLO205, (IC_50_ = 4.94 ± 0.05 µM, 4.94 ± 0.05 µM, 2.09 ± 0.01 µM, and 4.86 ± 0.01 µM) compared to 5-fluorouracil as a standard drug (IC_50_ = 2.08 ± 0.01 µM, 5.78 ± 0.04 µM, 4.35 ± 0.01 µM, 4.00 ± 0.01 µM, 19.01 ± 0.21 µM, respectively, against MCF-7, SiHa, A549, and COLO205, HepG2). The compound with no substitutions on ring B was revealed as being the most active (compound **112**). The activity reduced as the electronegativity of ring B increased. Increases in electronegativity on ring B and electro positivity on ring C were observed to have a positive influence on activity. The study found that methoxy-containing compounds were more suited for in vitro anticancer activities (compound **112**). The activity reduced as the electronegativity of ring-C rose. Because the compounds were more specific for the COX2 enzyme, it was possible to conclude that they showed anticancer effects via COX2 inhibition [125] (Refer to Table 8 for the structure of the compound).

## 3. Pyrazole Biomolecules as Inflammation Therapeutics

Inflammation is a physiological response to destructive stimuli encompassing blood vessels, immune cells, and chemical mediators (TNF-α, NO, IL-6) [126,127]. Pain is stressful and a horrible response due to injury in tissues or organs [128,129]. NSAIDs are a significant group of drug molecules recommended to cope with inflammatory conditions and to mitigate pain [130,131]. Nevertheless, excessive usage of NSAIDs frequently results in numerous adverse conditions such as gastrointestinal mucosal damage, renal toxicity, intolerance, and hepatotoxicity [132,133]. Future advancement/improvement of the therapeutic potential of anti-inflammatory and analgesic drugs (NSAIDs) lacking unwanted effects is still to be revealed by pharmaceutical scientists [134,135]. That is why several pyrazole bearing agents have also been established by chemists and druggists. Balbi et al., synthesized 5-(2,6,6-trimethyl-2-cyclohexen-1-yl)ethenyl-1*H-*pyrazolein derivatives, among them, compound **113** appeared to be a potent inhibitor of neutrophil chemotactic responsiveness in comparison to diclofenac [136] (Refer to Table 9 for the structure of the compound). Hall et al., developed methylene linked with pyrazoles, in which compound **114** appeared as a potent EP_1_ receptor antagonist [137]. Bandgar et al., prepared diaryl pyrazole derivatives and assessed them for anti-inflammatory potential against TNF-α and IL-6. Compound **115** showed IL-6 inhibition (42% at 10 μM) compared to flavopiridol (72–87% inhibition at 0.5 μM) and dexamethasone (85% inhibition at 1 μM) [138] (Refer to Table 9 for the structure of the compound). Abdel-Hafez et al., reported pyrazole-NO hybrid derivatives and assessed them for release of NO and their anti-inflammatory potential. Compound **116** displayed the maximum NO release and significant anti-inflammatory potential in comparison to indomethacin. [139] (Refer to Table 9 for the structure of the compound). Furthermore, 3-(4-methoxyphenyl)-*N*-propyl-5-(1*H-*pyrrol-2-yl)-1*H-*pyrazole-1-carbothioamide derivatives were developed by Gokhan-Kelekci et al., Compound **117** exhibited anti-inflammatory and analgesic potential [140]. Bis(3-aryl-4,5-dihydro-1*H-*pyrazole-1-thiocarboxamides) derivatives were prepared by Barsoum et al., and assessed for anti-inflammatory activity. Compound **118** appeared to be the best potential derivative in comparison to indomethacin [141]. Chandra et al., reported newer pyrazole derivatives and determined their anti-inflammatory and analgesic potential. Compound **119** showed significant results at three graded doses (25 mg/kg, 50 mg/kg, and 100 mg/kg p.o.) [142]. Ruiz et al., prepared (Z)-3-(2-(argiodiazenyl)phenyl)-*N*-((4-(5-(p-tolyl)-3-(trifluoromethyl)-1*H-*pyrazol-1yl)phenyl)sulfonyl)propanamide derivatives (**120**) and assessed them for inhibition of COX-2 compared to celecoxib [143]. Burguete et al., synthesized newer pyrazole derivatives and assessed them for anti-inflammatory potential, in which compound **121** showed significant anti-inflammatory potential [144] (Refer to Table 9 for the structure of the compound). Sandeep et al., prepared 3-substituted-1-aryl-5-phenyl-6-anilino-pyrazolo[3,4-d]pyrimidin-4-one derivatives and tested them for anti-inflammatory potential. Compound **122** revealed significant edema inhibition of 35–39 after 3 h of administering synthesized derivatives compared to diclofenac sodium and celecoxib (25 mg/kg). [145]. Hamdy et al., prepared pyrazolopyridine derivatives, among which compound **123** appeared to be a potent anti-inflammatory inhibitor [146]. Sharath et al., carried out the synthesis of newer indole-linked pyrazole derivatives and evaluated them for anti-inflammatory potential, in which compound **124** appeared as the most active derivative [147]. Thore et al., reported ethyl-5-amino-3-methylthio-1*H-*pyrazole-4-carboxylate derivatives and evaluated them for analgesic and anti-inflammatory properties via the writhing test. Compound **125** showed good analgesic and anti-inflammatory potential (25 mg/kg) with 0.9–1.12 ulcerogenic index in comparison to diclofenac sodium, which showed ulcerogenic index values of 3.10 [148]. Bandgar et al., prepared 1-methyl-5-(2,4,6-trimethoxyphenyl)-1*H-*pyrazole derivatives and assessed them for anti-inflammatory potential. Compound **126** appeared to be the most potent. [149]. Keche et al., prepared 1-acetyl-3-(3,4-dimethoxyphenyl)-5-(4-(3-(arylureido/arylthioureido/arylsulfonamid o)phenyl)-4,5-dihydropyrazole derivatives, in which compound **127** reflected the highest potential inhibition of inflammation in comparison to diclofenac sodium [150] (Refer to Table 9 for the structure of the compound). Selvam et al., prepared 1-(4-substitutedphenyl)-3-phenyl-1*H-*pyrazole-4-carbaldehyde derivatives and evaluated them for anti-inflammatory potential, among which **128** yielded excellent results [151]. El-Sayed et al., prepared pyrazole derivatives, among which compound **129** showed optimum COX-2 inhibition (IC_50_ = 0.26 µM and SI = 192.3) compared to celecoxib (IC_50_ = 0.28 µM and SI = 178.57) [127] (Refer to Table 9 for the structure of the compound).

Nagarapu et al., reported 3-phenyl-*N*-[3-(4-phenylpiperazin-1yl)propyl]-1*H-*pyrazole-5-carboxamide derivatives, among which compound **130** appeared to be the best potential anti-inflammatory derivative [152] (Refer to Table 10 for the structure of the compound). Bandgar et al., developed 3,5-diaryl pyrazole derivatives, in which compound **131** revealed excellent inhibition of inflammation [138] (Refer to Table 10 for the structure of the compound). Barsoum et al., synthesized bis(3-aryl-4,5-dihydro-1*H-*pyrazole-1-carboxamide) derivatives and evaluated them for inhibition of inflammation, in which compound **132** appeared as the best potential derivative compared to indomethacin [141] (Refer to Table 10 for the structure of the compound). Burguete et al., reported newer pyrazole derivatives and assessed them for anti-inflammatory activity. Compound **133** displayed good inhibition of inflammation [144]. Bekhit et al., prepared 1*H-*pyrazole scaffolds and assessed them for anti-inflammatory and analgesic potential. Many derivatives displayed inhibition of inflammation with reduced ulcerogenic potential. Compound **134** showed significant and selective COX-2 inhibition in comparison to indomethacin [153] (Refer to Table 10 for the structure of the compound). Amir et al., reported anti-inflammatory potential of 3,5-dimethylpyrazole derivatives and 3-methylpyrazole-5-one derivatives. Compounds **135a** and **135b** showed significant anti-inflammatory, analgesic, lipid peroxidation, and ulcerogenic activities. [154] (Refer to Table 10 for the structure of the compound). Furthermore, 4-[((1-(2-hydroxyethyl)-3,5-dimethylpyrazole-4-yl)azo)phenyl]-4-(2-phenethyl)-2,4-dihydro-3*H-*1,2,4-triazole-3-thione derivatives were prepared and assessed for analgesic effect by Oruc et al using morphine as the standard drug. Compound **136** yielded the most potent results [155]. Bekhit et al., prepared derivatives containing the pyrazolyl benzene sulfonamide moiety and assessed them for anti-inflammatory activity. Compound **137** displayed significant results in comparison to indomethacin [156] (Refer to Table 10 for the structure of the compound). Chowdhury et al., prepared newer 4-[2-(4-methyl(amino)sulfonylphenyl)-trifluoromethyl-2*H-*pyrazol-3-yl]-1,2,3,6-tetrahydropyri -dine derivatives and inspected them for inhibition of inflammation. The outcomes showed that compound **138** exhibited the maximum inhibition of inflammation in comparison to celecoxib and aspirin [157] (Refer to Table 10 for the structure of the compound). Abdel-Hafez et al., prepared pyrazole-NO hybrids and assessed them for potential inhibition of inflammation. Compound **139** appeared potent in comparison to indomethacin [139]. El-Din et al., developed newer pyrazole derivatives and assessed them for anti-inflammatory potential [158]. Compound **140** presented the best potential outcomes with minimal ulcerogenicity without affecting renal function. Bekhit et al., reported 4-thiazolylpyrazolyl derivatives and tested them for inhibition of inflammation, among which compound **141** displayed significant inhibition and was considered as a potential COX-2 inhibitor [159]. Youssef et al., reported dipyrazoleethandiamides and cyano-1-phenyl-1*H-*pyrazol-5-yl]acetamide derivatives, of which compound **142** showed significant anti-inflammatory activities [160] (Refer to Table 10 for the structure of the compound). A novel class of 3-phenyl-*N*-[3-(4-phenylpiperazine-1-yl)propyl]-1*H-*pyrazole-5-carboxamide derivatives were prepared by Nagarapu et al., in which compound **143** yielded excellent results [152]. Shrivastava et al., prepared pyrazole containing 1,5-diaryl derivatives and evaluated them for anti-inflammatory potential. Compound **144** demonstrated the significant inhibition of inflammation in comparison to control and standard [161]. *N*-(substitutedaryl/alkylcarbamothioyl)-4-[5-(4-methylphenyl)-3-(trifluoromethyl)-1*H-*pyrazol-1-yl]benzenesulfonamide compounds were prepared and investigated by Kucukguzel et al., for anti-inflammatory potential. The outcomes suggested that compound **145** revealed potential activity without causing any cell damage to the liver, kidney, and brain in comparison to celecoxib [162]. Calis et al., reported pyrazole-3(5)-carboxylic acid derivatives and tested them for anti-inflammatory potential. Compound **146** was designated with the highest anti-inflammatory potential compared to indomethacin and aspirin as standard drugs [163] (Refer to Table 10 for the structure of the compound).

Chavan et al., described novel pyrazole amalgamated flavones and assessed them for inhibition of inflammation. Compound **147**, the 1*H-*pyrazole derivative with the 2,3,6-trimethoxyphenyl group, showed the best potency [164] (Refer to Table 11 for the structure of the compound). Baytas et al., prepared (E)-3-[3-(pyridin-4-yl)-1-phenyl-1*H-*pyrazole-4-yl]acrylamides and tested them for anti-platelet and anti-inflammatory activity [165]. Compound **148** revealed significant activity. Ragab et al., reported 1,3,4-trisubstitutedpyrazole containing derivatives and evaluated them for their anti-inflammatory and analgesic potential and ulcerogenicity [166] (Refer to Table 11 for the structure of the compound). Compounds **149**, **150,** and **151** showed good inhibition of inflammation and analgesic potential. They showed less selectivity for COX (I/II) but displayed good tolerance to GIT in comparison to phenylbutazone. Derivatives of pyrazole carboxamides bearing naproxen were developed by El-Sehemi et al., and assessed for anti-inflammatory potential. Compound **152** appeared to be the best potential derivative [167] (Refer to Table 11 for the structure of the compound). Hassan et al., prepared celecoxib analogues with a benzofuran ring and assessed them for COX (I/II) inhibitory bioassay. Among all these compounds, compound **153** displayed noteworthy inhibition of inflammation. The outcome exposed the vital role of derivatives containing C-3-pyridine-3-yl to exhibit better anti-inflammatory potential [6]. Kumar et al., reported pyrazolyl pyrazoline-containing derivatives and tested them for anti-inflammatory potential. Compound **154** appeared to have the most potential as an anti-inflammatory compound [168]. Mohammed et al., prepared hydrazone linked pyrazole derivatives. These compounds were evaluated for anti-inflammatory activity, blocking PGE2 production in blood serum of adult wistar rats, COX-1/2 inhibition, and ulcerogenicity. Compound **155** displayed significant anti-inflammatory efficacy compared to diclofenac, with no or minimal ulcerogenicity observed in comparison with indomethacin as a standard drug. The pyrazole derivatives demonstrated SI of 11.1 for COX (I/II) inhibition [169] (Refer to Table 11 for the structure of the compound). Pyrazole derivatives were prepared by Tewari et al., and investigated for anti-inflammatory potential and COX-2 inhibition [170]. Among these, compound **156** with IC_50_ = 0.44 µM and **157** with IC_50_ = 0.51 µM showed the most significant results compared to celecoxib with IC_50_ = 0.002 µM for COX-2 selectivity and paw volume of 1.46 and 1.11 compared to the reference drug nimesulide, which showed a paw volume of 1.26. Alegaon et al., prepared 1,3,4-trisubstituted pyrazole derivatives and assessed them for anti-inflammatory potential [171] (Refer to Table 11 for the structure of the compound). Compound **158** presented 64.6% inhibition of inflammation in comparison to diclofenac with 86.72% inhibition. Bansal et al. [172] prepared 1,3,4-oxadiazole-linked diarylpyrazole derivatives, in which compound **159** exhibited potential COX-2 inhibition with maximum selectivity compared to aspirin. Wang et al., synthesized pyrazole-linked benzamide derivatives and evaluated them for inverse agonistic activity on retinoic acid receptor Y. Compound **160** was identified as a potential lead molecule [173]. Newer pyrazole derivatives were prepared and evaluated for anti-inflammatory activity by Li et al.; among these, compound **161** exhibited better potency in comparison to indomethacin and ibuprofen [174] (Refer to Table 11 for the structure of the compound).

Wang et al., synthesized benzamide-containing pyrazole derivatives and investigated them for anti-inflammatory potential. Compound **162** showed potential anti-inflammatory results [173]. El-Fekyet al., carried out a series of quinoline-integrated pyrazole scaffolds and assessed them for anti-inflammatory property. Compound **163** revealed the maximum inhibition and more binding affinity for the COX-2 binding position [175] (Refer to Table 12 for the structure of the compound). Newer pyrazole-containing heterocycles were prepared by Kendre et al., and tested for analgesic potential. Scaffold **164** displayed good analgesic activity [176]. Kumar et al., synthesized novel pyrazole derivatives and identified anti-inflammatory activity. Compound **165** appeared to be the best potential inhibitor of inflammation [177]. Pelcman et al., prepared pyrazole carboxamide derivatives and screened them for inhibitory activity against 15-lipoxygenase. Scaffold **166** appeared to show significant anti-inflammatory potential with good % inhibition and analgesic activity [178] (Refer to Table 12 for the structure of the compound). Compound **167** was identified as the most potent and selective inhibitor of COX-2 with IC_50_ = 1.33 µM. Newer pyrazole-linked analogues were prepared and tested for analgesic potential by Abdellatif et al.; among these synthesized compounds, compound **168** demonstrated the highest anti-inflammatory action post carrageenan after 3 h of inflammation initiation (89% inhibition) as compared to the standard drug celecoxib (80% inhibition) [179] (Refer to Table 12 for the structure of the compound). Abdel-Aziz et al., prepared newer pyrazole derivatives, of which compound **169** exhibited remarkable anti-inflammatory potential with ED_50_ = 68 ± 2.2 mg/kg and 51 ± 0.7 mg/kg [180]. Doma et al., reported novel pyrazole analogues and evaluated them for anti-inflammatory activity using 0.1% carrageenan and injecting it into the subplantar region of the rat’s right paw. Derivative **170** was found to be the most significant member of the class [181]. Newer benzophenones were reported by Bandgar et al., and assessed for analgesic potential. Compound **171** showed significant inhibition of inflammation and analgesic activity [149] (Refer to Table 12 for the structure of the compound). Jadhav et al., prepared pyrazole derivatives and tested them for inhibition of inflammation. Compound **172** demonstrated the highest anti-inflammatory results when compared to diclofenac [182]. Vijesh et al., synthesized newer pyrazole-bearing 1,2,4-triazole and benzoxazole derivatives and assessed their anti-inflammatory potential. Compound **173,** containing dichlorothiophene and triazole, was confirmed to have good analgesic and anti-inflammatory potential [183]. Aggarwal et al., reported many pyrazole-containing derivatives and investigated their analgesic efficacy. Compound **174** revealed noteworthy inhibition of inflammation (62–76%) in comparison to indomethacin (78%) [184] (Refer to Table 12 for the structure of the compound). Among the leads established by Yewale et al., compound **175** appeared as the most potent molecule with superior anti-inflammatory potential compared to diclofenac sodium and equivalent results against the standard drug celecoxib (dose 25 mg/kg) [145] (Refer to Table 12 for the structure of the compound).

Kamble et al., reported thiazole-bearing pyrazole derivatives and assessed them for anti-inflammatory potential. Compound **176** yielded the most effective results [185] (Refer to Table 13 for the structure of the compound). Chougala et al., prepared coumarin-bearing pyrano[2,3-c]pyrazole derivatives and screened them via an anti-inflammatory bioassay against protein denaturation and HRBC membrane stabilization model. Compound **177** exhibited the most potent anti-inflammatory results [186]. Somakala et al., reported newer pyrazolylurea derivatives and evaluated them for their p38α MAPK (mitogen-activated protein kinase) inhibition. Among these compounds, compound **178** showed p38α MAPK inhibition with IC_50_ values ranging 0.069 ± 0.07 mmol/L against SB203580 (IC_50_ = 0.043 ± 3.62 mmol/L). Compound **178** also indicated promising in vivo anti-inflammatory potency (80.93% inhibition) compared to diclofenac sodium (81.62% inhibition) [187]. Maddila et al., prepared 1,3,4-thiadiazole linked pyrazole-3-carboxamide derivatives and evaluated them for anti-inflammatory potential. Compound **179** showed significant inhibition of inflammation (77.27% and 81.00% at 3 h and 5 h) in comparison to indomethacin (74.82% and 80.32% at 3 h and 5 h) [188]. Abdellatif et al., synthesized 1,3,5-triaryl-4,5-dihydro-1*H-*pyrazole derivatives and assessed their inhibition of COX enzymes, anti-inflammatory potential, analgesic potential and ulcerogenicity. Compound **180** displayed significant anti-inflammatory potential with ED_50_ = 53.99 µmol/kg in comparison to celecoxib with ED_50_ = 82.15 µmol/kg, with lessen ulcer index (1.20 for compound **180** and 2.90 for celecoxib) [189]. Elshemya et al., prepared triazine-linked pyrazole derivatives and assessed them for inhibition of COX-2. Compound **181** displayed the best potential results with IC_50_ = 0.74 μM compared to celecoxib with IC_50_ = 0.78 μM [190] (Refer to Table 13 for the structure of the compound). Levent et al., reported diarylpyrazole-bearing carboxamide derivatives as newer antiplatelet drugs that interfere with platelet aggregation and inflammation (in cardiovascular diseases). Compound **182** revealed potential results with IC_50_ = 5.7–8.3 nM [191]. Unlu et al., prepared novel regioisomeric 1-(3-pyridazinyl)-5-arylpyrazole derivatives and evaluated them for anti-inflammatory and analgesic potential. Compound **183** displayed inhibitory potency for COX-I/II and can be a lead for future investigations [192] (Refer to Table 13 for the structure of the compound). A novel class of 1-phenylpyrazolo [3,4-d]pyrimidine derivatives were prepared by Bakr et al., and assessed for inhibition of COX, anti-inflammatory activity, and ulcerogenicity. Compound **184** displayed a higher edema inhibition of 34–68%; however, the in vivo active compound showed flexible ulceration (ulcer index = 0.33–4.0) compared to celecoxib (ulcer index = 0.33) [193]. Chandak et al., synthesized new 2-amino-substituted 4-coumarinylthiazoles-bearing benzenesulphonamide moieties and determined their anti-inflammatory potential. Compound **185** revealed promising anti-inflammatory activity (0.11 ± 0.16% IA) equivalent to the standard drug indomethacin (0.17 ± 0.03% IA) [194]. Faour et al., synthesized di-pyrazole comprising *N*-phenylpyrazole nucleus containing polysubstituted pyrazole moieties through different amide linkages and investigated them for inhibition of COX-2, LPS induced iNOS, and anti-inflammatory and analgesic activities. Compound **186** inhibited COX-2 with IC_50_ = 11.2 µM with 78% inhibition of inflammation, compared to 53% inhibition by diclofenac, and 54% protection against pain compared to 52% protection by diclofenac [195]. Alam et al., prepared hybrid pyrazole analogues, among which compound **187** exhibited anti-inflammatory potential with 80.63% inhibition after 4 h in comparison to ibuprofen (with 81.32% inhibition after 4 h and inhibition against COX-1/2 and TNF-α). The same compound showed high COX-2 inhibition, with half maximal inhibitory concentration of 1.79 μM compared to celecoxib [196] (Refer to Table 13 for the structure of the compound).

Compound **188** (AD732) was designed as a pyrazole derivative with anti-inflammatory and analgesic properties. Compound **188** (AD732) showed a strong inhibitory effect against COX-2 (IC_50_ = 0.57 ± 0.04 µM) rather than COX-1. Compound **188** (AD732) was compared to the standard celecoxib (IC_50_ = 0.26 ± 0.32 μM target on COX-1, IC_50_ = 2.62 ± 0.02 μM target on COX-2) and indomethacin medications (IC_50_ = >100.00 μM target on COX-1, IC_50_ = <0.30 μM target on COX-2) for anti-inflammatory action. In comparison to celecoxib, the COX-2 inhibitory activity of compound **188** (AD732) was less effective in vitro, suggesting that it may have less cardiovascular toxic effects. To summarize, compound **188** (AD732) appears to be a better and more efficient compound with intriguing promise for pain and inflammatory control. When treated rats were assessed 24 h following a single higher dose treatment, neither celecoxib nor compound **188** (AD732) caused stomach ulcers. The superior gastrointestinal safety of AD732 is based on its favorable inhibitory efficacy against COX-2 over COX-1 [197] (Refer to Table 14 for the structure of the compound). A novel pyrazole derivative was prepared and evaluated for inhibition of COX-1/2. The pyrazole ring’s bridging amide N generates a reasonably stable H-bond with Ser 530. Compounds **189**(**a**), **189**(**b**), **189**(**c**), and **189**(**d**) showed high activity and selectivity against COX-2 (IC_50_ = 39.43, 61.24, 38.73, and 39.14 nM). All compounds reported selectivity indices of 22.21, 14.35, 17.47, and 13.10, for **189**(**a**), **189**(**b**), **189**(**c**), and **189**(**d**), correspondingly. With IC_50_ = 34.21 nM, compound **189a** replaced with acetamide morpholine appeared to be a potential COX-2 inhibitor. The IC_50_ values for compounds **189c** and **189d** substituted with fluoro and methoxy groups were 38.73 and 39.14 nM, correspondingly. In vivo, these compounds were superior to or equivalent to celecoxib as anti-inflammatory drugs. In silico, the same molecules exhibited comparable interactions to SC-588. Surprisingly, these drugs preferentially reduced the synthesis of the triggered microsomal PGE2 synthase [198] (Refer to Table 14 for the structure of the compound). Pyrazole and triazole compounds were synthesized, docking studies were performed, and their biological activity towards selective COX-2 inhibitors was investigated. Compound **190a,** when substituted with sulfonamide on one *N*-aromatic ring, exhibited the highest inhibitory action against COX-2 with IC_50_ = 0.017 ± 0.001 μM and COX-1 with IC_50_ = 0.263 ± 0.016 μM. Compound **190b**, which was replaced with nitro on the *N*-aromatic ring, inhibited COX-1 more effectively, with COX-1 (IC_50_ = 0.012 ± 0.001 μM). All compounds were compared with the other existing drug celecoxib, which demonstrated anti-inflammatory action towards COX-1 and COX-2 with IC_50_ = 1.479 ± 0.089 μM and 0.004 ± 0.000 μM, correspondingly. Because of its lower volume and far more optimal arrangement of the NO_2_ in same area, **190b** was the most effective pyrazole-containing COX-1 inhibitor. The presence of a spacer in the inhibitor structure is important because it places the aromatic ring in a favorable location for hydrophobic interaction in COX-2. There are 2 aryl groups on the heterocyclic cores (pyrazole or triazole) that are not the same as those on most COX-2 inhibitors [199] (Refer to Table 14 for the structure of the compound).

Novel pyrazolo[3,4-d]pyridazinone-containing derivatives were developed and assessed against the alterations of discoidin domain receptor1 (DDR1) as anti-inflammatory agents. Compound **191** demonstrated strong inhibition of discoidin domain receptor1 (DDR1) with an IC_50_ value of 10.6 ± 1.9 nM and significant selectivity against 430 kinases. In a dextran sulphate sodium prompted animal colitis model, compound **191** potently reduced the production of pro-inflammatory cytokines and DDR1 auto phosphorylation in cells. The removal or relocation of the trifluoromethyl group in Compound **191** resulted in a significant loss of efficacy against the DDR1 outcome. The activity was significantly reduced when trifluoromethyl-substituted phenyl was replaced with n-butyl or cyclohexyl. To retain robust DDR1 inhibitory activity levels ranging from 27.4 to 60.4 nM, the acetylaminophenyl group at the R1 position might be substituted with phenyl or substituted phenyl. In mice’s bone-marrow-derived macrophages and human THP-11 generated macrophages, compound **191** effectively reduced LPS-induced production of these pro-inflammatory cytokines at 10 µM. Compound **191** suppressed basal autophosphorylation of discoidin domain receptor1 (DDR1) with an EC_50_ of 34.4 nM, which was more powerful than the positive control DDR1-IN-1, which had an EC_50_ of 114.5 nM [200].

## 4. Authors Perspective

The main goal of this revised study is to highlight the anticancer and anti-inflammatory profiles of the pyrazole molecule. Cancer is an uncontrollable abnormal cell proliferation that is a severe life-threatening disorder worldwide. Pharmaceutical firms are now investing heavily in developing and enhancing viable and safe anticancer treatments with minimal side effects. Pyrazole derivatives exhibit anti-inflammatory properties and have been shown to target several cancer cell lines, such as COX (I/II). The description includes a library of powerful compounds against cancer cell lines and inflammation. Among them, certain effective compounds and their SAR structures are strongly associated with biological activity and predicted some additional derivatives that approach structural alterations. We addressed the anticancer therapeutic action of compounds; all derivatives showed strong inhibitory activity, but certain powerful compounds demonstrated good inhibitory activity against cancer cell lines.

### 4.1. Pyrazole Biomolecules as Cancer Therapeutics

**Figure 4:**
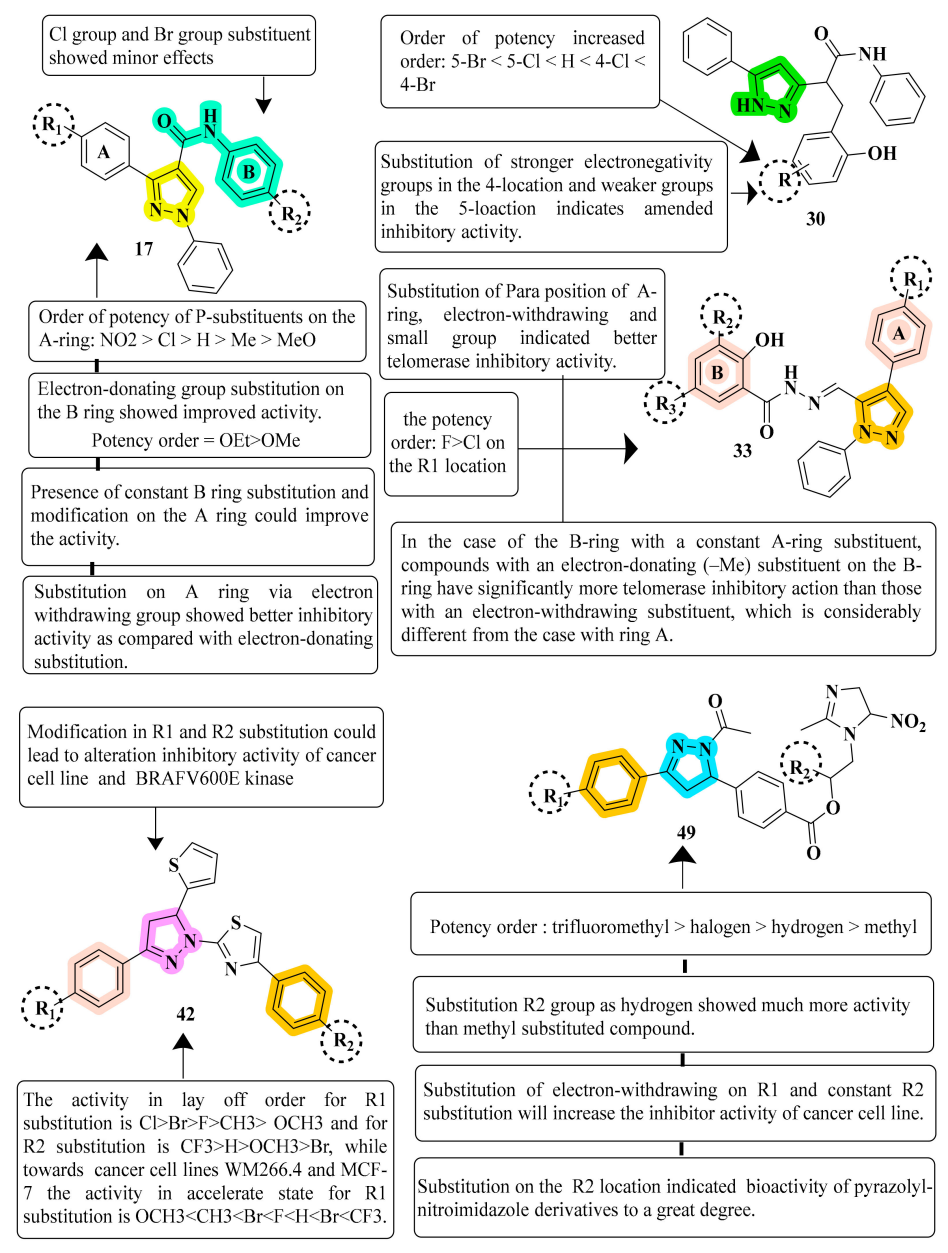
Structure and SAR of potent anticancer activity compounds **17**, **30**, **33**, **42**, and **49**.

Compound **21** exhibited the most strong biological activity against HCT116, MCF-7, and Aurora-A kinase inhibitory activity cell lines, with IC_50_ values of 0.39 ± 0.06 µM, 0.46 ± 0.04 µM, and 0.16 ± 0.03 µM, which were equivalent to the positive control. The scaffolds of the known inhibitors of Aurora-A kinase can be categorized into four main groups, A–D. The scaffold in A contains 1,4,5,6-tetrahydropyrrolo[3,4-c]pyrazole; the scaffold in B contains pyrrolo[2,3-b]pyrimidine; the scaffold in C contains quinoline; and the scaffold in D contains 2-anilino-diaminopyrimidine. Benzamide is a significant pharmacophore of natural compounds as well as a synthetic precursor for a variety of medications. Benzamide has been shown to have several pharmacological effects, including anti-inflammatory, anticancer, and anti-fungal properties. To create a more potent reaction with the Aurora-A kinase domains, a benzamide moiety was transferred to the pyrazole moiety to create the N phenyl-1*H-*pyrazole-4-carboxamide template. The highest activity was found for 21 compounds with a para-NO2 group on the A-rings and a para-OEt group on the B-rings [44] (refer to Figure 4 for Structure and SAR). In a separate investigation, compound **30** demonstrated a significant inhibitory effect against WM266.4 and A375 with IC_50_ values ranging from 1.50 to 1.32 µM. It makes use of a common scaffold comprised of a terminal aromatic group (phenyl or pyrazole) that occupies the allosteric pocket generated by the displacement of the DFG loop and an amide linker that links an aryl group in the hydrophobic pocket to a hinge-binding heterocycle. The use of an amide bond would allow for the quick and efficient screening of several candidate hinge-binding groups. The original molecule was modified to see how the substituents affected the BRAFV600E inhibitory effects. Urea derivatives have higher inhibitory effects than Schiff base compounds and related intermediate amines. Changes in substituents on the benzene rings had a minor impact on the activity of the compounds. In contrast, differences in the skeleton had a greater impact, which may explain why substituents on the benzene rings had a minor impact on the ability for molecular bonding between BRAFV600E and the compounds. According to the findings, the pyrazolyl core and the phenyl urea motif are critical in the inhibition of BRAFV600E [51] (refer to Figure 4 for Structure and SAR).

Compounds with the OH and Me groups on the B-ring, compound **33**, resulted in notable activity. Compound **33** displayed the most powerful effect, inhibiting the growth of MCF-7 and B16-F10 cells with IC50 values of 0.57 ± 0.03 and 0.49 ± 0.07 µM, respectively, and inhibiting the activity of telomerase with an IC_50_ of 1.9 ± 0.43 µM. More than one way to achieve powerful coordination may be achieved by using hydrazone derivatives and, specifically, acyl hydrazones. Acylhydrazone derivatives have the potential to form complexes with metal ions, such as Fe^3+^ and Ni^2+^, resulting in the inhibition of a physiologic reaction catalyzed by ions or the promotion of the absorption, transportation, distribution, and metabolism of drugs in the body, respectively. The F group outperformed the Cl group in antiproliferative action on the A ring, which might be attributable to the fluorine substituent’s greater lipophilicity and metabolic stability. The presence of fluorine often increases the solubility of lipids, which increases the rates of absorption and transport of drugs. Compound **33**, based on its data structure, may be useful in designing and manufacturing stronger telomerase inhibitors [54] (refer to Figure 4 for Structure and SAR).

A series of novel BRAF-targeting pyrazole-based derivatives developed recently may prove useful against cancer. Some of them proved to have potential antitumor activity, particularly compound **42**, which had an IC_50_ value of 0.12 µM against the cell line WM266.4 and 0.16 µM against the cell line MCF-7 and which also showed the most potential antiproliferative activity of apoptosis by flow cytometry experiments. Common scaffolds consist of a terminal aromatic group (phenyl or pyrazole) that fills the allosteric pocket. Thriophene and thiazole groups were demonstrated to have antibacterial, anti-inflammatory, and antiviral properties according to generalized research. The relevant compounds were formed by displacing the DFG loop and an amide linker and linking an aryl group in the hydrophobic pocket to a hinge-binding heterocycle. The introduction of an amide bond would enable the rapid and effective screening of different hinge-binding groups. Changes in substituents on the benzene thiazole rings had only a small effect on the compounds’ activity. As a consequence, compound **42** and the other pyrazole derivatives with effective thiazole and thiophene pharmacophores are promising candidates for further research as anticancer medicines [62](refer to Figure 4 for Structure and SAR).As shown in the figure below, compound **49** acts as a potent inhibitor of HER-2/EGFR, possessing a half-life of 0.26 m/0.51 m compared to the positive controls erlotinib (IC_50_ = 0.41 µM for HER-2 and IC_50_ = 0.20 µM for EGFR) and lapatinib (IC_50_ = 0.54 µM for HER-2 and IC_50_ = 0.28 µM for EGFR). On the mechanistic level, nitroimidazole derivatives drew much attention because of their ability to enter and accumulate in tumor areas. To boost the antiproliferation activity of pyrazolyl-nitroimidazole derivatives against Hela or HepG2 cell lines, electron-withdrawing groups were shown to be preferred to electron-donating groups. This indicates that the synthesized chemicals’ significant inhibitory effects on cell growth were directly connected to their kinase inhibitory actions [68] (refer to Figure 4 for Structure and SAR).

**Figure 5:**
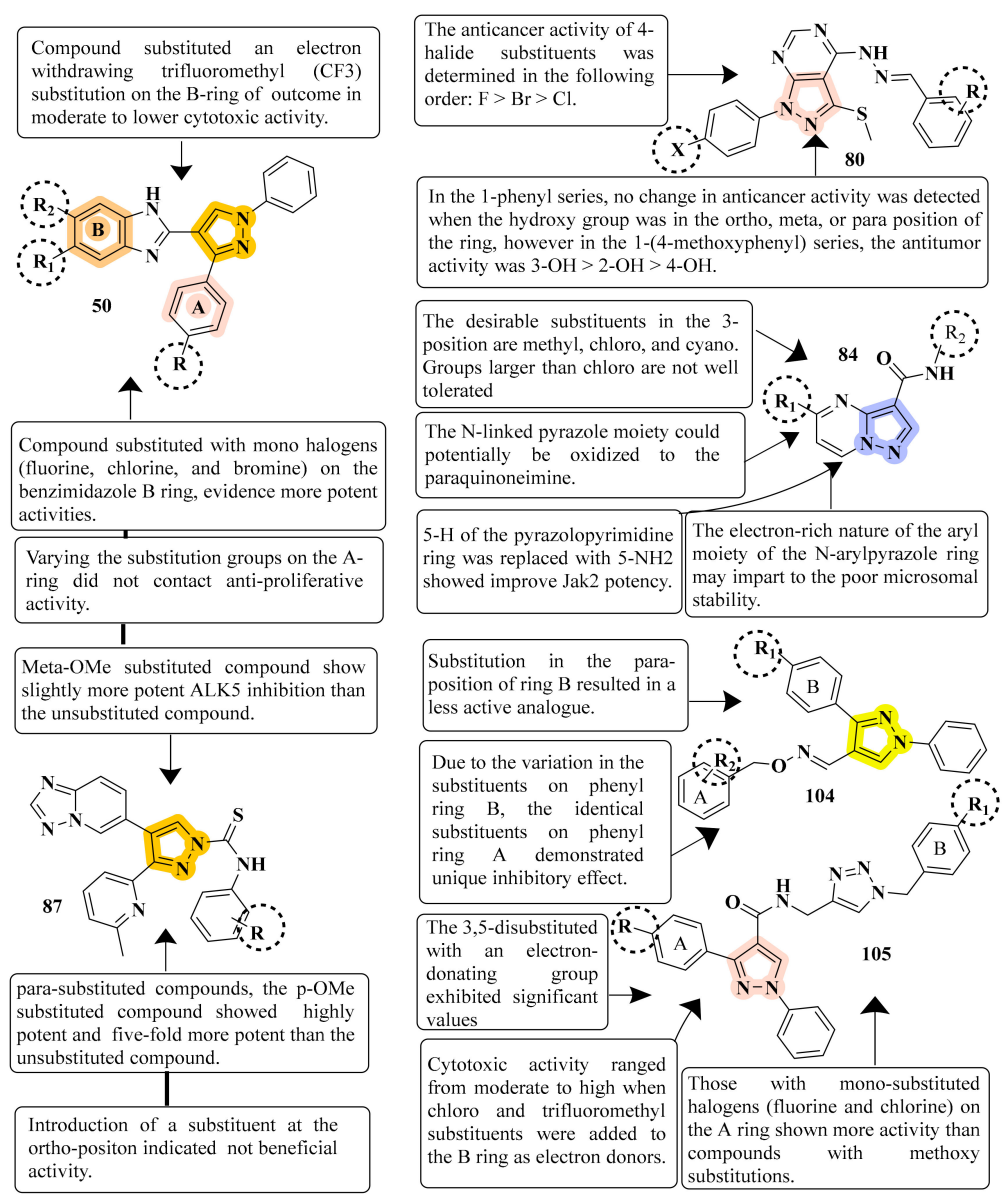
Structure and SAR of potent anticancer activity compounds **50**, **80**, **87**, **104**, and **105**.

These molecules are intriguing novel frameworks for the case of cancer therapies due to their simplicity of synthesis and extraordinary biological activity. Compound **50** inhibited MCF-7, A549, and HeLa cells significantly, with IC_50_ values ranging from 0.83–1.81 µM. Furthermore, the compounds induced G1 phase cell cycle arrest in MCF-7 cells by inhibiting cyclin D1 and CDK2. It was observed that structural alterations and modifications on the B ring of pyrazolobenzimidazole derivatives resulted in distinct cytotoxic effects by studying the difference in selectivity of the three cell lines to the compounds. As a result, pyrazole-benzimidazole hybrids may lower cell cycle regulators, including cyclin D1 and CDK2 in MCF-7 cells, causing cell cycle arrest and growth suppression [69] (refer to Figure 5 for Structure and SAR). Because the pyrazolo[3,4-d]pyrimidine nucleus is an isostere of the purine nucleus, it has an anticancer effect via serving as an ATP competitive inhibitor for several kinase enzymes. It has been reported that hydrazinyl derivatives have anticancer properties, particularly in breast cancer cell lines. There is evidence that a methyl sulphonyl ring at position 3 enhances the antitumor activity of pyrazolo[3,4-d]pyrimidine nucleic. This study showed that compound **6d** (X = H, R = 4-F) showed the highest IC_50_ at 7.5 nM. More research is needed to understand the precise mechanism of antitumor activity and investigate the SAR of various nucleus locations [94] (refer to Figure 5 for Structure and SAR). A high-throughput screening method identified the pyrazolo[1,5-a]pyrimidine scaffold as a potent Jak2 inhibitor. Increased polarity and the elimination or inhibition of metabolic hotspots such as N-methyl groups are two often used approaches for increasing stability against CYP-mediated metabolism. The H-bond accepting properties of oxygen atoms in heteroaromatic ring systems are weak; on the other hand, sp2 nitrogens of heteroaromatics tend to be strong H-bond acceptors. Compound **84** was found to be the most promising derivative, with an IC_50_ of 7.4 nM. Compound **84** offers a good combination of cell potency, oral exposure, and in vivo pSTAT5 knockdown, making it a feasible tool for initial Jak2-dependent efficacy studies. Any alteration at the para-position of the N-aryl group reduces the inhibitory potency of all Jak family members considerably. The pyrazolo[1,5-a]pyrimidine scaffold was shown to be a strong inhibitor of Jak2 in a high-throughput screening study [98] (refer to Figure 5 for Structure and SAR).

Several of the pyridyl-substituted five-membered heterocyclic rings were boosted in their inhibitory activity and selectivity by adding methylene, methylene amino, or amino methylene linkages between their core rings and their phenyl rings. The most active compound **87** inhibited ALK5 phosphorylation with a IC_50_ value of 0.57 nM and demonstrated 94 percent inhibition at 100 nM in a luciferase reporter test using HaCaT cells irreversibly transfected with a p3TP-Luc construct. There was a significant increase in both the inhibitory activity and selectivity of compound **87** against p38a MAP kinase upon the incorporation of the [1,2,4]triazolo[1,5-a]pyridin-6-yl and phenycarbothioamide moiety at positions 4 and 1 of the pyrazole ring, respectively [101] (refer to Figure 5 for Structure and SAR).This work demonstrated the strong immunosuppressive actions of a variety of new pyrazole derivatives having an O-benzyl oxime moiety. Compound **104** had the greatest inhibitory efficacy (IC_50_ = 1.18 µM for lymph node cells and IC_50_ = 0.28 µM for PI3K). In particular, a variety of synthesized pyrazole oxime ether analogs were shown to have significant immunosuppressive effects in the low micromolar range. A study of the A-ring para substituents revealed that an electron-withdrawing group had increased immunosuppressive action, and the potency order is F > Cl > OMe > H > Me. The molecules with a potent inhibition effect towards IL-6 produced in ConA-stimulated murine lymph node cells were investigated [117] (refer to Figure 5 for Structure and SAR). The synthesis of N-((1-benzyl-1*H-*1,2,3-triazol-4-yl)methyl)-1,3-diphenyl-1*H-*pyrazole-4-carboxamides was reported well as their biological evaluation. Nocodazole (8.01–0.95 µM) exhibited significant inhibition against MIAPaCa-2, MCF-7, and HeLa cell lines with compound **105** (GI_50_) in the range of 0.13–0.7 µM (GI_50_). Using the pyrazole moiety with two aryl ring systems, A and B, this study investigated the compounds with electron-withdrawing and electron-donating groups, i.e., chloro, fluoro, and fluoro trifluoromethyl groups on the ring systems (A and B). This compound **105** resulted in cell cycle arrest in MCF-7 cells and downregulation of CDK1 expression in MCF-7 cells when it was treated with it. As a result, they can be regarded as promising lead molecules for creating more effective anticancer medicines against breast cancer cells [118] (refer to Figure 5 for Structure and SAR).

**Figure 6:**
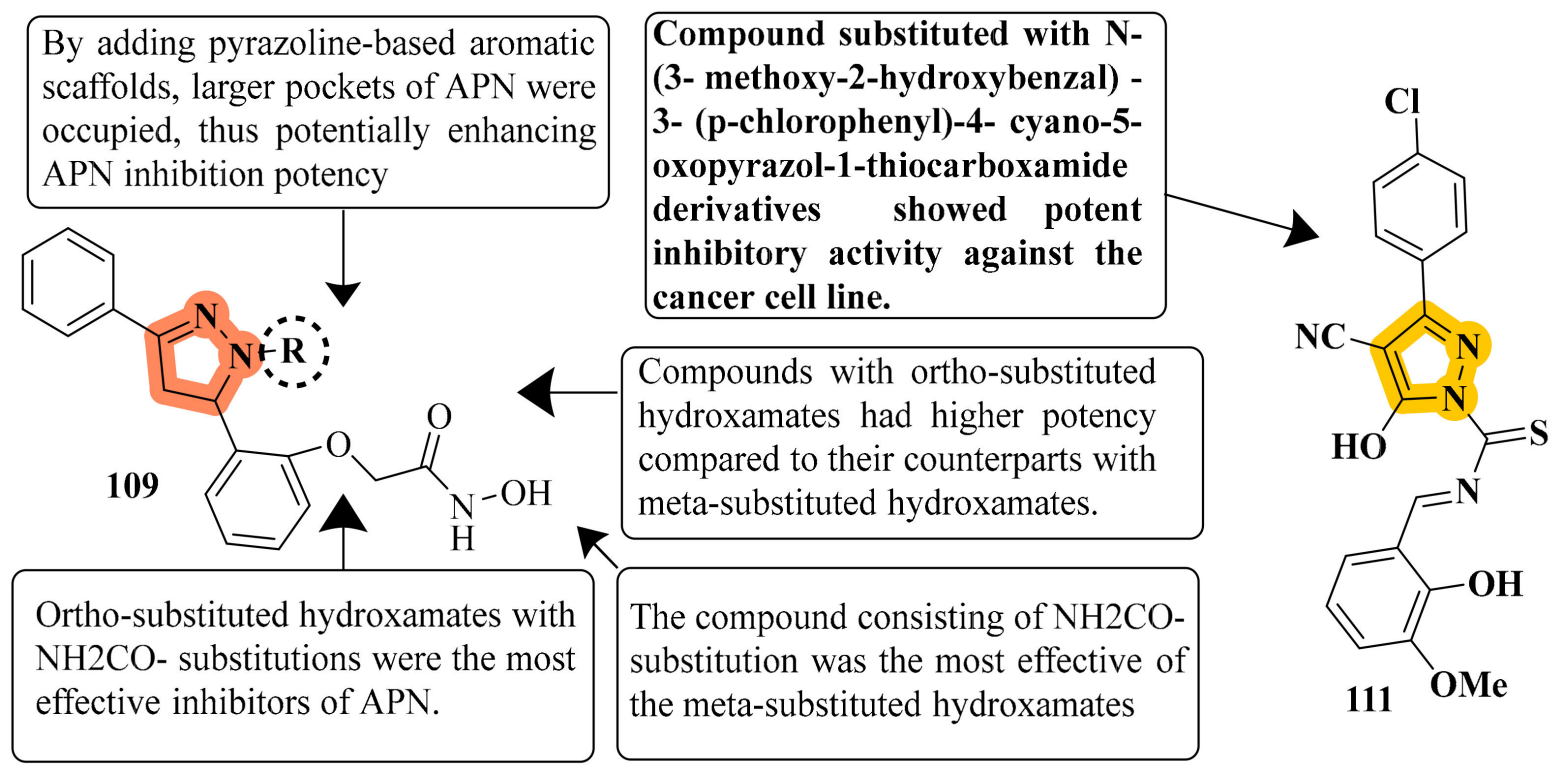
Structure and SAR of potent anticancer activity compounds **109** and **111**.

APN inhibition was best with compound **109**, which had an IC_50_ value of 0.16 ± 0.02 μM, a value over one order of magnitude less than that of bestatin (IC_50_ = 9.4 ± 0.5 μM). Depending on where R was substituted on the pyrazoline ring, these compounds could have significantly different APN inhibitory potencies. B series compounds have higher inhibitory activity when compared to APN equivalents. As determined by the structure–activity relationships (SARs) of A series compounds, only ortho- or meta-substituted hydroxamates can be synthesized from B series compounds, while para-substituted hydroxamates are inactive. Compound **109** might be a starting point for developing more powerful APN inhibitors. To test human APN against the ES-2 and PLC/PRF/5 cell surfaces, compounds with significant porcine kidney APN inhibitory properties were selected. APN is abundantly expressed on both cell lines’ membranes [122] (refer to Figure 6 for Structure and SAR). The strongest inhibitory effect was achieved by compound **111** with N-(3-methoxy-2-hydroxybenzal)-3-substituted(p-chlorophenyl)-4-cyano-5-oxopyrazol-1-thiocarboxamide against HL-60, with an IC_50_ of 1.35 μM compared to Dox’s IC_50_ of 2.02 μM. Compound **111** improved the G2/M phase from 11.05 percent to 39.22 percent compared to the untreated control. The highest intense cytotoxic action was demonstrated by compound **111**. In a study that included activity assays of apoptosis detection and Topoisomerase II inhibition, compound **111** was found to be an effective inhibitor of Topo II [124] (refer to Figure 6 for Structure and SAR).

### 4.2. Pyrazole Biomolecules as Inflammation Therapeutics

**Figure 7:**
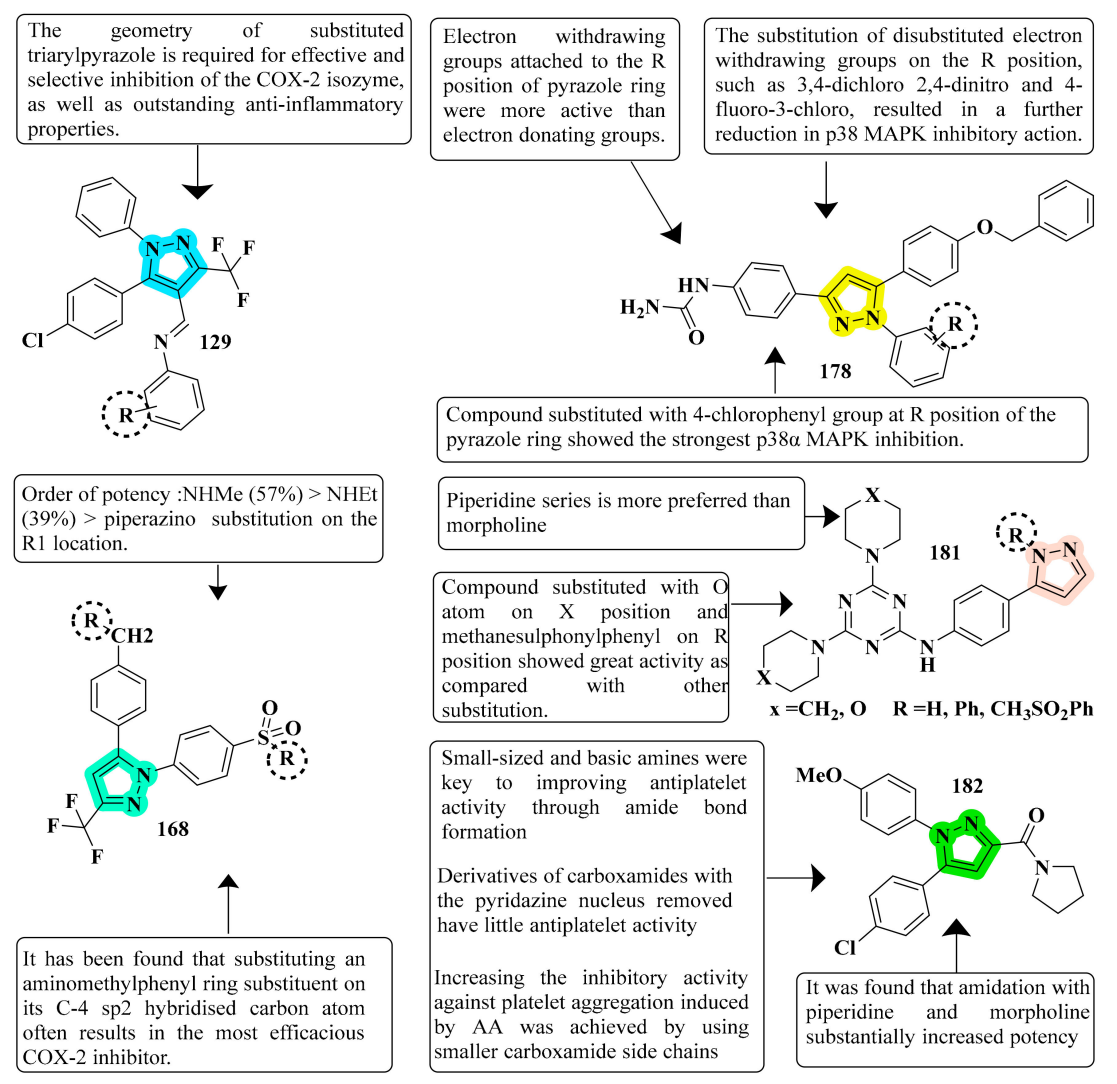
Structure and SAR of potent anti-inflammatory activity compounds **129**, **168**, **178**, **181**, and **182**.

The efficacy of new pyrazole and pyrazoline compounds to inhibit ovine COX-1 and COX-2 isozymes was assessed utilizing an in vitro cyclooxygenase (COX) inhibition test. Among the molecules examined, **129** exhibited excellent COX-2 inhibitory efficacy (IC_50_ = 0.26 µM) and selectivity (SI) = >192.3, which is equivalent to the reference medication celecoxib (IC_50_ = 0.28 µM and selectivity index = 178.57). Compound **129** is overly lipophilic due to the presence of trifluoromethyl fragments; therefore, it should serve as a lead molecule for further optimization of certain biological properties and ADME characteristics [127] (refer to Figure 7 for Structure and SAR). Novel 1-(4-methane(amino)sulfonylphenyl) derivatives 5th (4-substituted-aminomethyl-phenyl)-3-trifluoromethyl-1*H-*pyrazoles were developed, synthesized, and tested for biological activity. Compound **168**, derived from the library, exhibited significant anti-inflammatory action. Compared with the standard celecoxib drug (80% inhibition), compound **168** demonstrated the greatest anti-inflammatory effect post carrageenan, 3 h after inflammation onset (89% inhibition) [179]. A total of fifteen pyrazolyl urea derivatives were produced and tested for their ability to inhibit p38 MAPK and act as antioxidants. Among the tested compounds, derivative 178 was discovered to be the most powerful, and its binding mechanism inside the p38 MAPK was also documented. Compound **178**, which has a 4- chloro group and has significant p38 MAPK activity, also had the greatest anti-inflammatory efficacy (80.93 percent inhibition). The substitution of 2-chloro and 3-chloro groups for the 4-chloro groups resulted in a modest reduction in inactivity (76.19 percent and 78.06 percent inhibition, respectively). In both the in vitro and in vivo models, the monosubstituted electron-withdrawing group in the phenyl ring linked to the pyrazole nucleus showed strong anti-inflammatory efficacy. Compound **178** was found to be a strong anti-inflammatory agent, equivalent to the standard reference medication diclofenac sodium. In addition, compared to conventional medicine, this molecule had a lower ulcerogenic potential and the least activity in causing oxidative stress in tissues. In contrast, pyrazole compounds with a urea pharmacophore exhibited comparable anti-inflammatory effects and better TNF-inhibitory characteristics in the investigations. Moreover, pyrazolyl urea derivatives were investigated for p38 MAPK inhibition and demonstrated substantial efficacy [187] (refer to Figure 7 for Structure and SAR).

An inhibitor of COX-2 with a five-membered pharmacophore, such as pyrazole, and isoxazole, which is polysubstituted 1,3,5-triazines. Compound **181** from synthesized derivatives showed promising findings with an IC_50_ of 0.74 μM vs. celecoxib’s IC_50_ of 0.78 μM. When a methanesulphonylphenyl pyrazole moiety was added, the selectivity increased significantly. To summarize, when the triazine core was replaced with pyrazoline, isoxazole, or unsubstituted pyrazole moieties, a considerable COX-2 selectivity was observed. Thus, scaffolds derived from Compound **181** might be further developed, and their biological activity tested [190] (refer to Figure 7 for Structure and SAR). The pyridazine 6-position was substituted with phenyl, and the carboxyl arm in the central pyrazole was extended by adding polar substituents. In addition, pyrazole compounds without either the 1-pyridazine or the 5-phenyl groups were synthesized. These derivatives were designed to explore how a side chain of different sizes and basicity at three positions of the pyrazole could affect the antiplatelet activity and to learn about the effects of exposure to vicinal diaries about the central heterocycle, as well as the necessary presence of central pyrazoles on their biological profiles. Various compounds of this class had different inhibitory effects on platelet aggregation induced by AA and collagen, mainly depending on the shape and size of the carboxamide molecules at the 3-position of the pyrazole ring. However, only small carboxamide molecules specifically inhibited collagen-induced platelet aggregation. Finally, we describe compound **182** as a new potent antiplatelet agent with an IC_50_ in the two- to one-digit nanomolar range (IC_50_ values of 5.7 nM). Because of their great efficacy in the cellular milieu, these simple pyrazole derivatives may hold promise for developing more effective molecules for cardiovascular disease intervention [191] (refer to Figure 7 for Structure and SAR).

**Figure 8:**
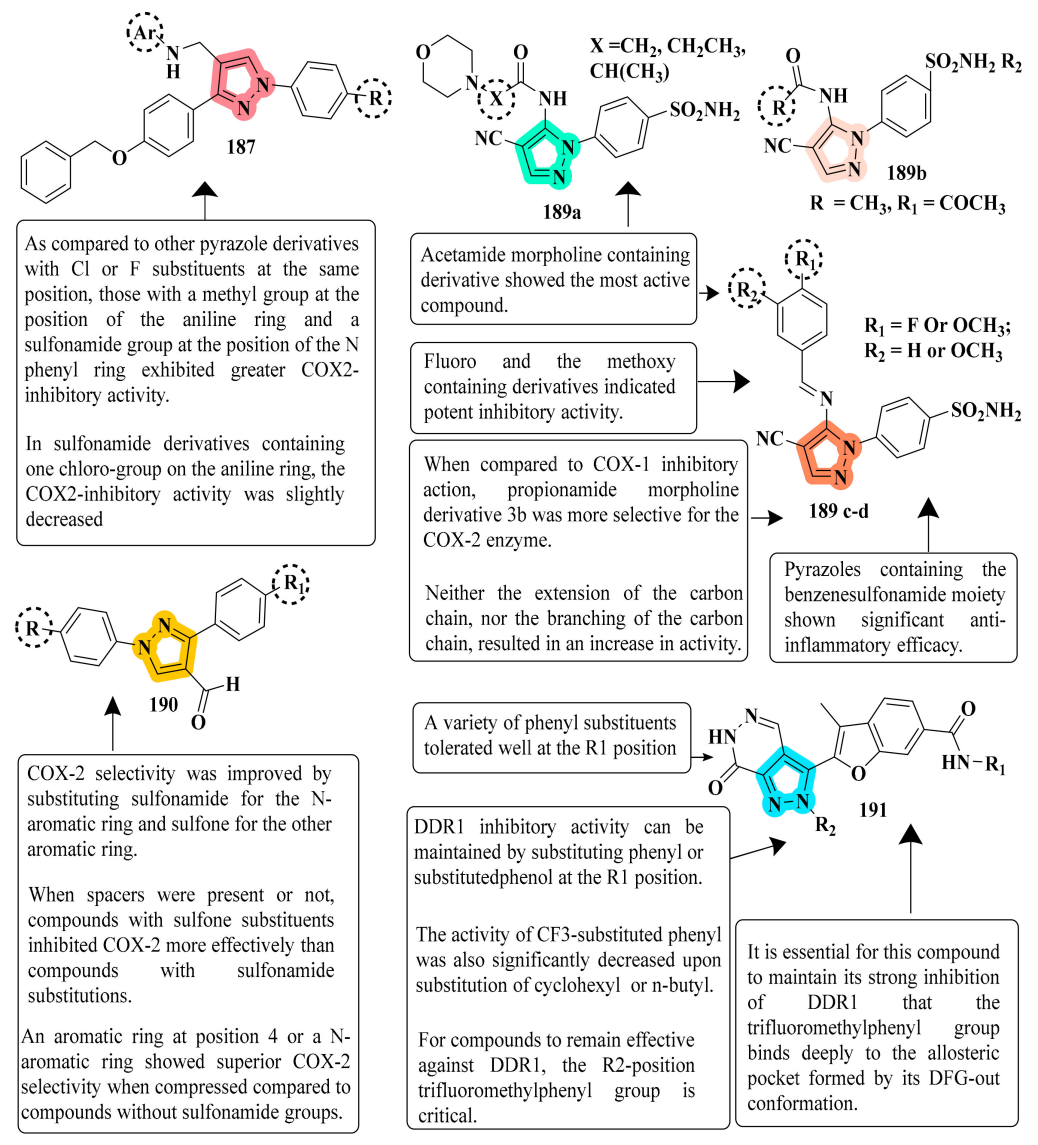
Structure and SAR of potent anti-inflammatory activity compounds **187**, **189**, **190** and **191**.

The primary pyrazole core of selective COX2 inhibitors (celecoxib and SC558) was engaged, and structural alterations led to the identification of additional pyrazole analogs. In comparison to ibuprofen (which inhibited COX-1/2 and TNF- after 4 h and exhibited 81.32 percent inhibition), compound **187** exhibited 80.63 percent inhibition at 4 h. We, therefore, found that methylamine pyrazole derivatives provided the requisite geometry to effectively and selectively inhibit the COX2 enzyme and provide good anti-inflammatory properties when substituted for sulfonamide pyrazole derivatives. As a result, the hydrophobic benzyloxyphenyl group was introduced in the same way as the celecoxib trifluoromethyl group to boost the affinity for COX2 [196] (refer to Figure 8 for Structure and SAR). A novel pyrazole derivative was created and evaluated for its potential to inhibit COX-1/2 activity. There was good selectivity towards COX-2 inhibition in many pyrazoles bearing the benzenesulfonamide moiety, and consequently, these compounds had powerful anti-inflammatory activity. Compounds **189(a**), **189(b**), **189(c**), and **189(d**) showed high activity and selectivity against COX-2 (IC50 = 39.43, 61.24, 38.73, and 39.14 nM). All compounds reported selectivity indices of 22.21, 14.35, 17.47, and 13.10 for **189(a**), **189(b**), **189(c**), and **189(d**), correspondingly. In vivo, these compounds were superior to or equivalent to celecoxib as anti-inflammatory drugs. Interestingly, these molecules specifically reduced the induced microsomal PGE2 synthase synthesis, indicating that additional research into these derivatives is needed [198] (refer to Figure 8 for Structure and SAR).

In an easy synthetic procedure, a series of diaryl pyrazole and triazole derivatives to produce selective COX-2 inhibitors were designed and synthesized. Among the synthesized derivatives, compound **190a** with a sulfonamide substitution on one N-aromatic ring had the strongest inhibitory action against COX-2 with IC_50_ = 0.017 ± 0.001 μM and COX-1 with IC_50_ = 0.263 ± 0.016 μM. In contrast, compound **190b**, which has nitro on its N-aromatic ring, inhibited COX-1 more effectively (IC_50_ = 0.012 ± 0.001 μM). The best COX-2 selectivity was obtained with sulfonamide and sulfone substituted on the N-aromatic ring. More interestingly, the heterocyclic cores (pyrazole or triazole) contain two not vicinal aryls in contrast with the most commonly known COX-2 inhibitors. Additional work is needed to synthesize diverse pyrazoles and triazoles with different spacer lengths and substitutions in order to assess their role in COX-2 inhibitory activity and selectivity [199] (refer to Figure 8 for Structure and SAR). Researchers developed and tested new compounds known as pyrazolo[3,4-d]pyridazinones as anti-inflammatory drugs against discoidin domain receptor 1 (DDR1) modifications. Mouse bone-marrow-derived and human THP-11 macrophages were effectively decreased by compound **191** at ten micromolar concentrations when exposed to LPS. Compound **191** inhibited pro-inflammatory cytokines and autophosphorylation of DDR1 in cells in a mouse colitis model induced by dextran sulfate sodium (DSS). DDR1-IN-1, a positive control compound, had an EC_50_ of 114.5 nM, whereas compound **191** suppressed basal autophosphorylation at 34.4 nM, more powerful than the positive control compound. As the N-phenyl group of DC-1 was exposed above the solvent-exposed area of DDR1, further modifications can be made to this group in DDR1 [200] (refer to Figure 8 for Structure and SAR).

## 5. Conclusions

This literature survey focused on merging different pharmacophoric subunits of a large number of pyrazolic analogues to create potential lead compounds showing anticancer and anti-inflammatory activities. It also highlighted the prestige of these biomolecules in the design, discovery, and advancement of novel drug molecules.

## Figures and Tables

**Table 1:** Representative drugs containing the pyrazole scaffold.

S. No.	Name	Structure	Category
1	Celecoxib [6]	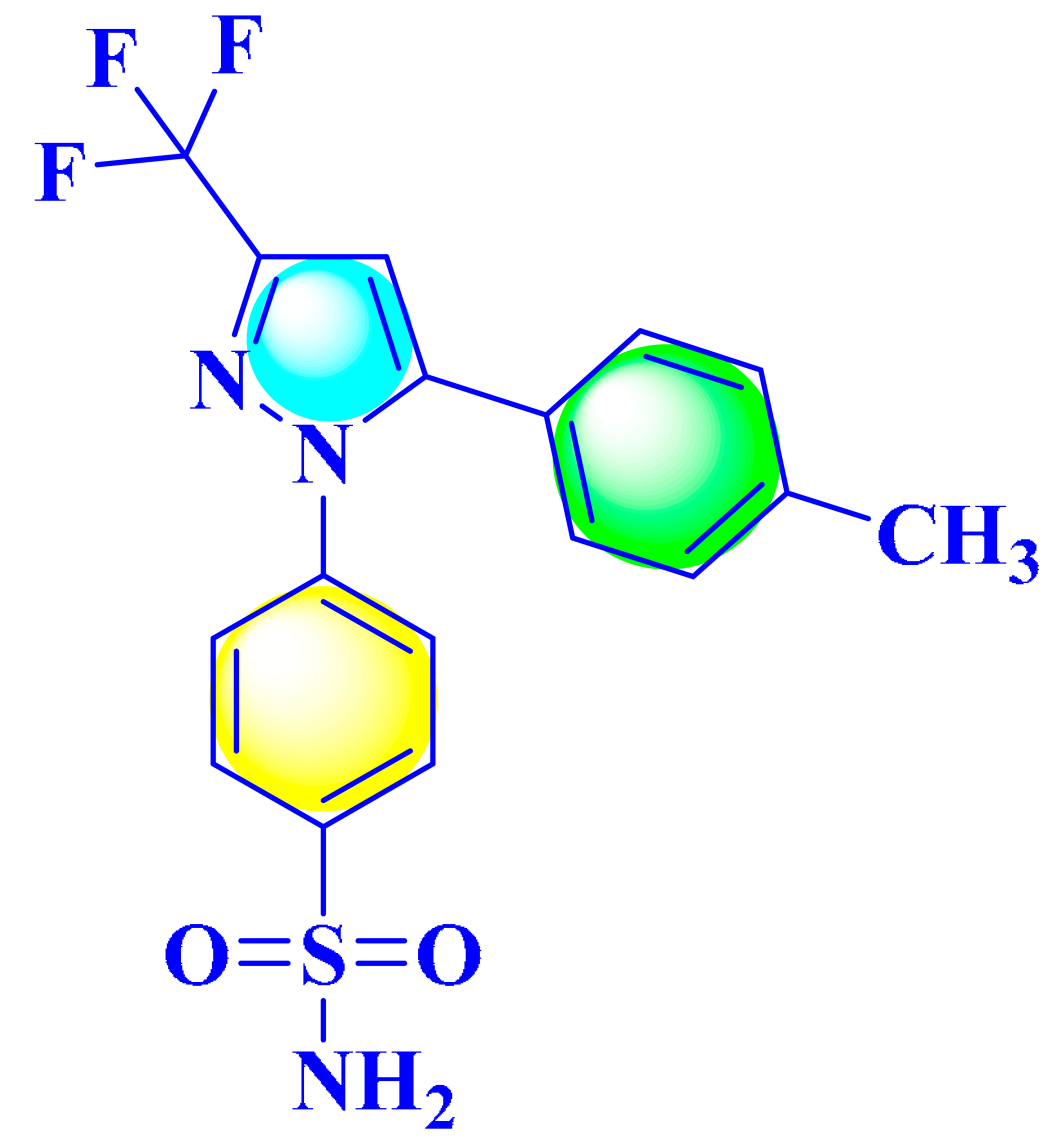	Anti-inflammatory
2	Lonazolac [7]	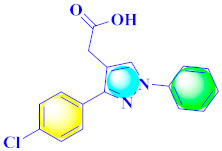	NSAID
3	Tepoxalin [8]	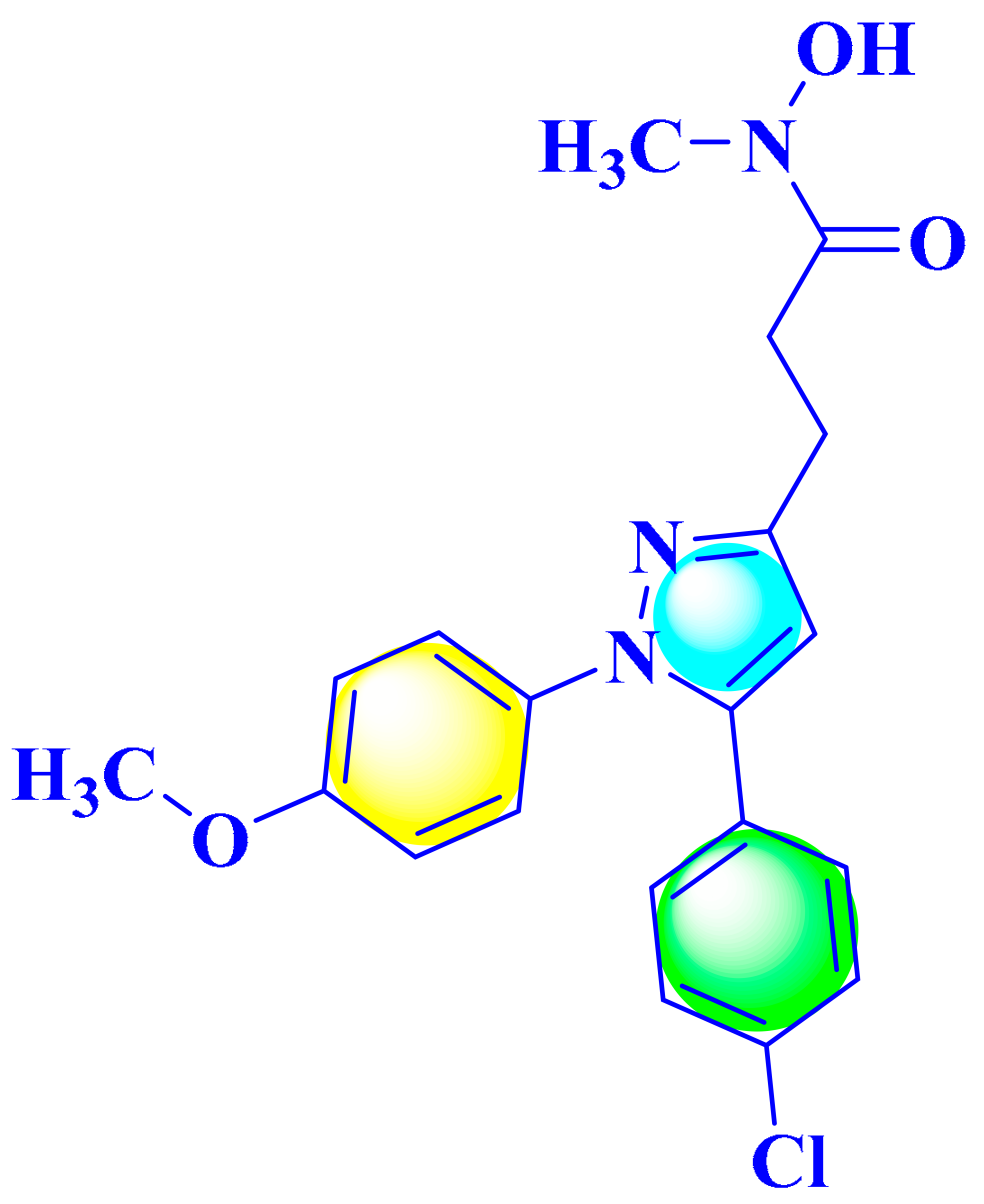	NSAID
4	Antipyrine [9]	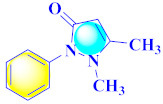	NSAID
5	Phenylbutazone [10]	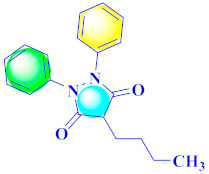	NSAID
6	Novalgin [11]	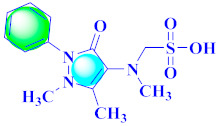	Analgesic, antipyretic, and antispasmodic
7	Ramifenazone [12]	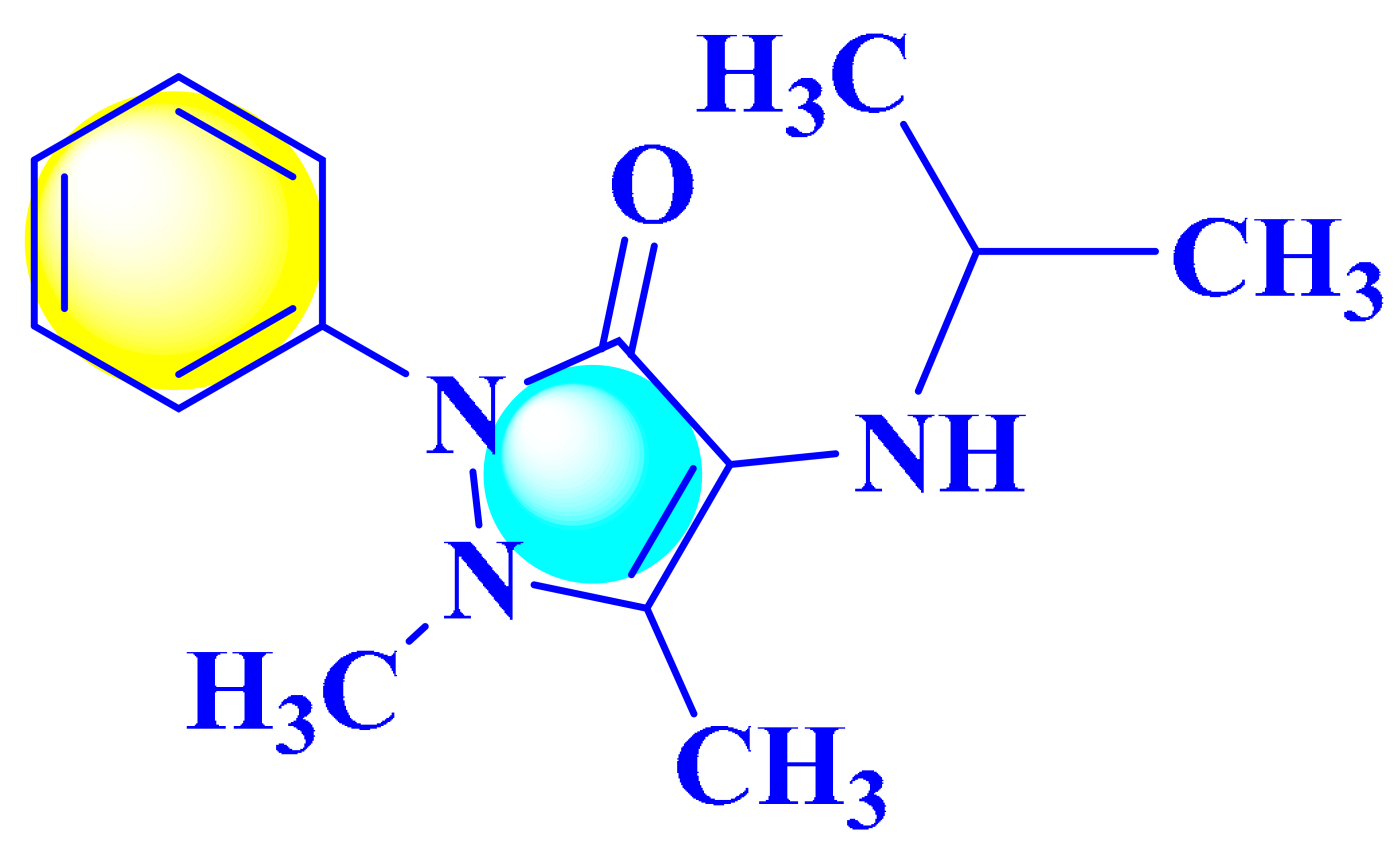	NSAID
8	Deracoxib [13]	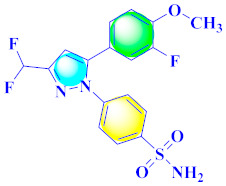	NSAID
9	Difenamizole [14]	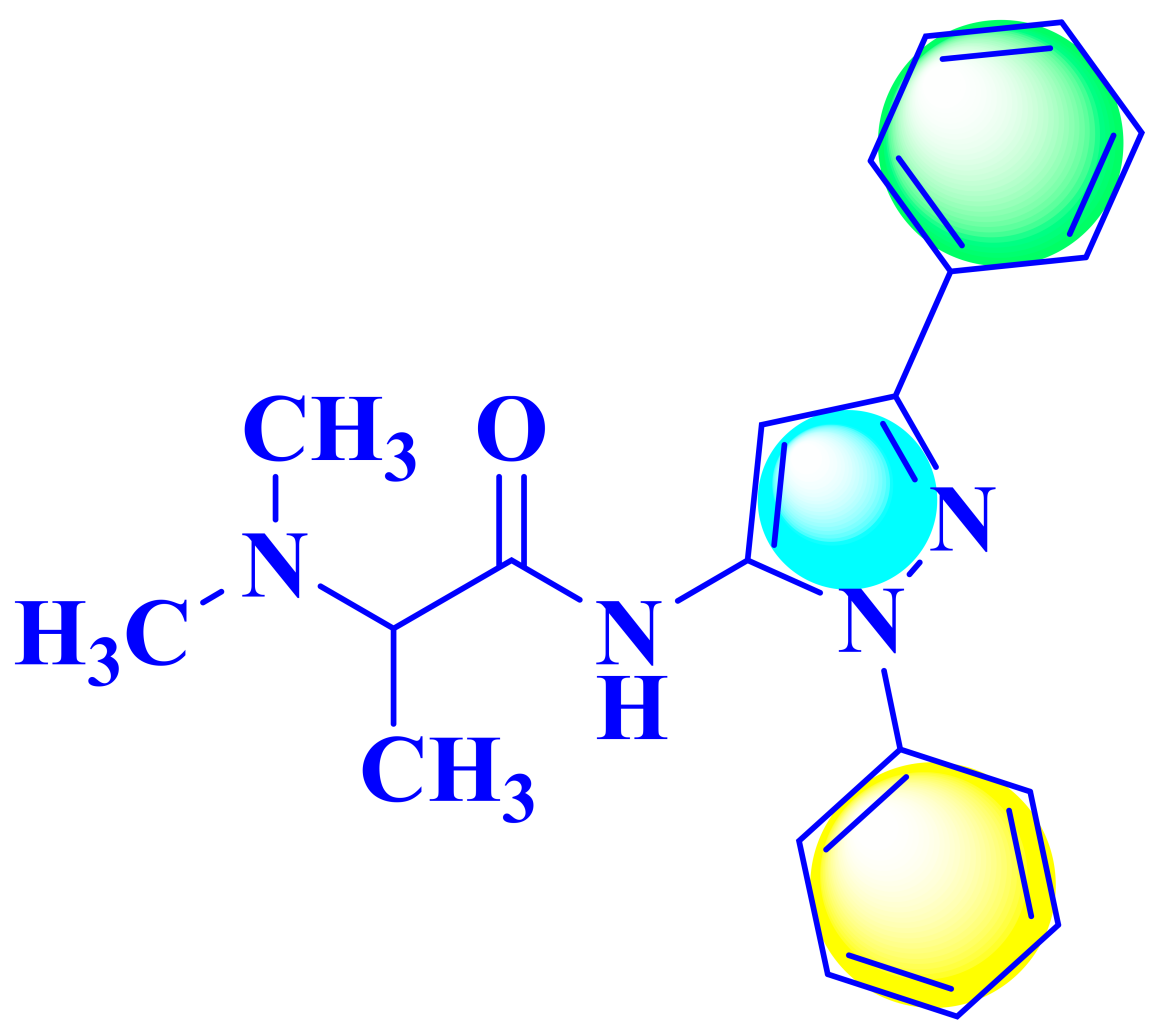	NSAID
10	Mepirizole [15]	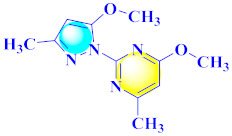	NSAID
11	SC-560 [16]	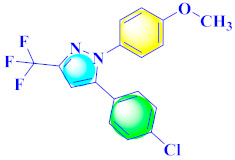	Cyclooxygenaseinhibitor
12	Crizotinib [17]	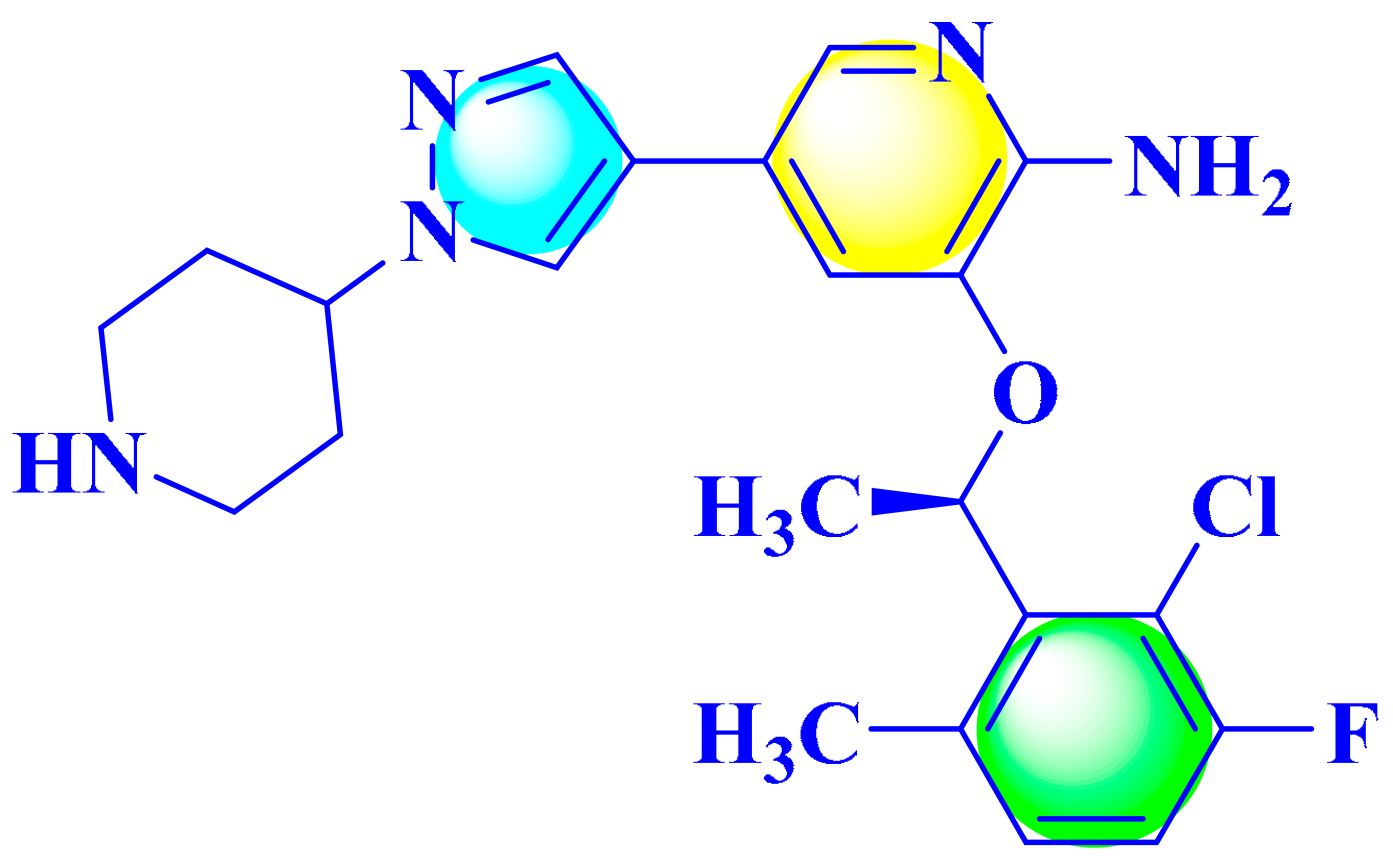	Anticancer
13	Pyrazofurin [18]	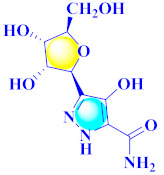	Anticancer
14	Tartrazine [19]	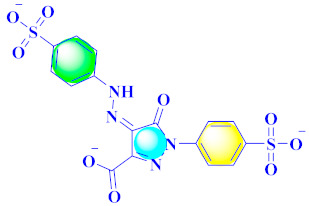	Anticancer

**Table 2:** Representative compound structures **2** to **18** and their anticancer activity.

Compounds	Structures	Anticancer Activity	
		Cancer Cell Line	Biological Activity Value
**2**	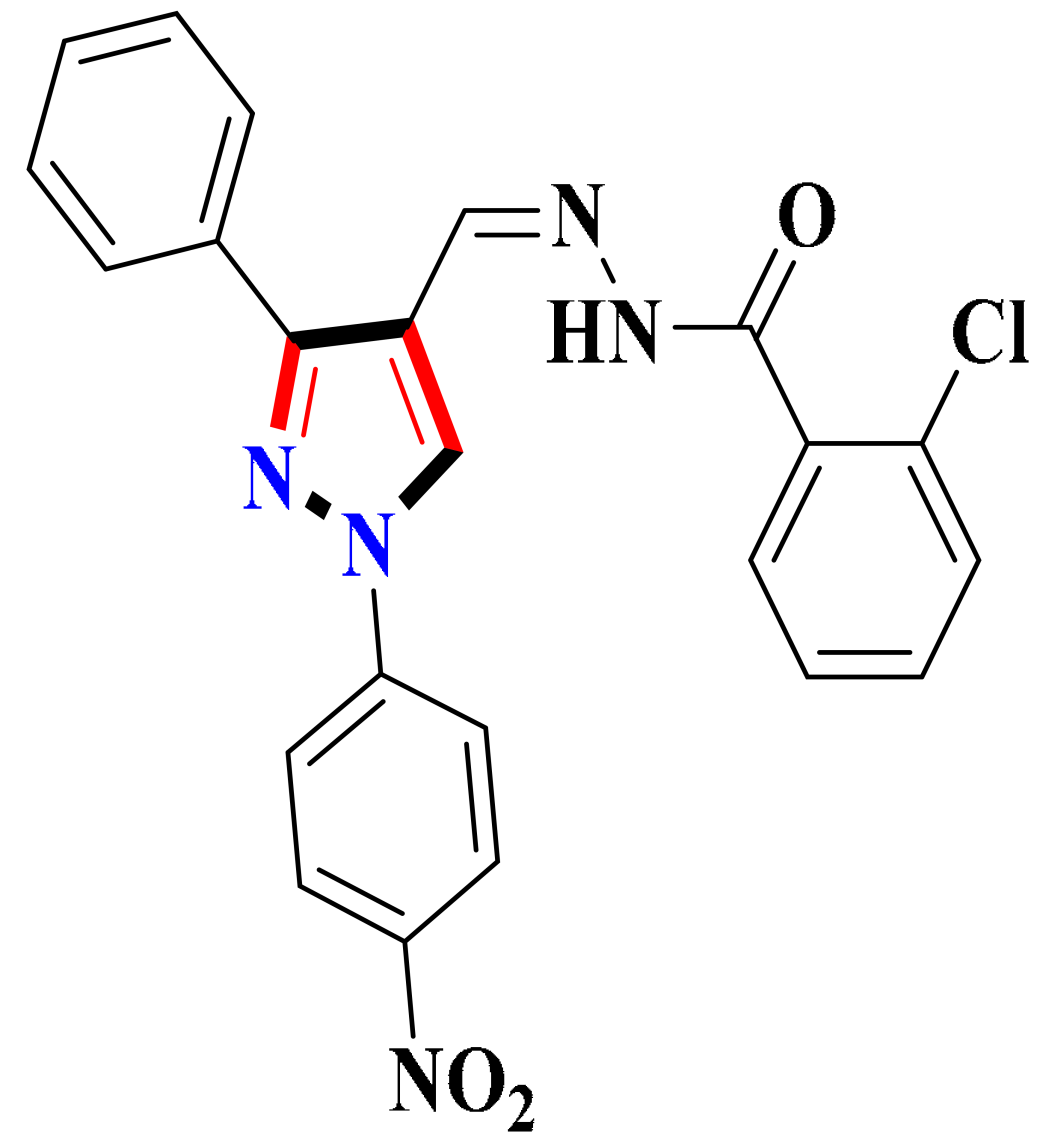	MCF7	IC_50_ = 3.79 µM
		SF-268	IC_50_ = 12.50 µM
		NCI-H460	IC_50_ = 42.30 µM
**3**	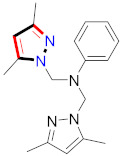	Hep-2	IC_50_ = 3.25 mg/mL
		P815	IC_50_ = 17.82 mg/ml
**4**	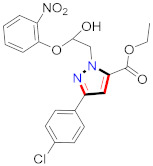	A549	IC_50_ = 26 µM
**5**	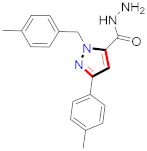	A549	IC_50_ = 49.85 µM
**6**	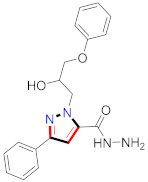	NCIH460	IC_50_ = 48 µM
**7**	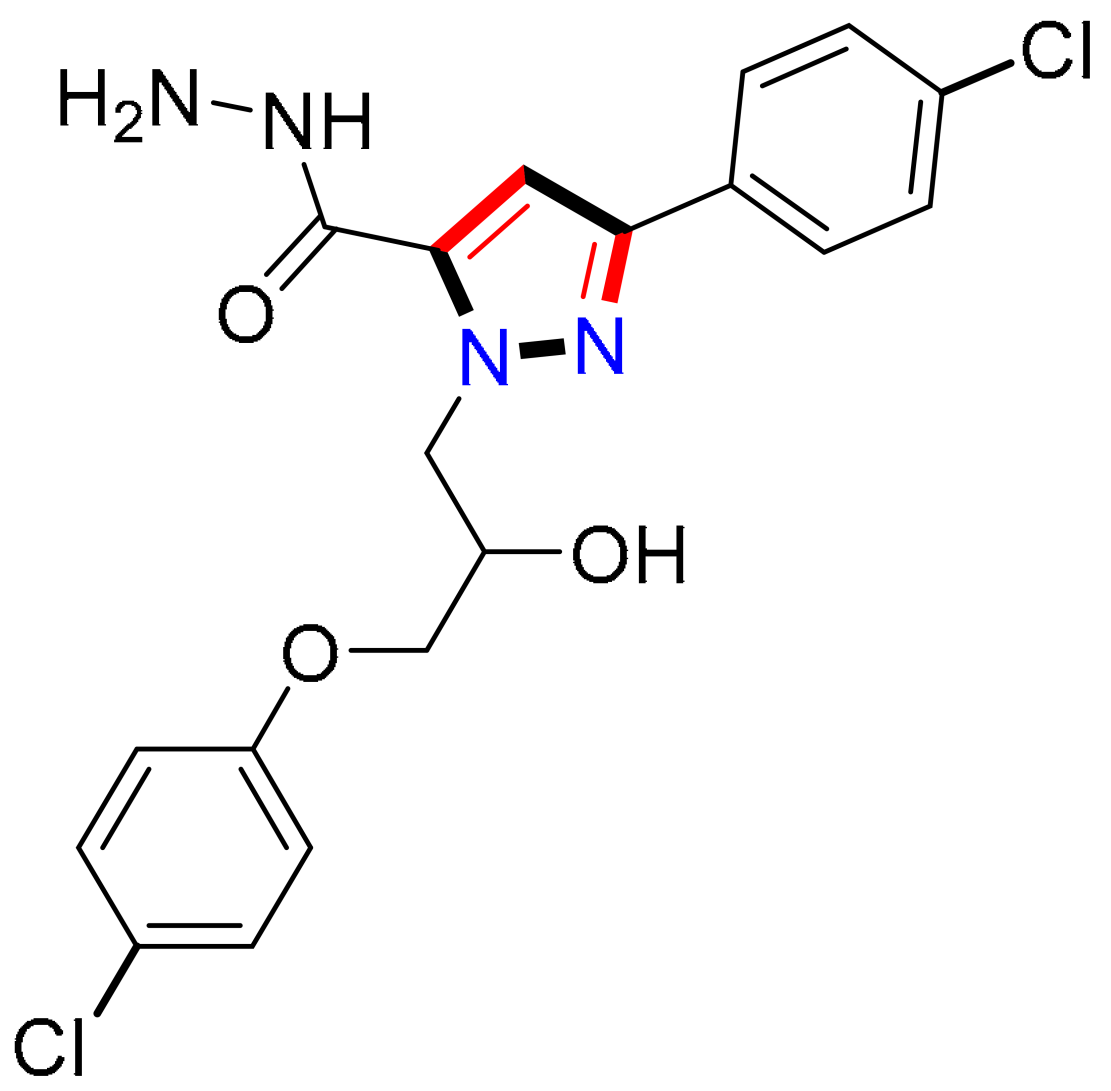	NCIH460	IC_50_ = 32 µM
**8**	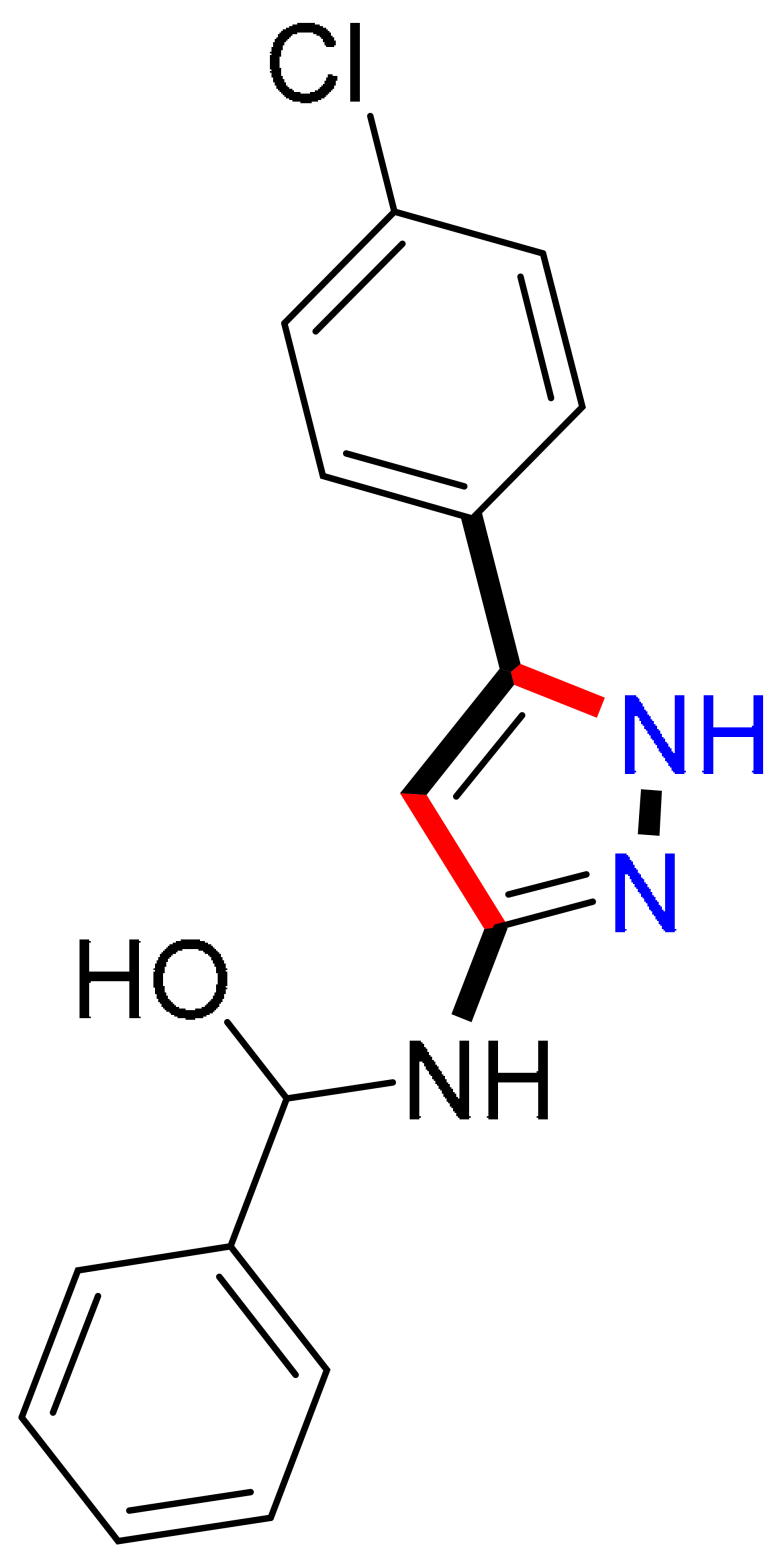	Hep-2	IC_50_ = 0.74 mg/mL
**9**	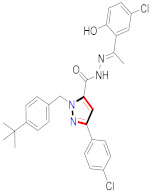	A549	IC_50_ = 0.28 µM
**10**	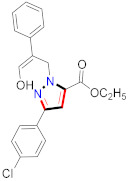	A549	IC_50_ = 14.5 µM
**11**	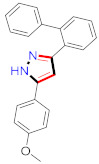	NCI-H460	GI_50_ = 0.67 µM
		SW620	GI_50_ = 0.89 µM
		OVCA	GI_50_ = 0.73 µM
		AGS	GI_50_ = 0.79 µM
**12**	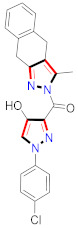	TGI	GI_50_ = 8.78 µM
		MG-MID	LC_50_ = 69.3 µM
**13**	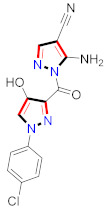	TGI	GI_50_ = 30.9 µM
		MG-MID	LC_50_ = 93.3 µM
**14**	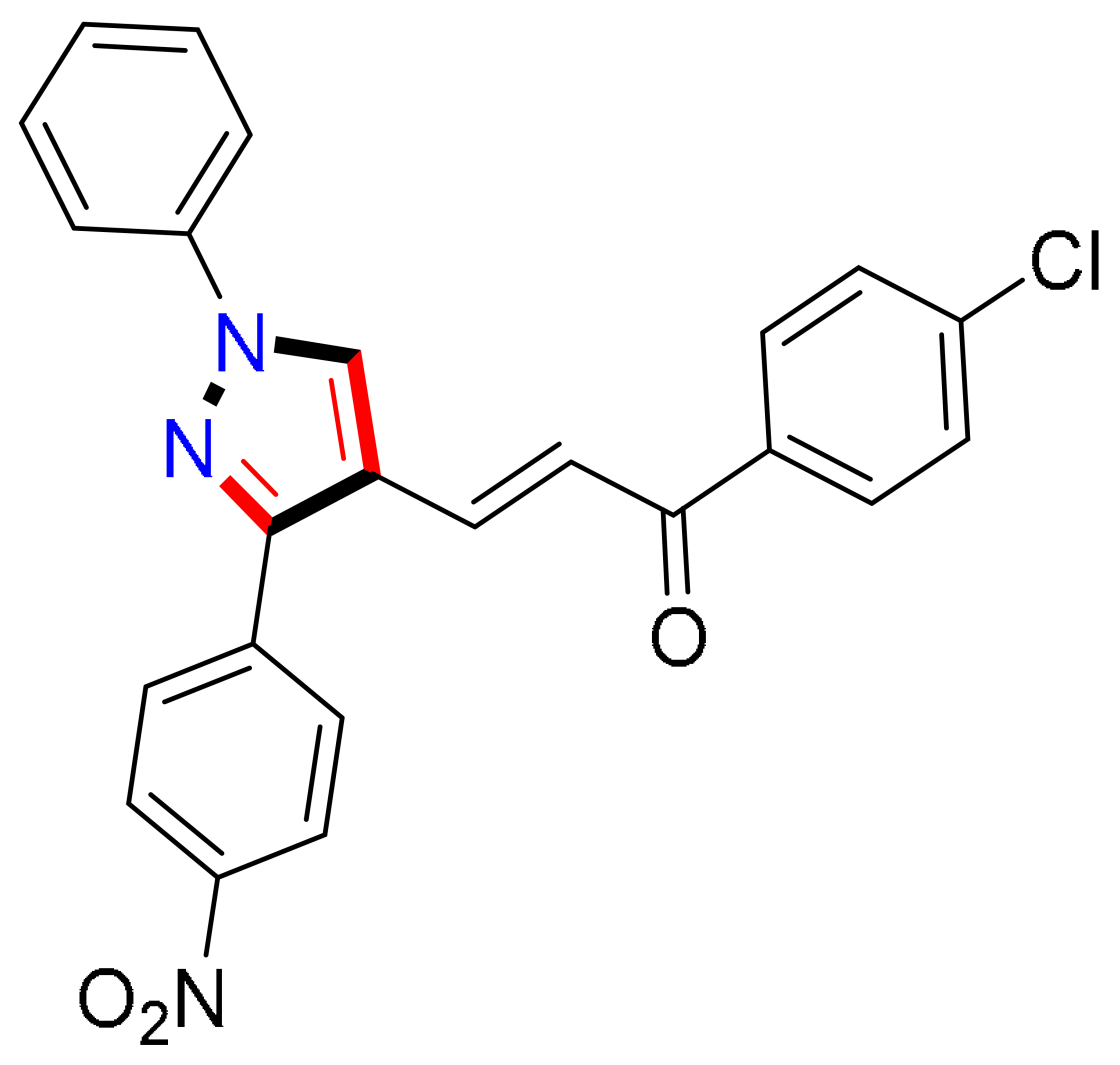	K-562	GI_50_ = 0.09 µM
		SR	GI_50_ = 2.76 µM
		UO-31	GI_50_ = 0.10 µM
**15**	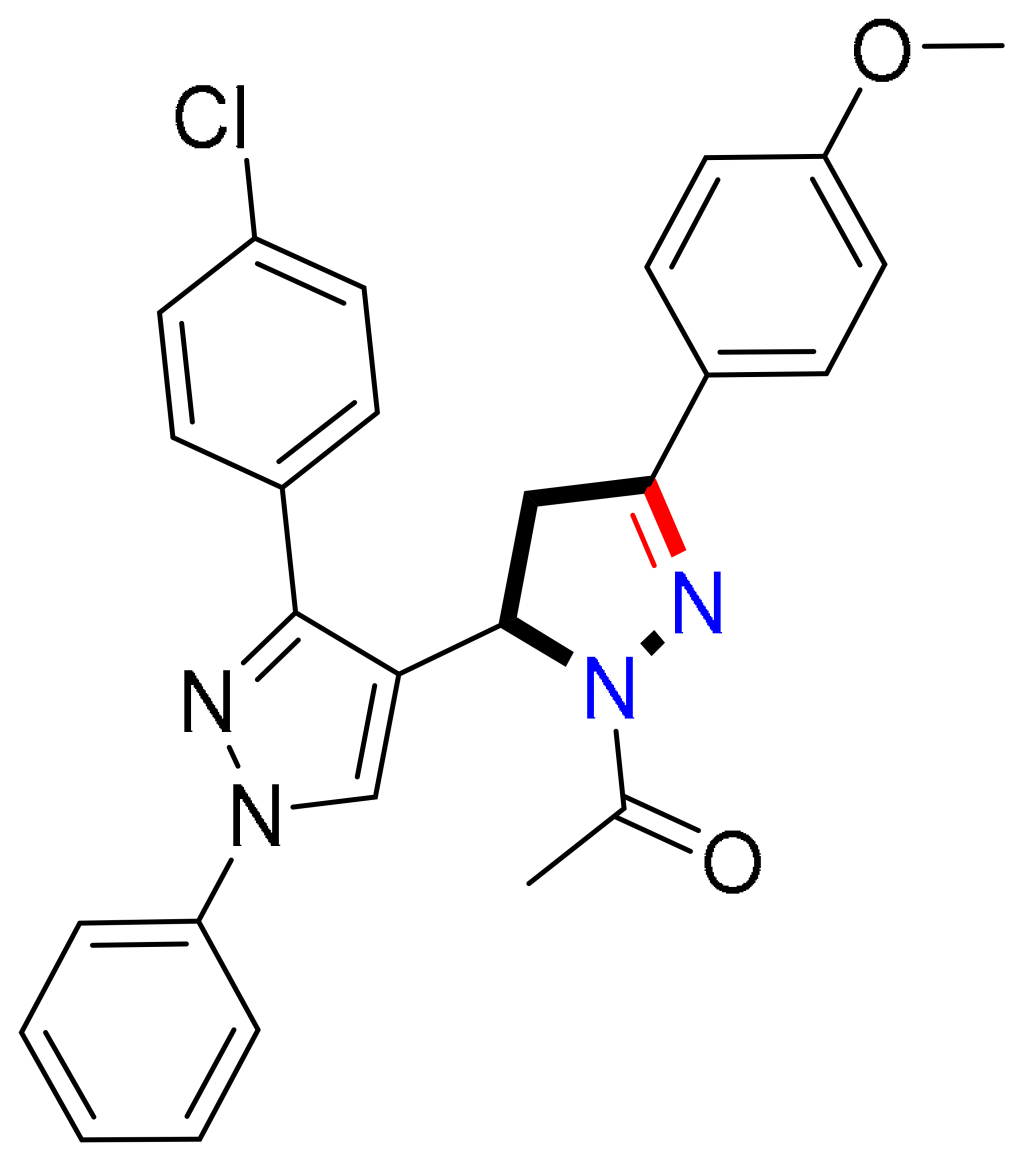	SR	IC_50_ = 1.57 µM
		HOP-92	IC_50_ = 0.04 µM
**16**	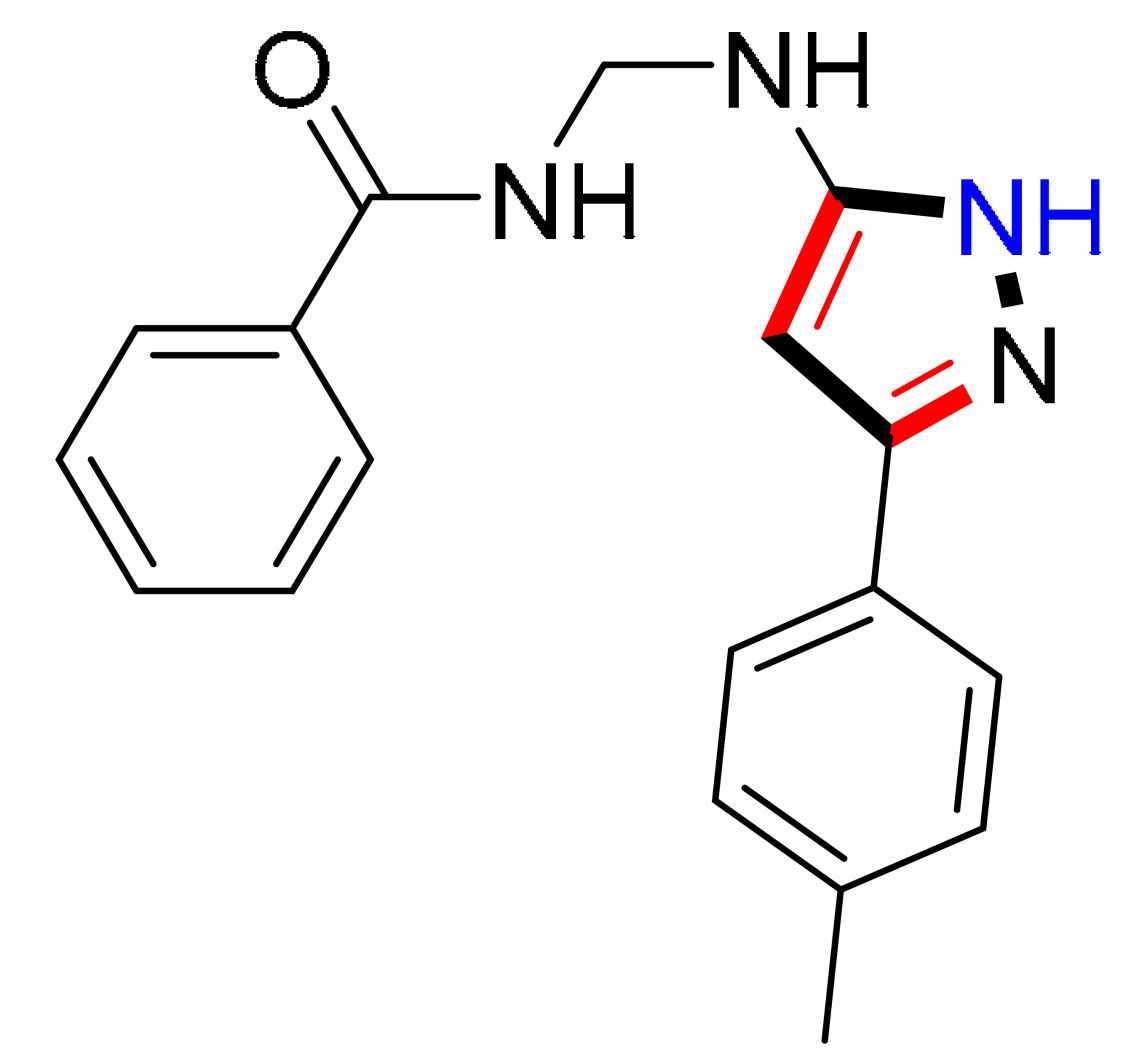	HCT-116	Log GI_50_ = −5.75
**17**	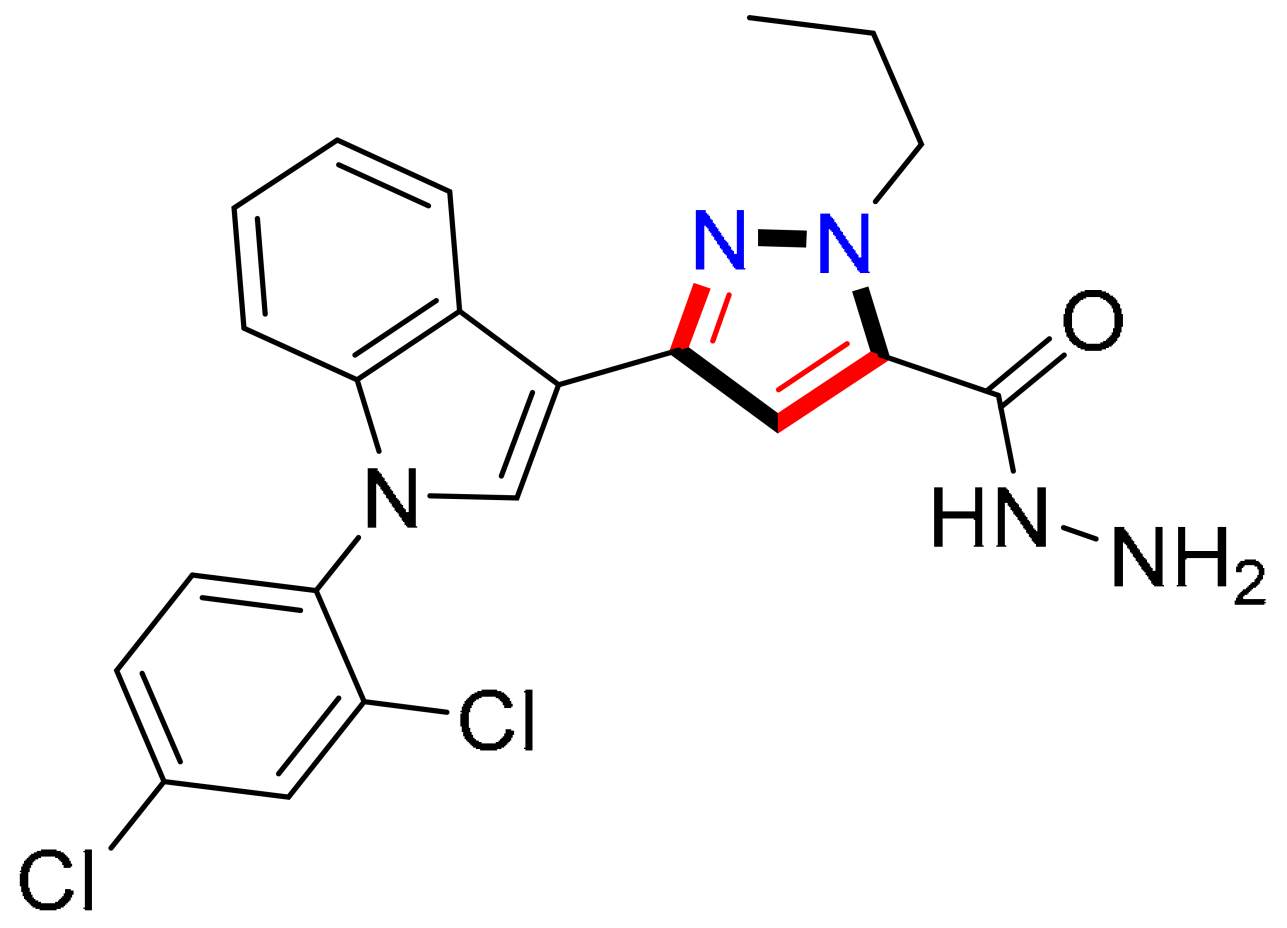	HepG-2	IC_50_ = 0.71 μM
		BT474	IC_50_ = 1.39 μM
		BGC823	IC_50_ = 0.71 μM
**18**	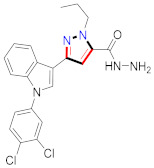	HepG-2	IC_50_ = 0.71 μM
		BT474	IC_50_ = 1.39 μM
		BGC823	IC_50_ = 0.71 μM

**Table 3:** Representative compound structure **19** to **30** and their anticancer activity.

Compounds	Structure	Anticancer Activity	
		Cancer Cell Line	Biological Activity Value
**19**	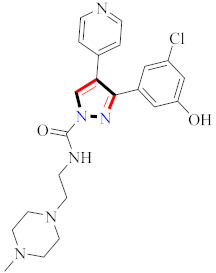	A375	IC_50_ = 4.2 µM
**20**	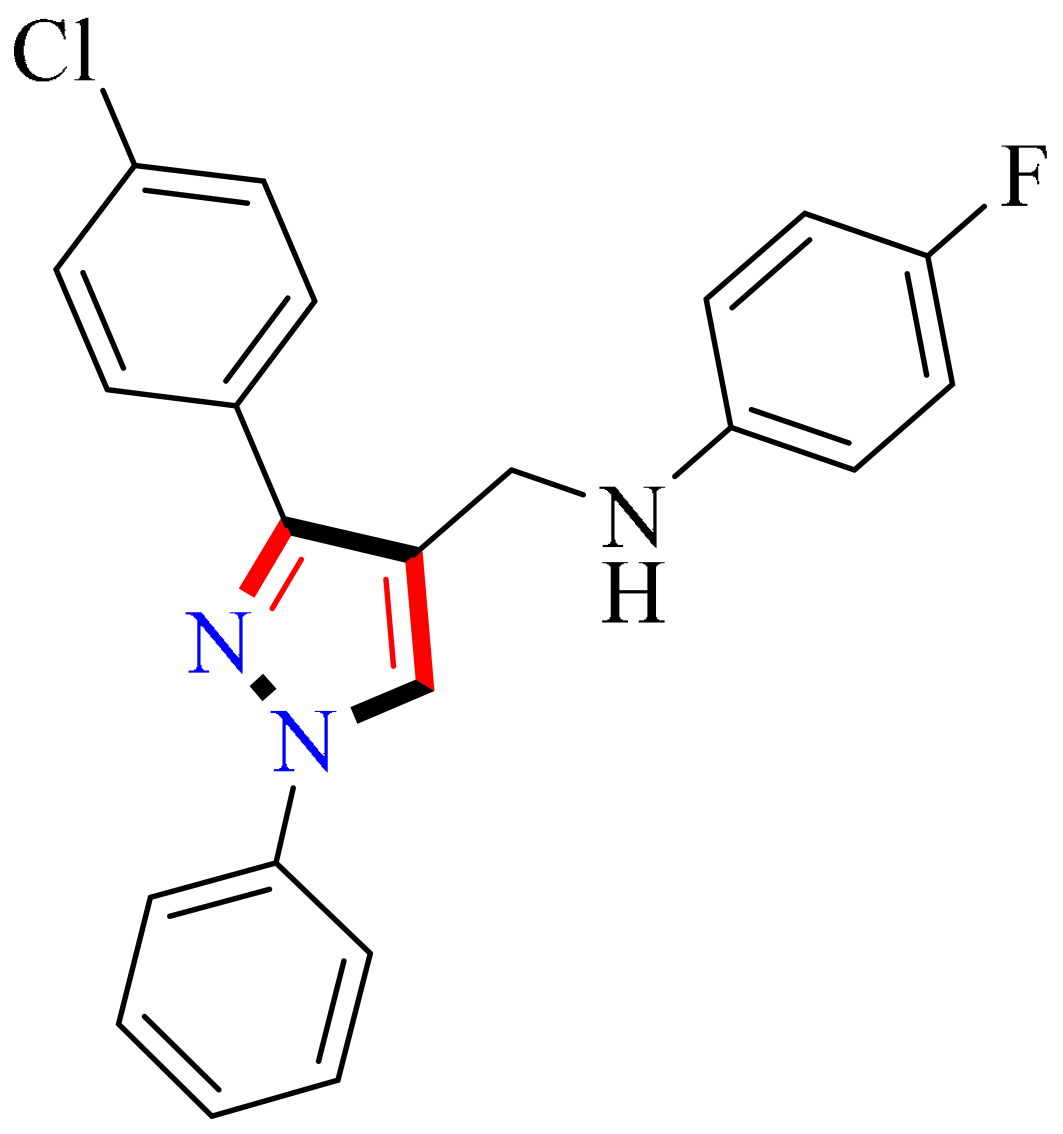	MCF-7	IC_50_ = 1.88 µM
		B16-F10	IC_50_ = 2.12 µM
**21**	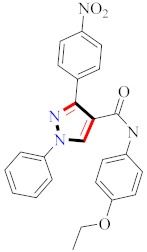	MCF-7	IC_50_ = 0.46 µM
		HCT116	IC_50_ = 0.39 µM
		Aurora-A kinase	IC_50_ = 0.16 µM
**22**	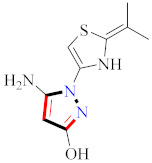	MCF7	IC_50_ = 0.01 µM
		NCI-H460	IC_50_ = 0.03 µM
		SF-268	IC_50_ = 31.5 µM
**23**	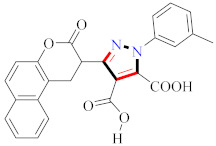	MCF7	IC_50_ = 0.01 µM
		NCI-H460	IC_50_ = 0.03 µM
		SF-268	IC_50_ = 31.5 µM
**24**	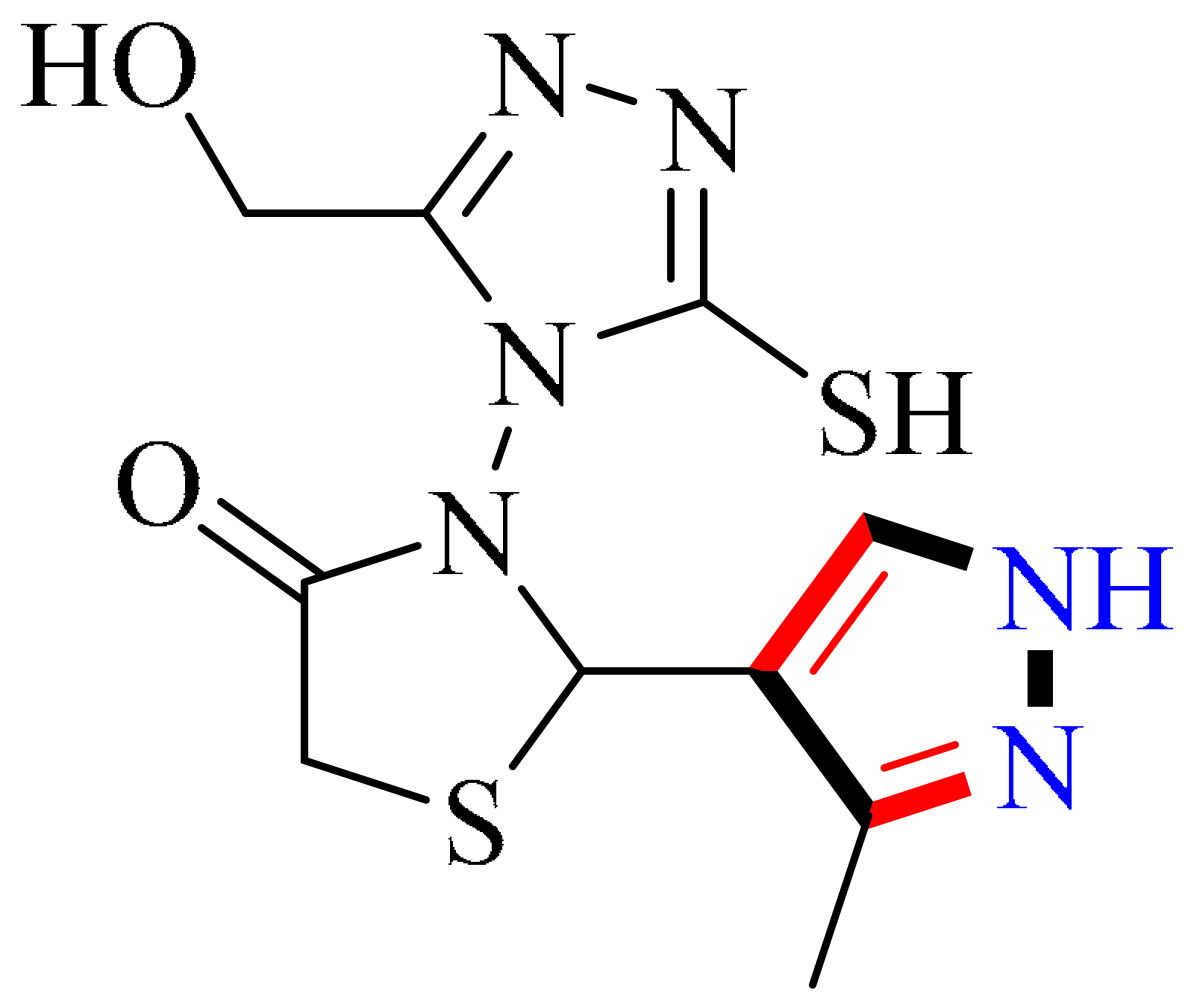	CDK2	IC_50_ = 25 nM
**25**	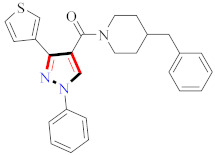	Raji	IC_50_ = 25.2 µM
		HL60	IC_50_ = 28.3 µM
**26**	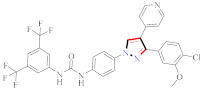	NCI-60 inhibition activity	128.13%
**27**	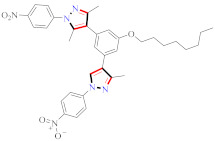	Bel-7402	-
		HL-60	-
		BGC-823	-
**28**	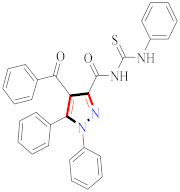	HepG2	% cell viability after 24 = 38.17
		Jurkat	% cell viability after 24 = 23.30
**29**	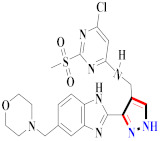	U937	IC_50_ = 5.106 µM
		K562	IC_50_ = 5.003 µM
		HT29	IC_50_ = 0.381 µM
		A549	IC_50_ = 0.487 µM
**30**	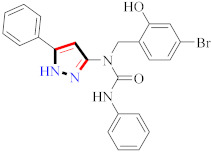	WM266.4	IC_50_ = 1.50 µM
		A375	IC_50 =_ 1.32 µM
		BRAF (V600E)	IC_50_ = 0.19 µM

**Table 4:** Representative compound structures **31** to **47** and their anticancer activity.

Compounds	Structure	Anticancer Activity	
		Cancer Cell Line	Biological Activity Value
**31**	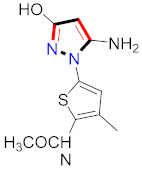	MCF-7	GI_50_ = 11.8 µM
		NCI-H460	GI_50_ = 14.5 µM
		SF268	GI_50_ = 16.7 µM
**32**	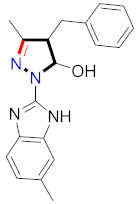	PCA-1/ALKBH3	IC_50_ = 0.67 µM
**33**	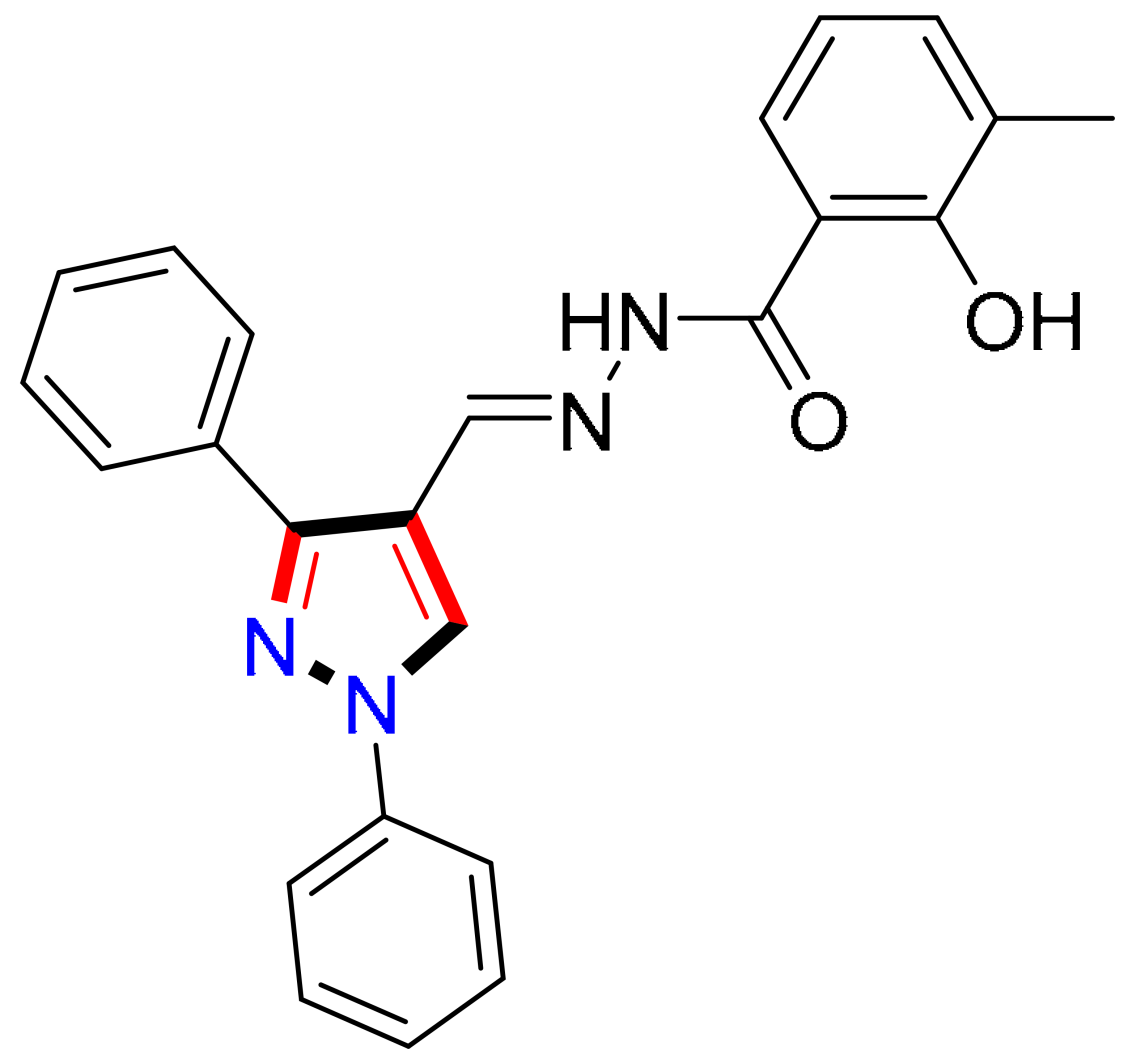	B16-F10	IC_50_ = 0.49 µM
		MCF-7	IC_50_ = 0.57 µM
**34**	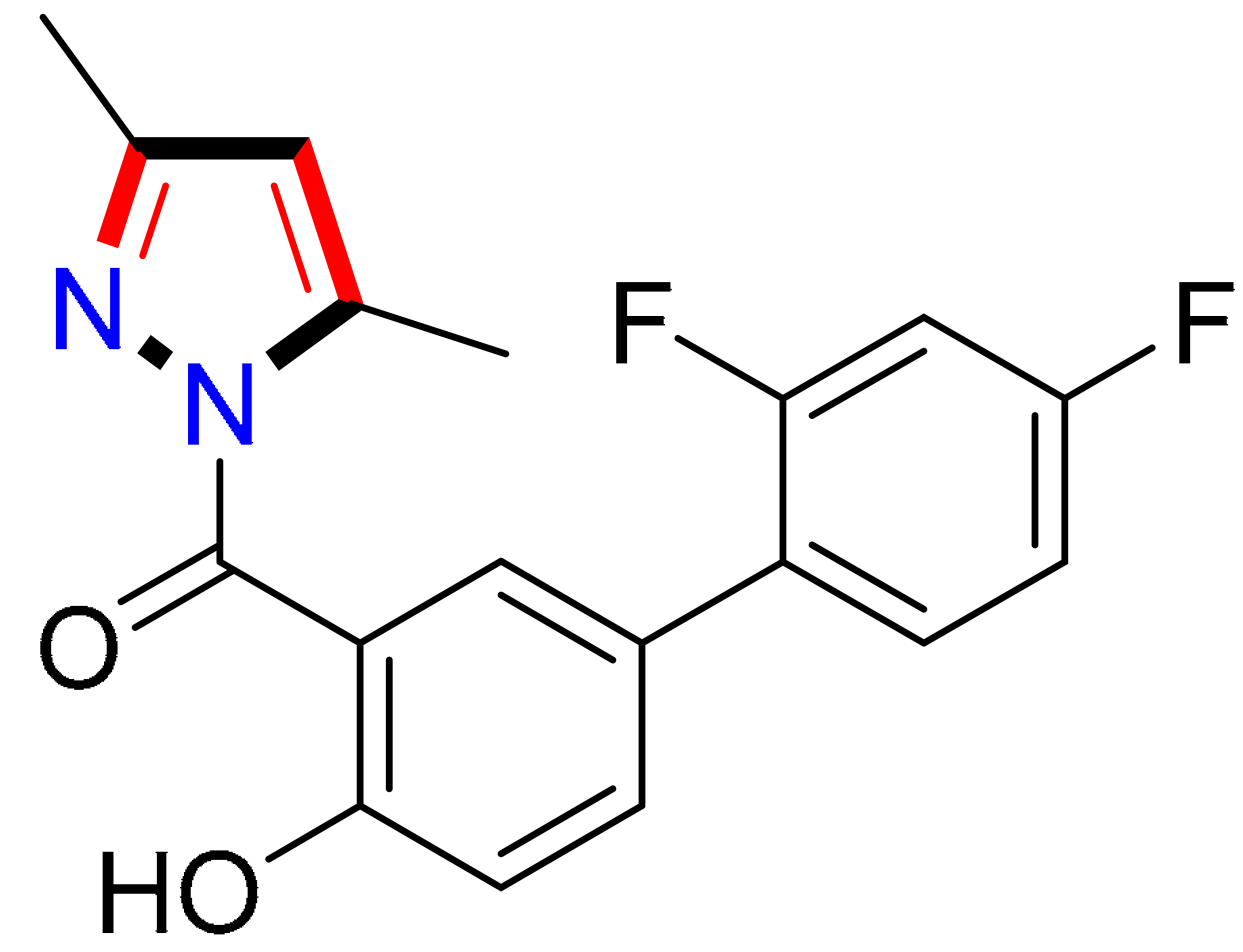	K-562	IC_50_ = 4 µM
**35**	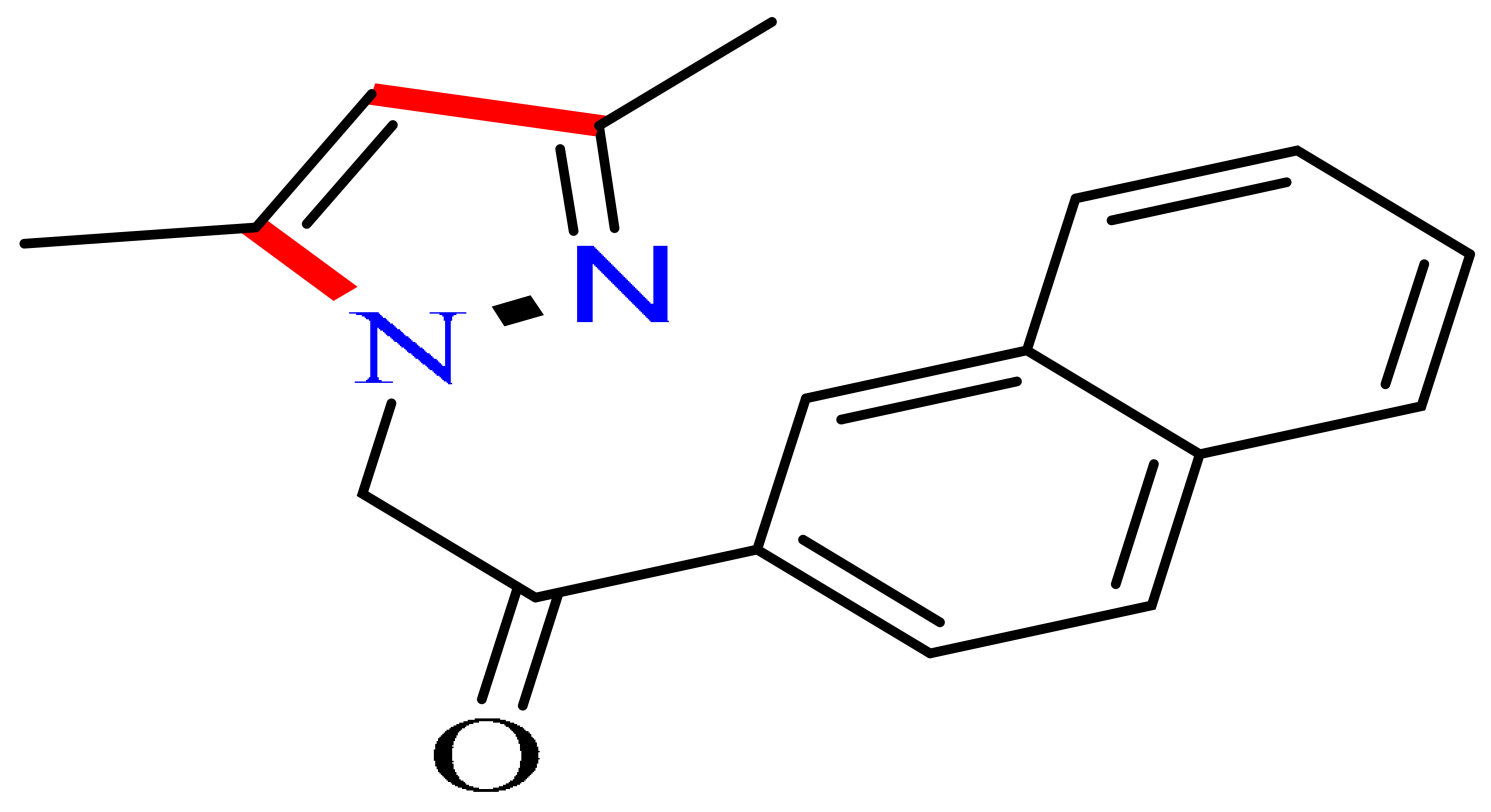	HCT-116	IC_50_ = 56.83 µM
**36**	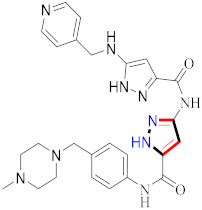	HepG2	IC_50_ = 32.23 µM
		HCT116	IC_50_ = 10.93 µM
		CDK2	IC_50_ = 9.5 µM
		EGFR	IC_50_ = 13.51 µM
**37**	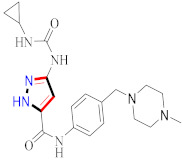	HepG2	IC_50_ = 32.33 µM
		HCT116	IC_50_ = 15.94 µM
		CDK2	IC_50_ = 7.23 µM
		EGFR	IC_50_ = 3.31 µM
**38**	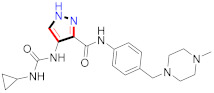	A375	IC_50_ = 8.50 µM
**39**	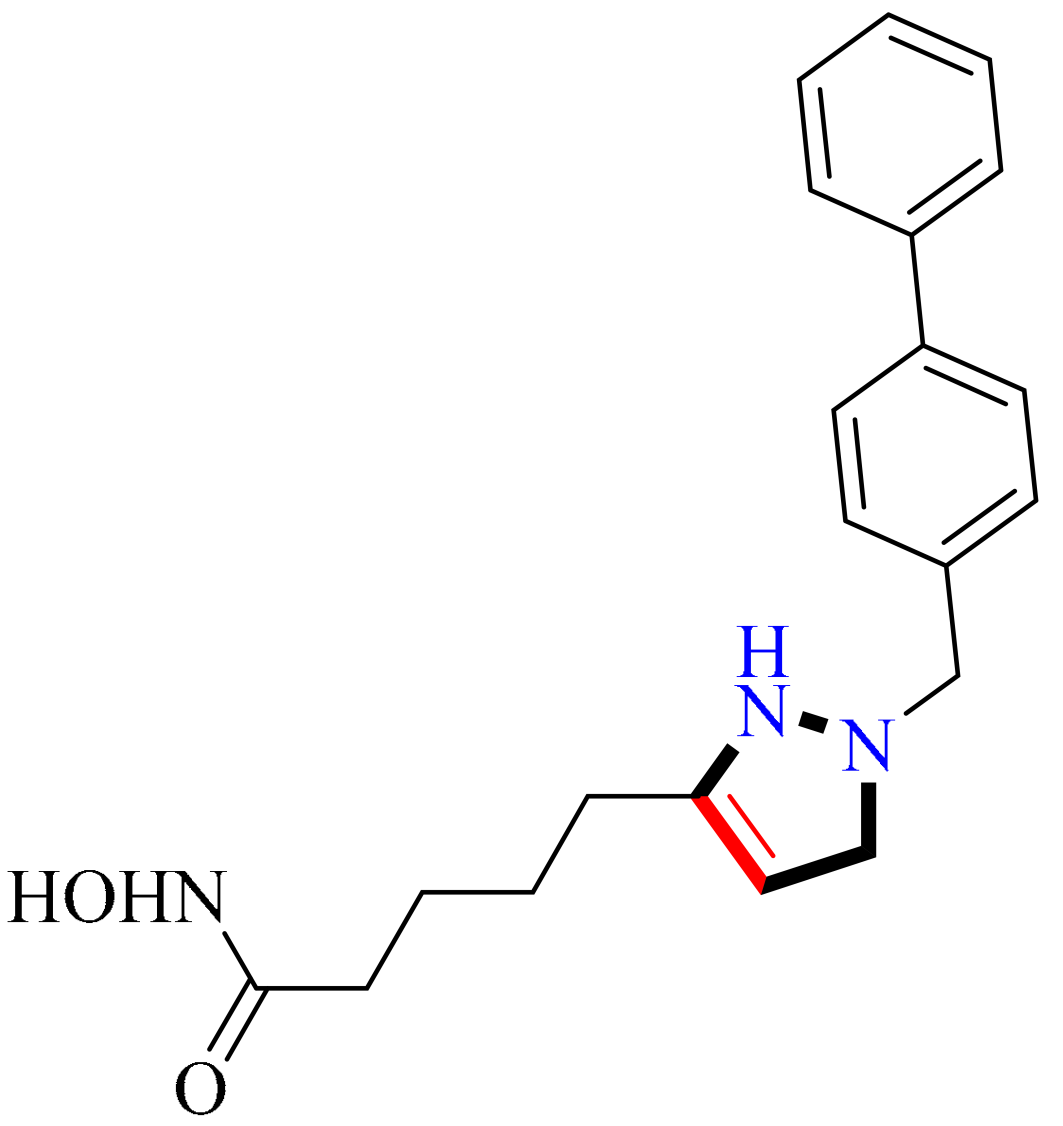	HDAC1	IC_50_ = 0.033 µM
**40**	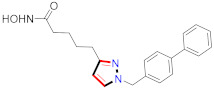	HeLa	IC_50_ = 0.043 µM
		HDAC1	IC_50_ = 0.225 µM
**41**	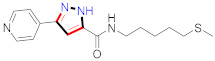	HCT-116	GI_50_ = 8.93 µM
**42**	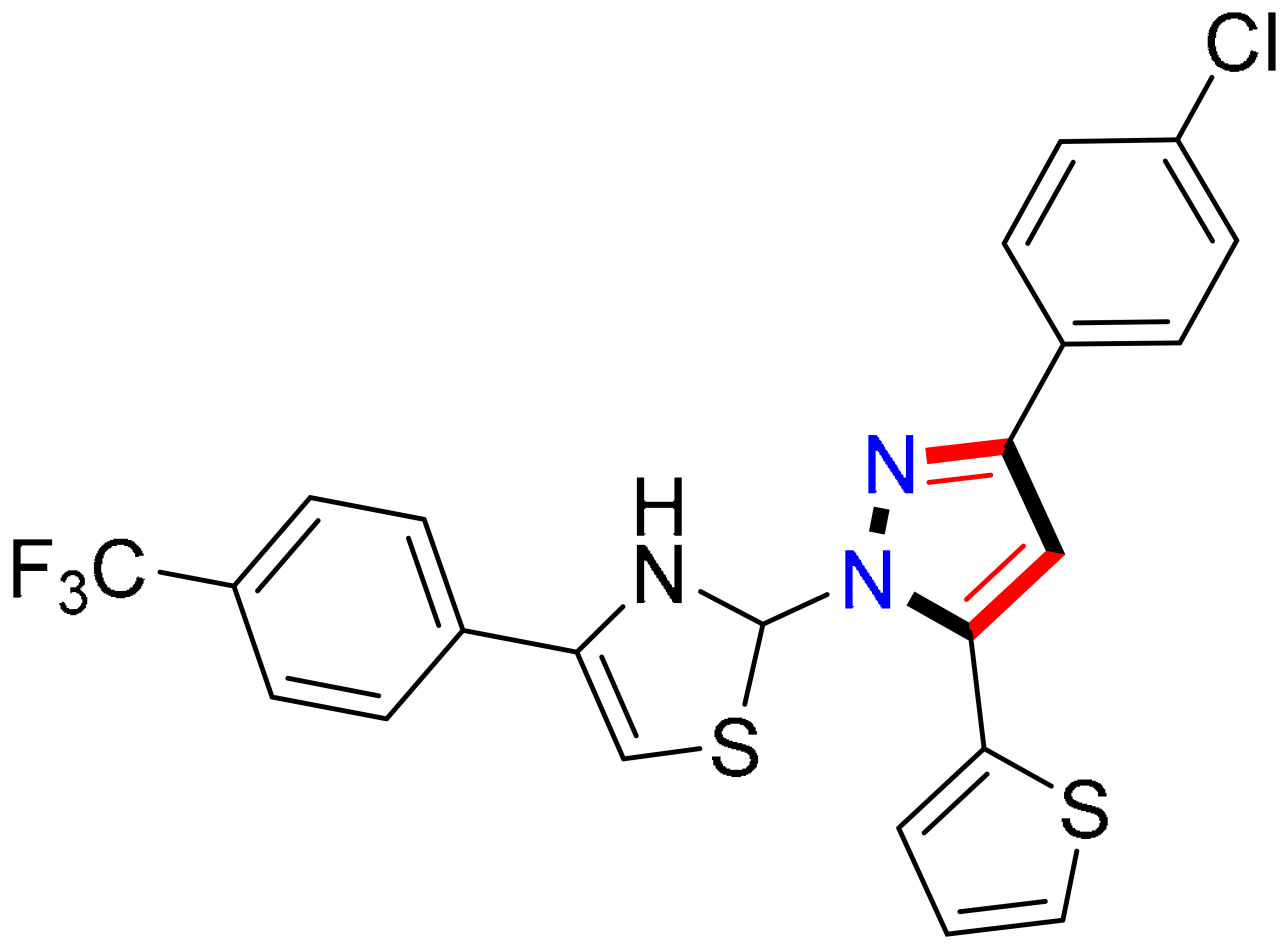	WM266.4	IC_50_ = 0.12 µM
		MCF-7	IC_50_ = 0.16 µM
**43**	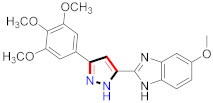	HepG2	IC_50_ = 1.8 µM
		A549	IC_50_ = 0.7 µM
		MCF7	IC_50_ = 1.6 µM
		HeLa	IC_50_ = 0.9 µM
		DU-145	IC_50_ = 2.2 µM
**44**	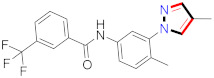	BCR-Bal kinase	IC_50_ = 14.9 nM
**45**	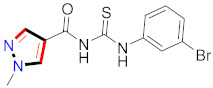	THP-1	IC_50_ = 72.9 µM
**46**	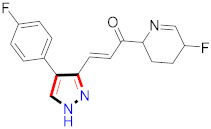	HeLa	IC_50_ = 0.018 µM
		MCF-7	IC_50_ = 0.047 µM
**47**	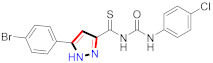	HT-29	G0/G1 at 50 µM = 4.17

**Table 5:** Representative compound structures **48** to **64** and their anticancer activity.

Compounds	Structure	Anticancer Activity	
		Cancer Cell Line	Biological Activity Value
**48**	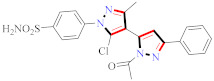	hCA I	Ki = 20.7 nM
**49**	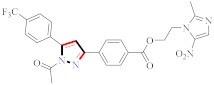	EGFR	IC_50_ = 0.26 µM
		HER-2	IC_50_ = 0.20 µM
**50**	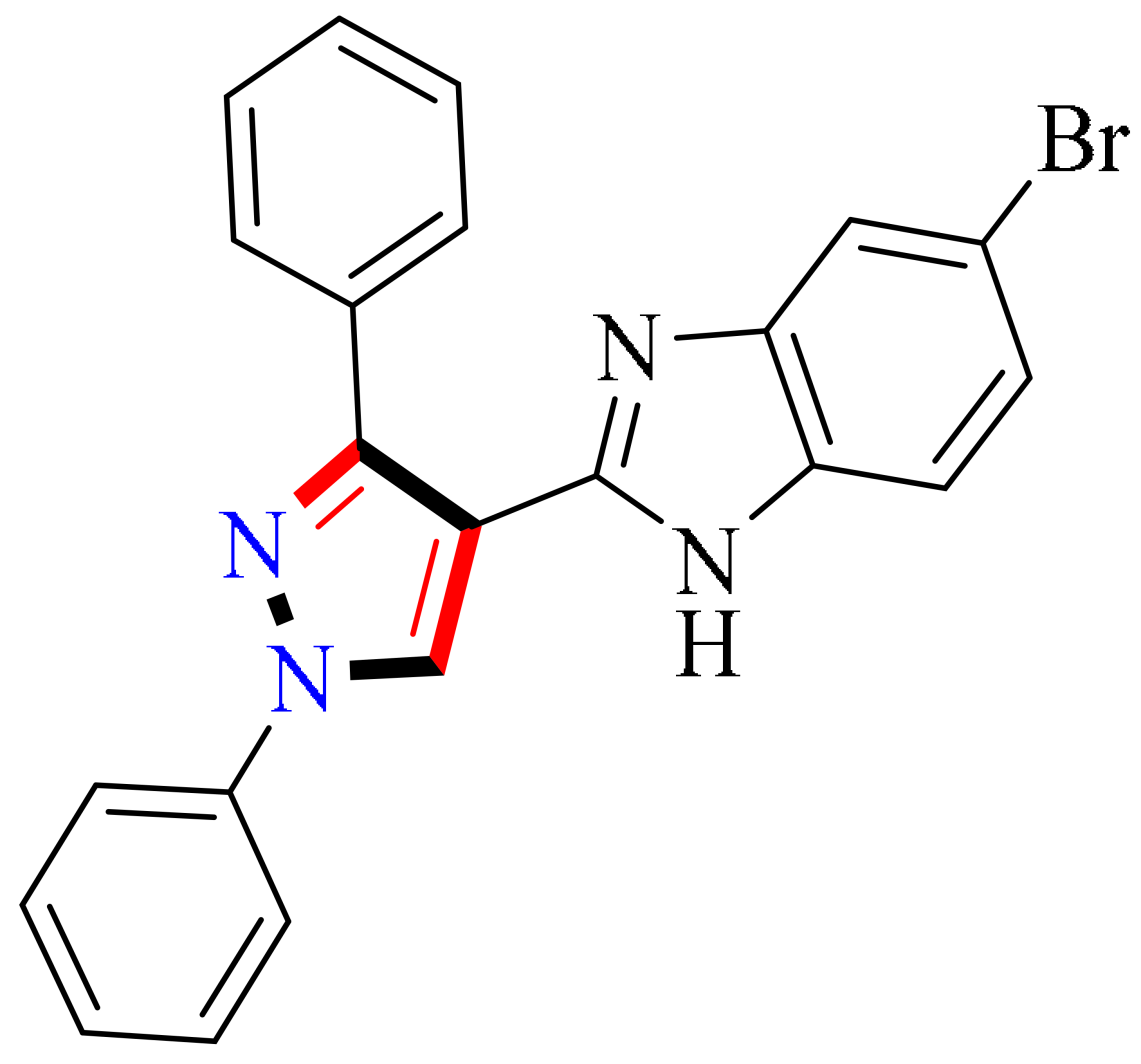	MCF-7	IC_50_ = 0.83 µM
		HeLa	IC_50_ = 1.76 µM
		A549	IC_50_ = 1.81 µM
**51**	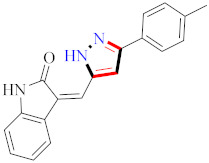	Tubulin polymerization	IC_50_ = 8.10 µM
**52**	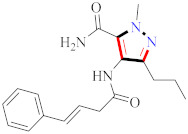	SGC-7901	IC_50_ = 8.11 µM
		MGC-803	IC_50_ = 3.44 µM
		Bcap-37	IC_50_ = 46.70 µM
**53**	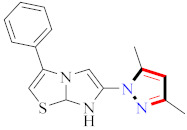	UO-31	% cell growth inhibition = 59.16
		SNB-75	% cell growth inhibition = 64.07
**54**	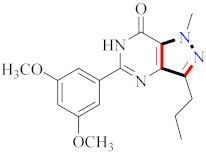	HeLa	IC_50_ = 19 µM
		CAKI-I	IC_50_ = 17 µM
		PC-3	IC_50_ = 37 µM
		MiaPaca-2	IC_50_ = 24 µM
		A549	IC_50_ = 14 µM
**55**	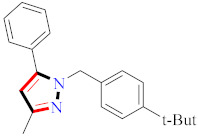	A549	IC_50_ = 2.56 µM
**56**	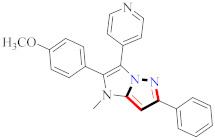	A549	IC_50_ = 2.0 µM
		B16F10	IC_50_ = 0.4 µM
		Hs683	IC_50_ = 1.3 µM
**57**	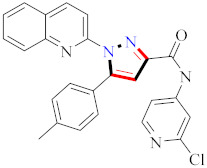	HCT-116	IC_50_ = 1.1 µM
		Huh7	IC_50_ = 1.6 µM
**58**	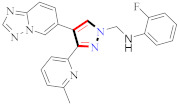	ALK5	IC_50_ = 13 nM
**59**	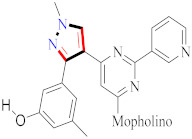	ROS1	IC_50_ = 13.6 nM
**60**	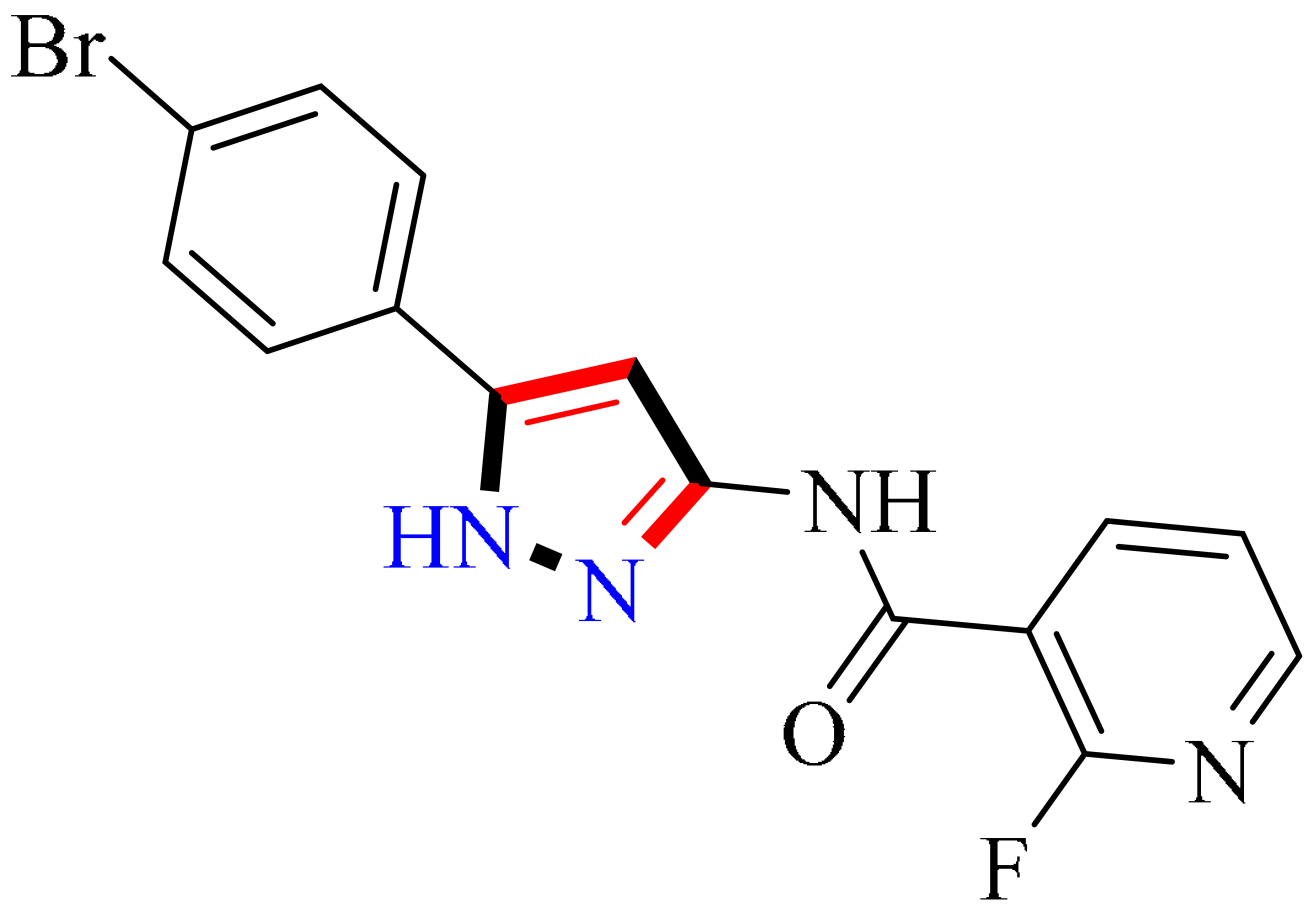	WM266.64	IC_50_ = 2.63 µM
		A375	IC_50_ = 3.16 µM
**61**	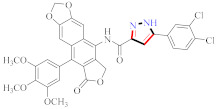	A549	IC_50_ = 31.07 µM
**62**	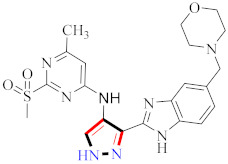	K562	IC_50_ = 5.003 µM
		HT29	IC_50_ = 0.381 µM
		LoVo	IC_50_ = 0.789 µM
		A549	IC_50_ = 0.487 µM
		U937	IC_50_ = 5.117 µM
**63**	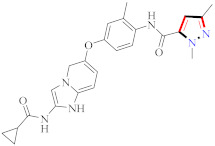	Aurora A/B kinase	IC_50_ = 28.9 µM
**64**	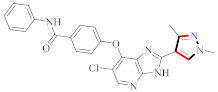	VEGFR2	IC_50_ = 0.95 nM
		Human umbilical vein endothelial cells	IC_50_ = 0.30 nM

**Table 6:** Representative compound structures **65** to **81** and their anticancer activity.

Compounds	Structure	Anticancer Activity	
		Cancer Cell Line	Biological Activity Value
**65**	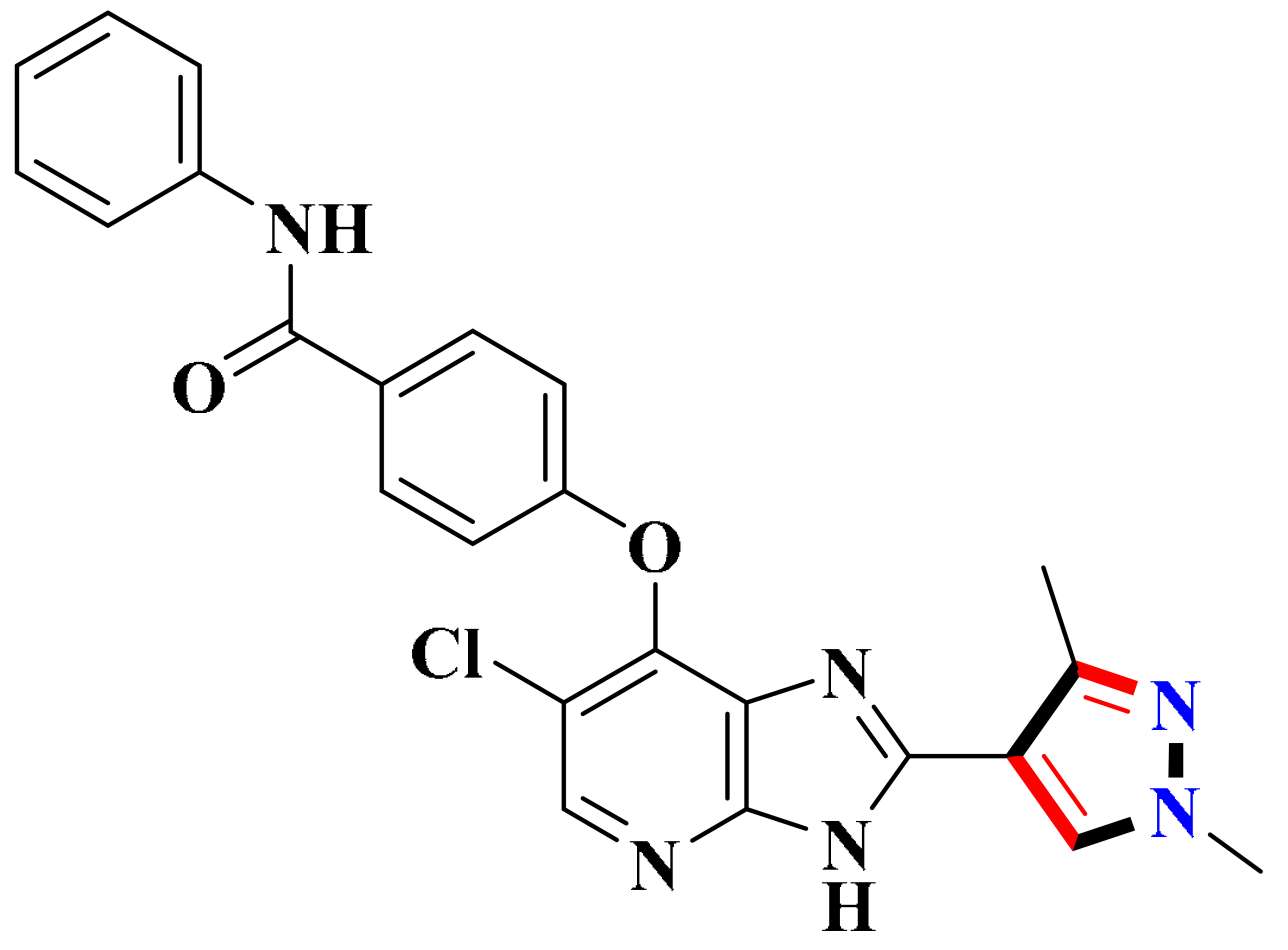	Aurora-A kinase	IC_50_ = 0.067 µM
**66**	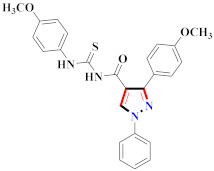	CDK2	IC_50_ = 25 nM
		H460	IC_50_ = 0.75–4.21 µM
**67**	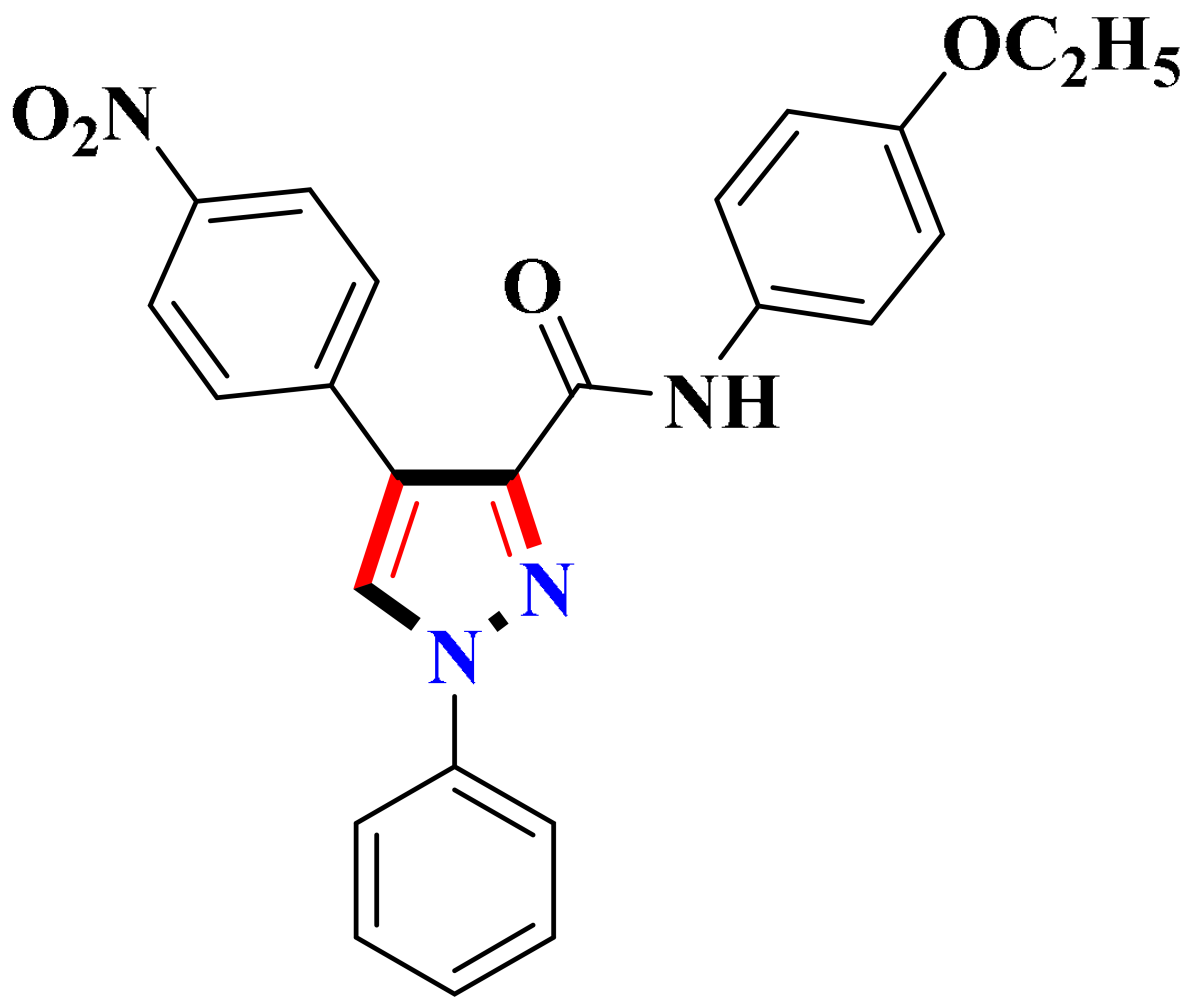	HCT116	IC_50_ = 0.39 µM
		MCF-7	IC_50_ = 0.46 µM
		Aurora-A kinase	IC_50_ = 00.16 µM
**68**	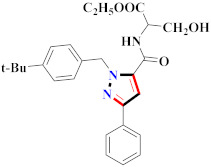	A549	IC_50_ = 36.12 µM
**69**	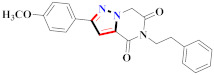	H322	IC_50_ = 280.5 µM
**70**	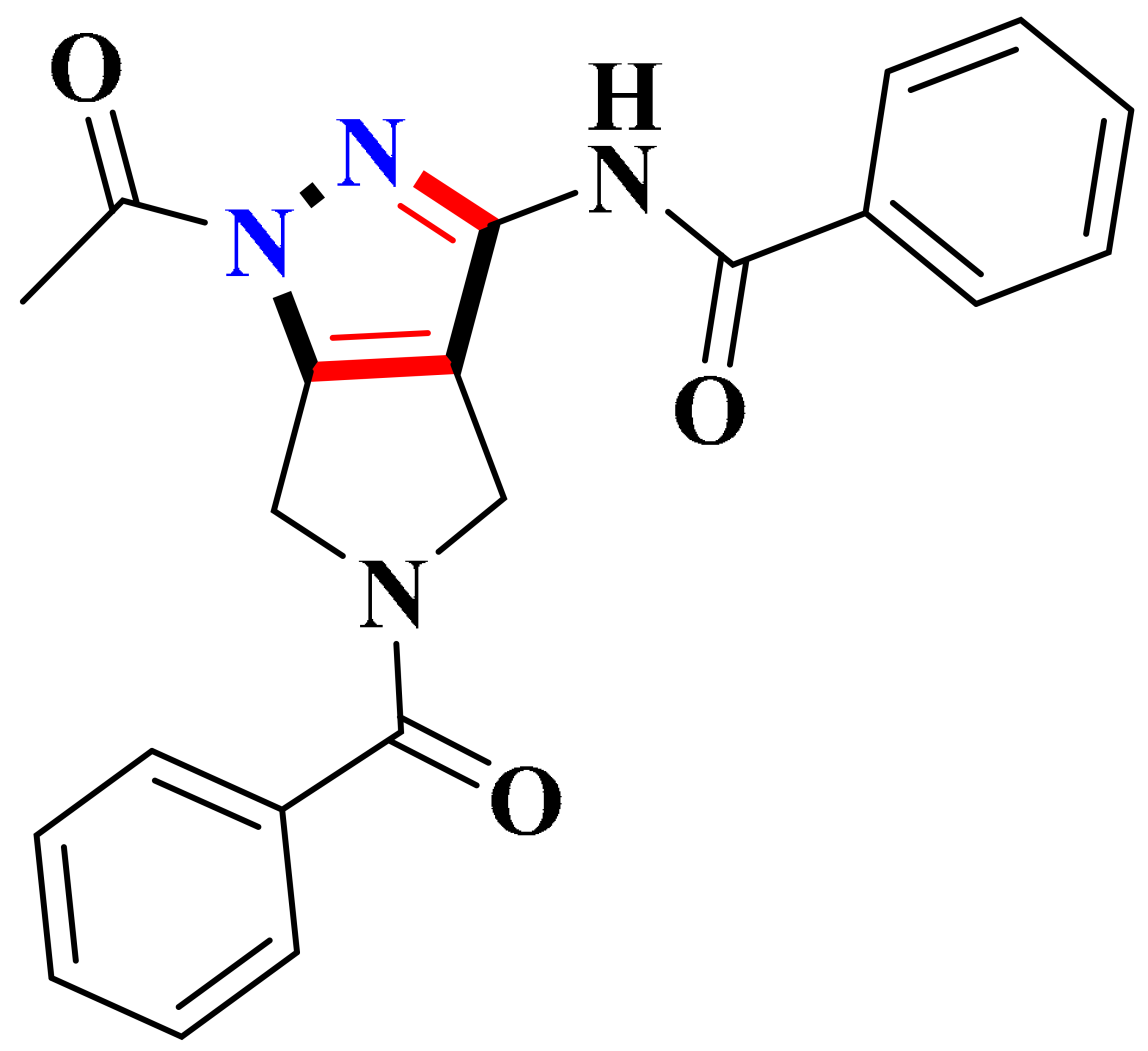	HCT-116	IC_50_ = 0.58 µM
**71**	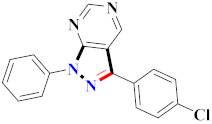	NCI-H226	IC_50_ = 45 µM
		NPC-TW01	IC_50_ = 59 µM
**72**	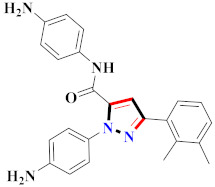	HCC	IC_50_ = 50 µM
		SNU449	IC_50_ = 100 µM
**73**	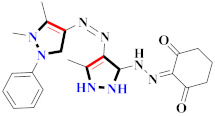	HepG2	IC_50_ = 8.3 µg/mL
**74**	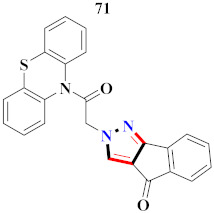	HCT-116,	GI% = 55.7 µM
		SKMEL-5	GI% = 51.4 µM
		LOX IMVI	GI% = 54.8 µM
**75**	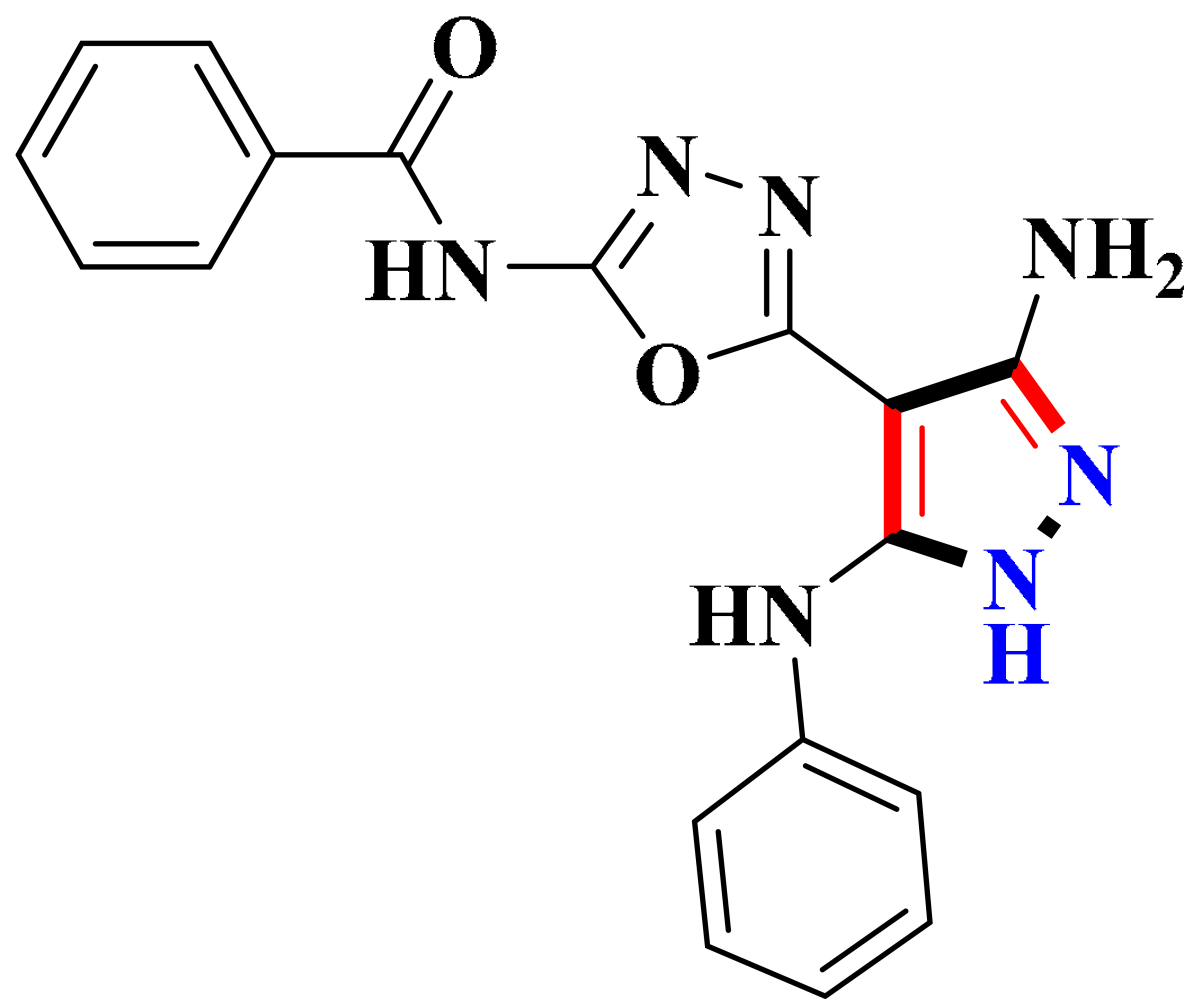	-	-
**76**	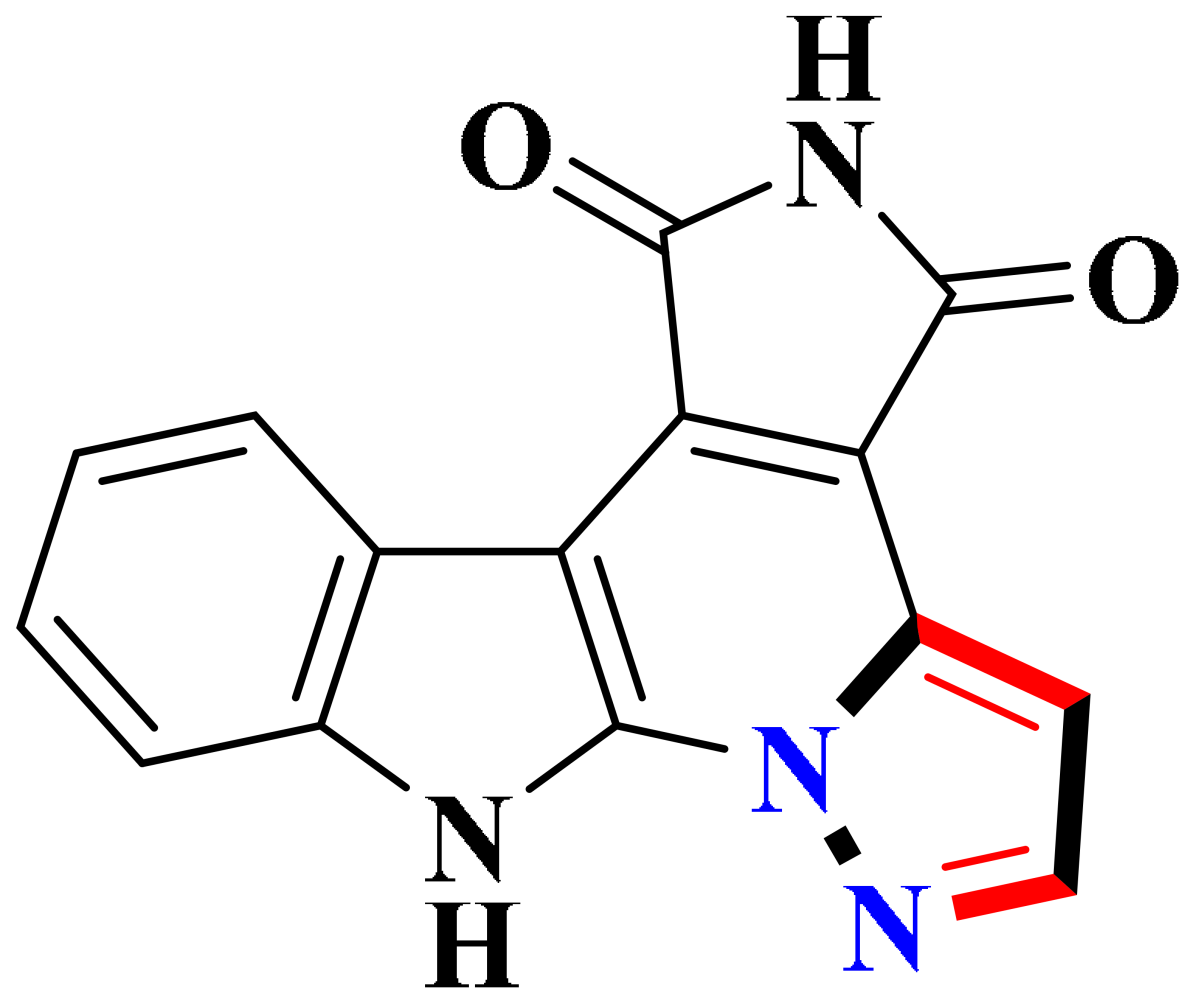	anticancer activity	IC_50_ = 11 µM
**77**	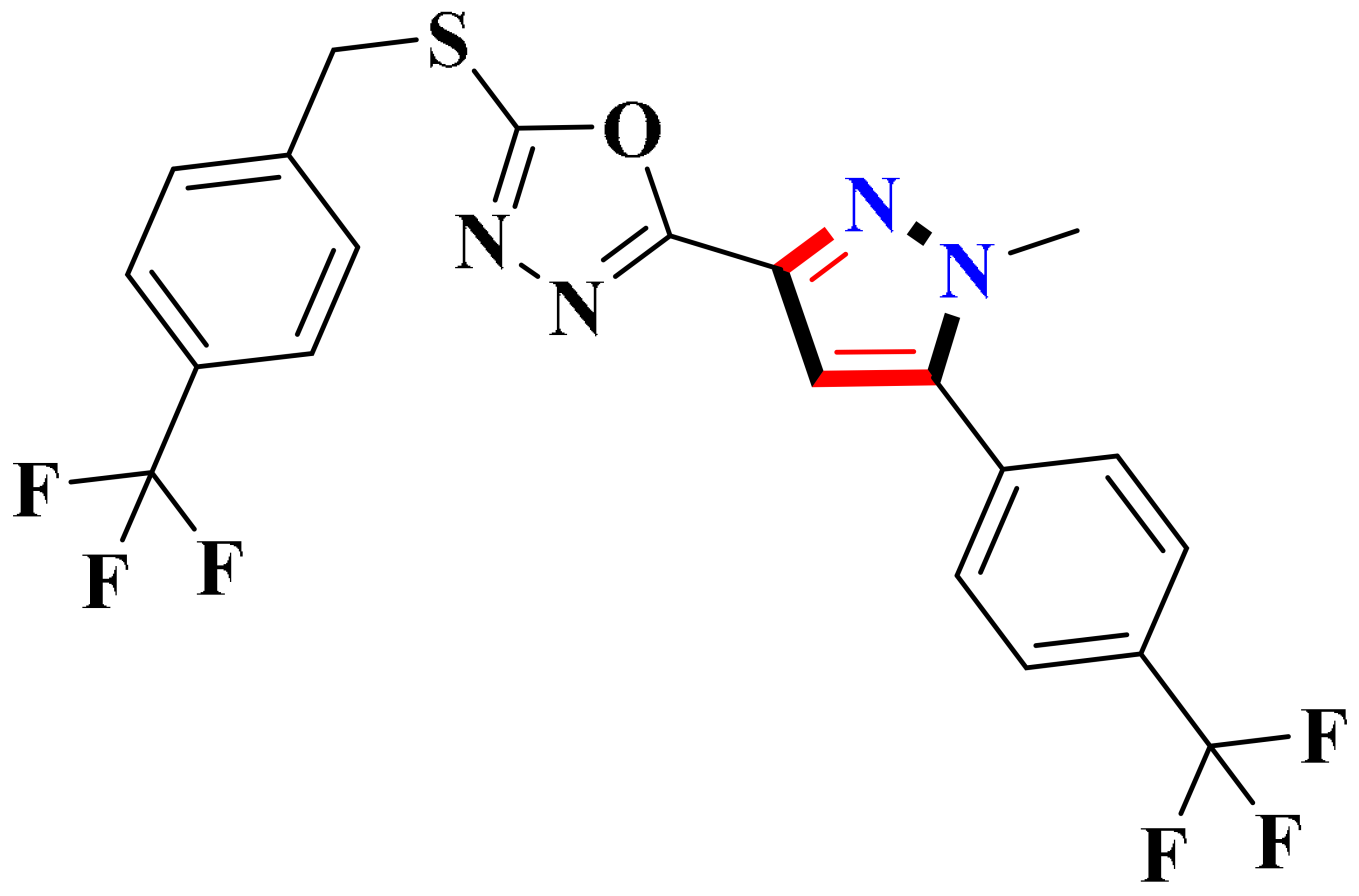	MCF-7	IC_50_ = 15.54 µM
**78**	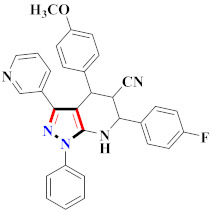	-	-
**79**	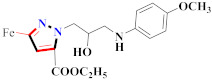	A549	IC_50_ = 28.3 5.7 µM
**80**	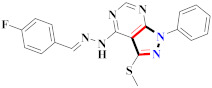	MCF-7	IC_50_ = 7.5 nM
**81**	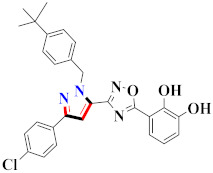	A549	IC_50_ = 1.54 µM
		NCIH23	IC_50_ = 0.36 µM

**Table 7:** Representative compound structures **82** to **99** and their anticancer activity.

Compounds	Structure	Anticancer Activity	
		Cancer Cell Line	IC50 (µM)
**82**	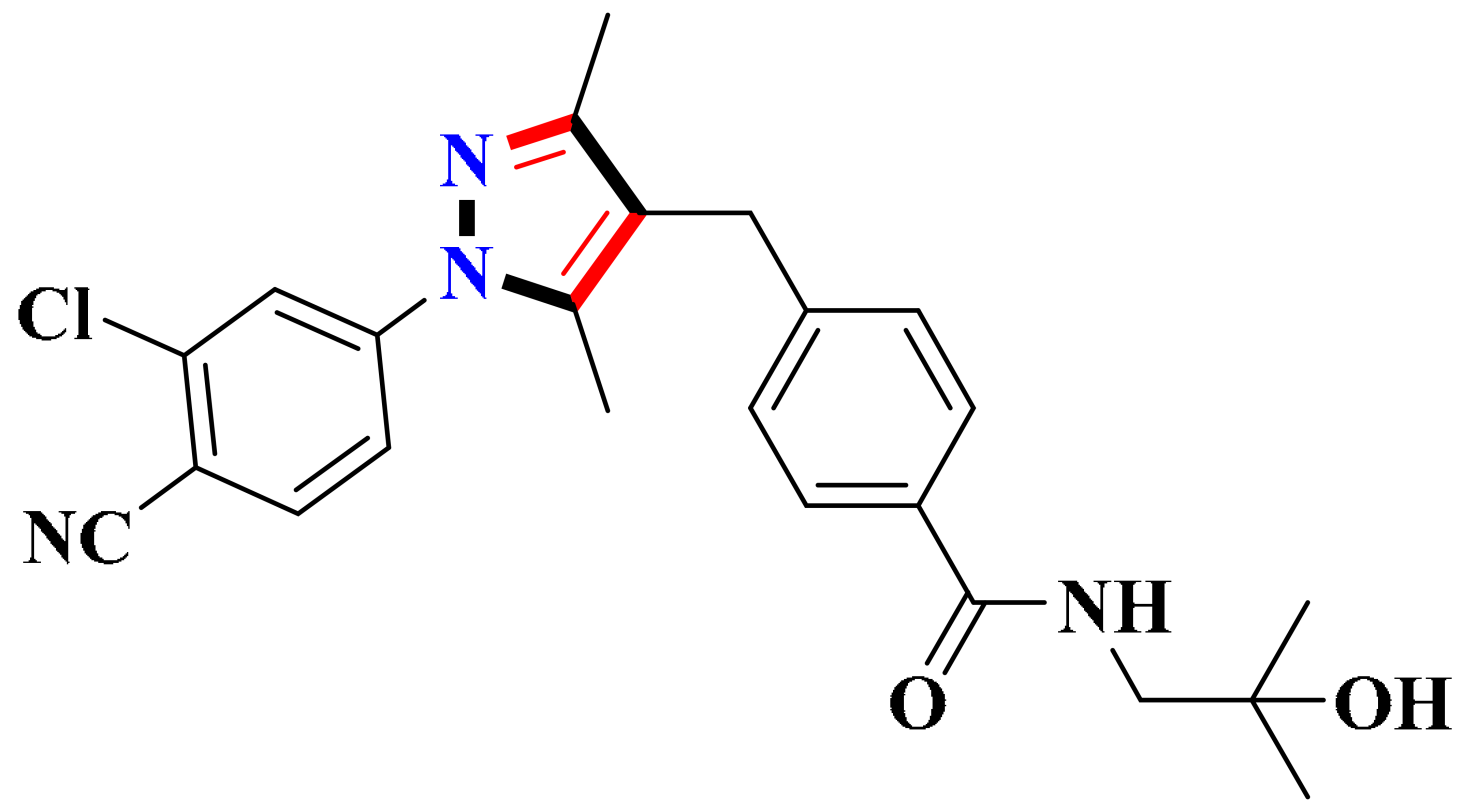	CRPC model of LNCap-hr	IC_50_ = 27 µM
**83**	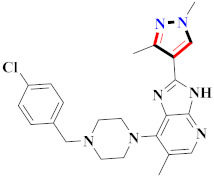	-	-
**84**	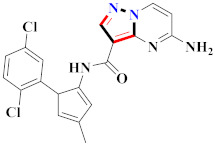	Janus Kinase	IC_50_ = 7.4 nM
**85**	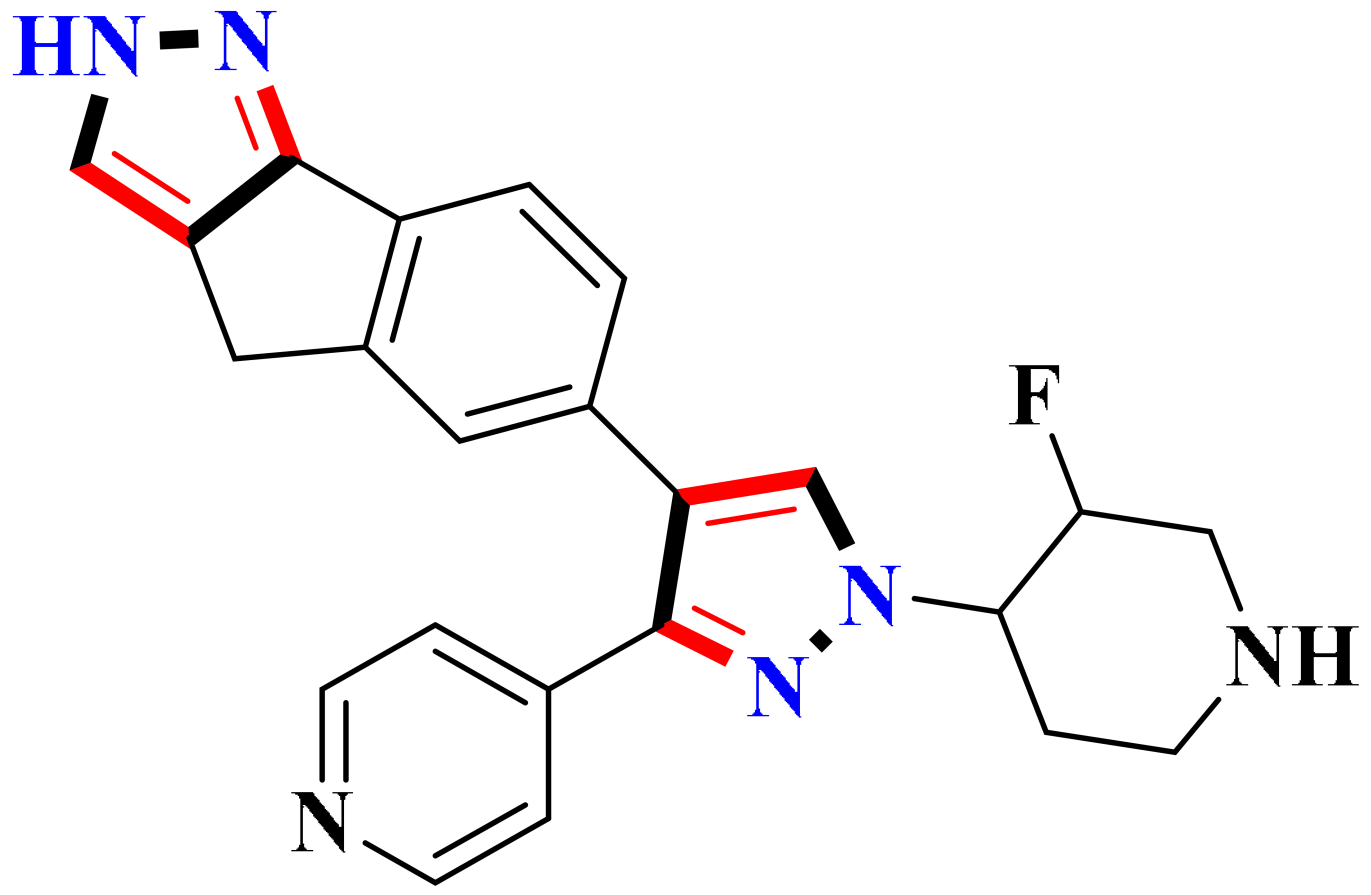	B-Raf	IC_50_ = 1.4 nM
**86**	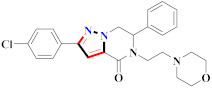	H322	IC_50_ = 29.4 µM
		A549	IC_50_ = 24.2 µM
**87**	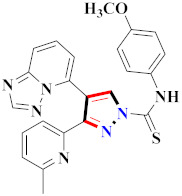	ALK5	IC_50_ = 0.57 nM
**88**	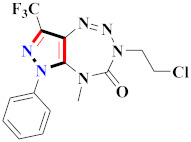	HL60K562	IC_50_ = 2.54 µM IC_50_ = 3.60 µM
**89a**	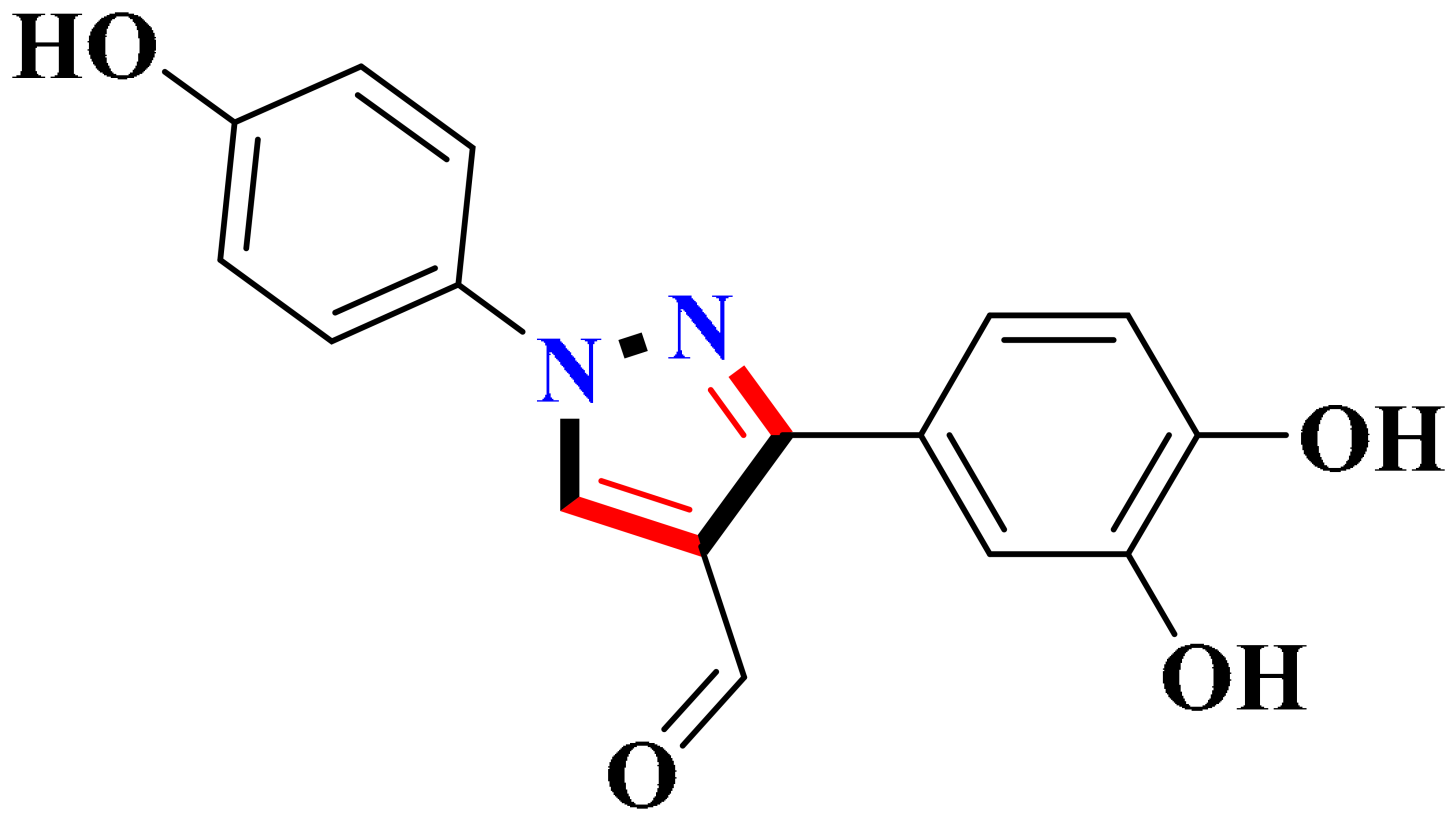	MCF7	IC_50_ = 12 µM
		HeLa	IC_50_ = 26 µM
**89b**	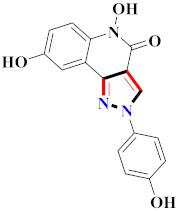	MCF7	IC_50_ = 25 µM
		HeLa	IC_50_ = 37 µM
**90**	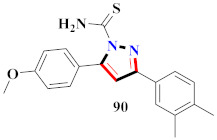	EGFR	IC_50_ = 0.07 µM
**91**	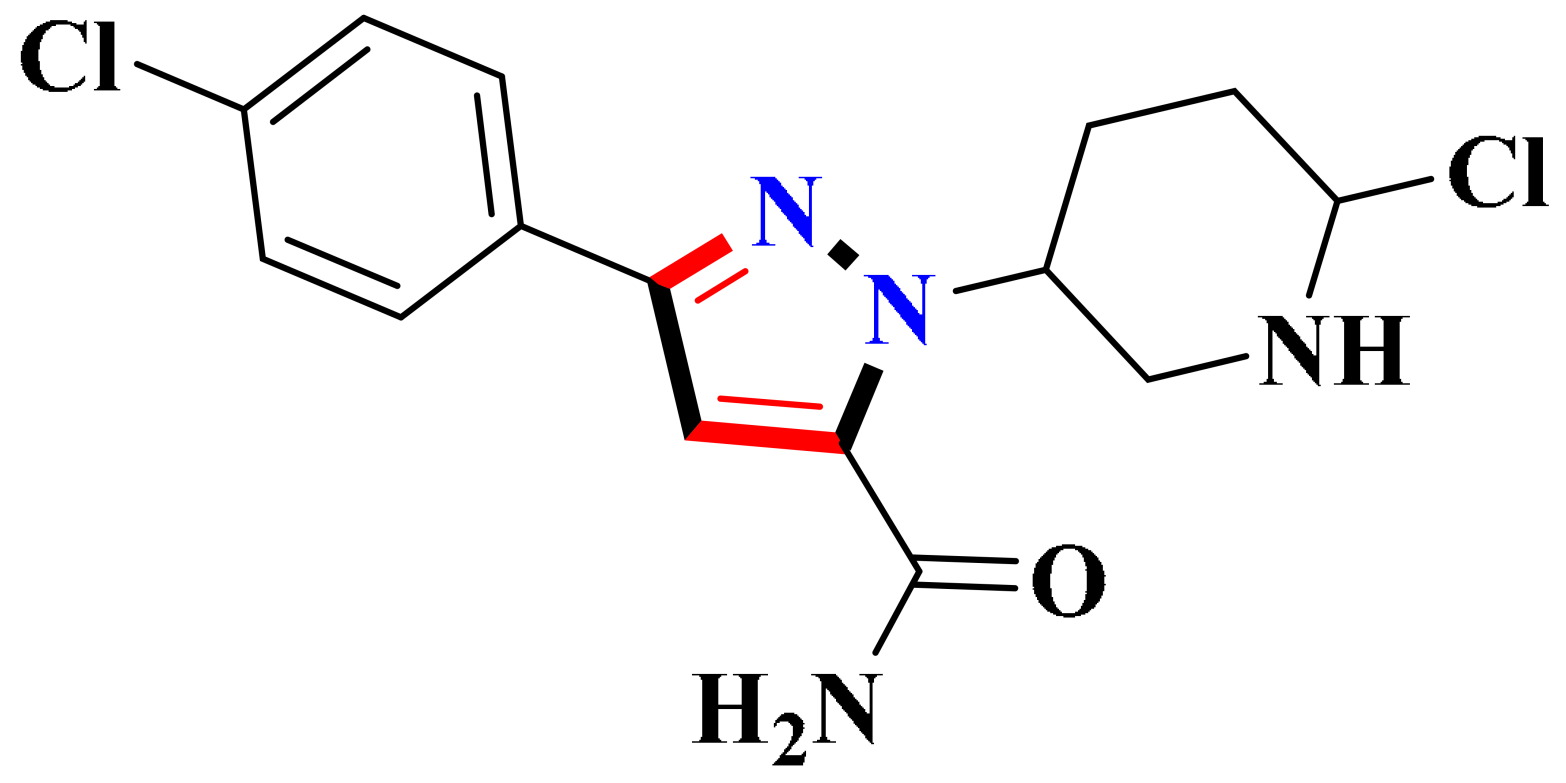	A549	-
**92**	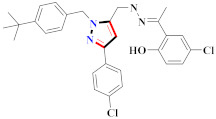	A549	IC_50_ = 0.28 µM
**93a**	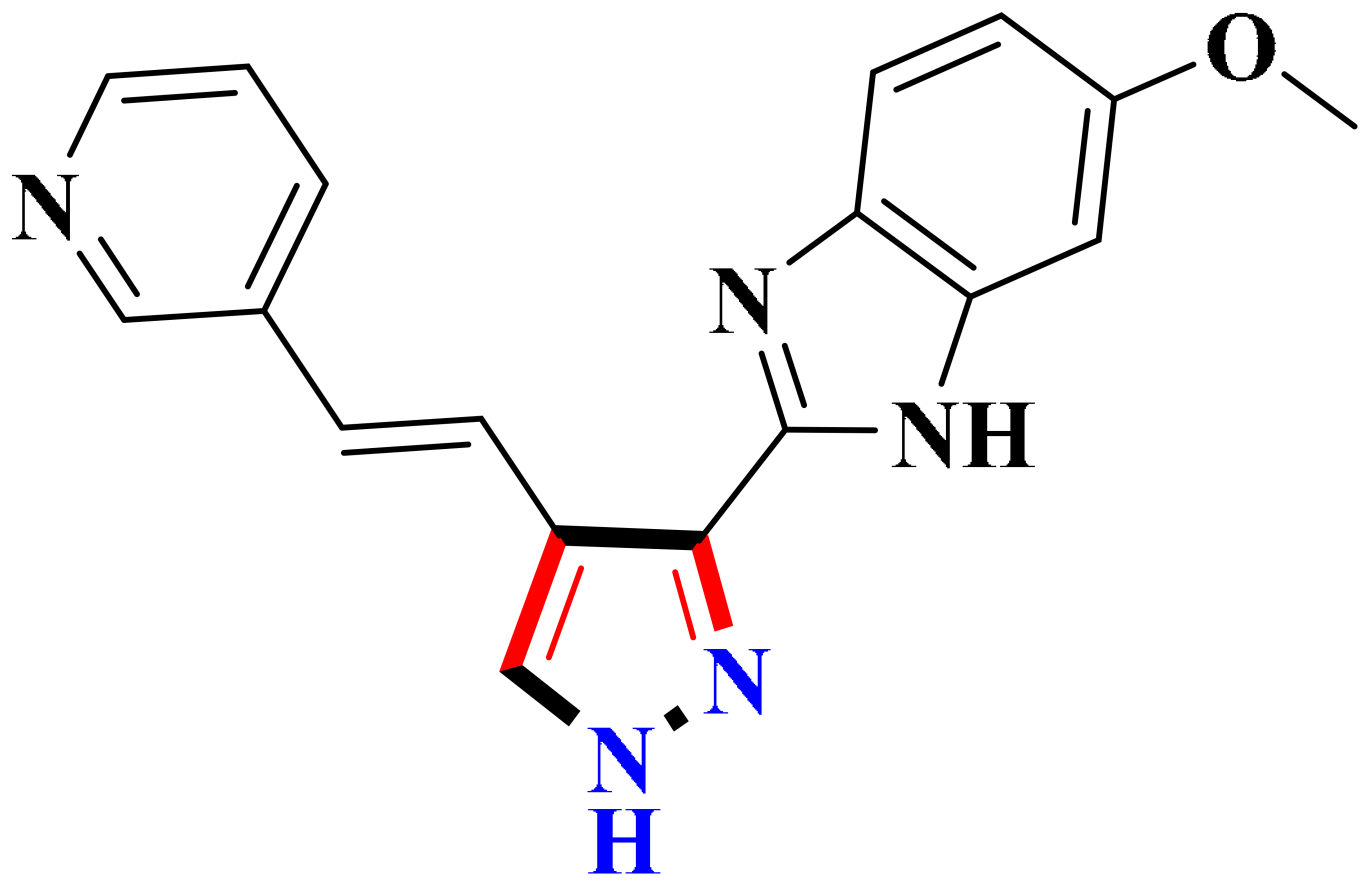	-	-
**93b**	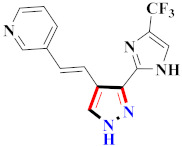	-	-
**94**	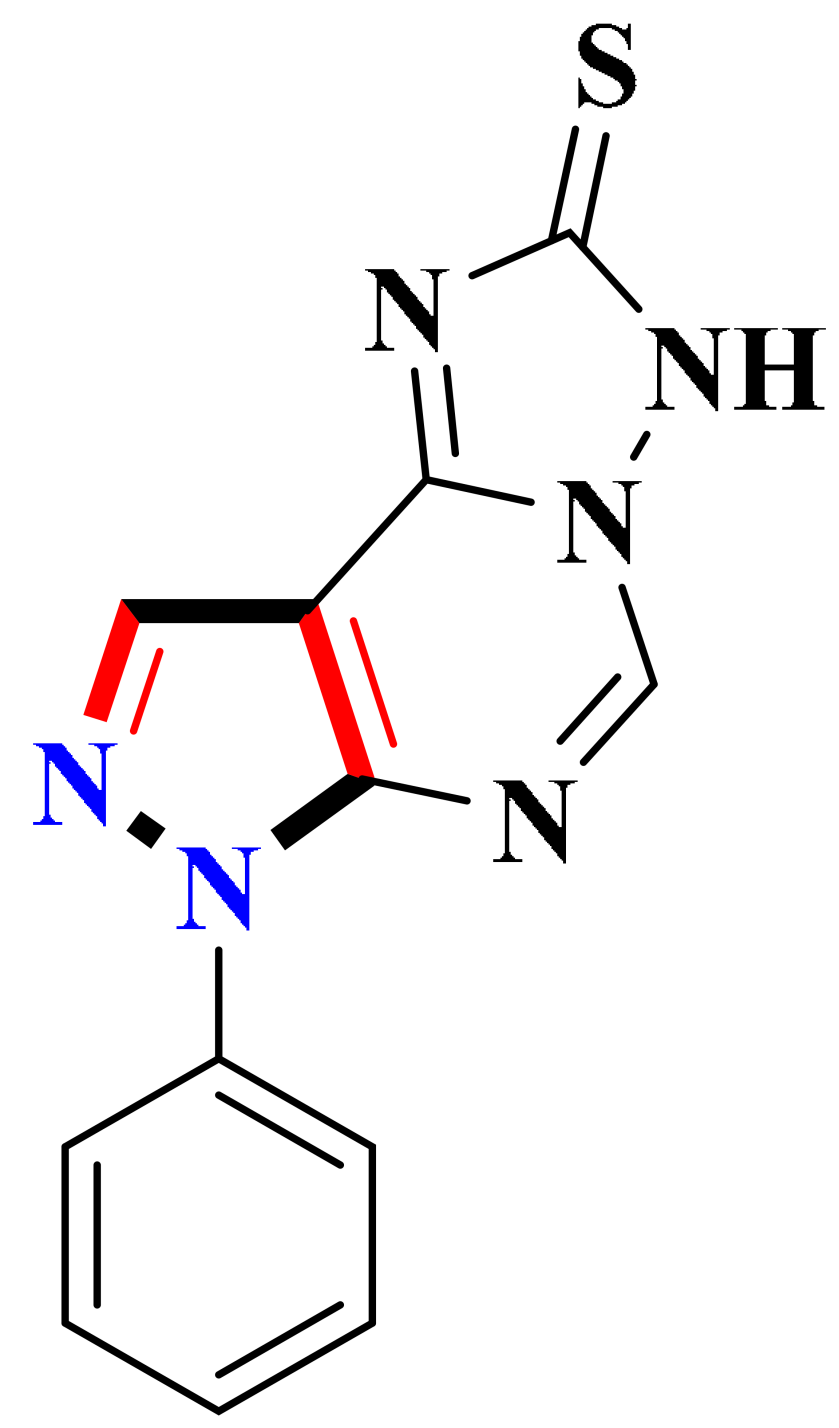	-	-
**95**	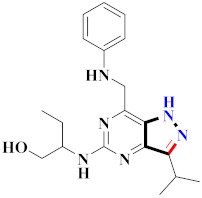	-	-
**96**	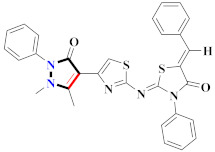	HepG2	IC_50_ = 13.17 µM
**97**	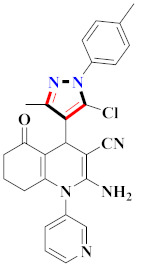	NCI-60	IC_50_ = 10 µM
**98**	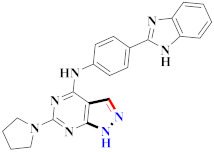	-	-
**99**	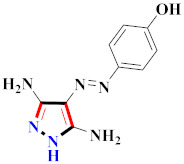	A2780	IC_50_ = 1.22 µM
		A549	IC_50_ = 52.5 µM
		P388	IC_50_ = 1.56 µM

**Table 8:** Representative compound structures **100** to **112** and their anticancer activity.

Compounds	Structure	Anticancer Activity	
		Cancer Cell Line	Biological Activity Value
**100**	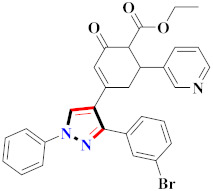	MCF-7	IC_50_ = 14.31 µM
		NCI-H460	IC_50_ = 8.55 µM
		HeLa	IC_50_ = 7.01 µM
**101**	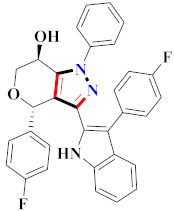	MCF-7	IC_50_ = 7.74 µM
		HGC-27	IC_50_ = >64 µM
		PC-3	IC_50_ = >64 µM
		EC-109	IC_50_ = 23.07 µM
**102**	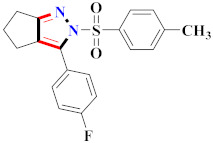	MCF-7	IC_50_ = 15.6 µM
		A589	IC_50_ = 19.8 µM
**103**	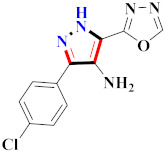	HT29	IC_50_ = 0.30 µM
**104**	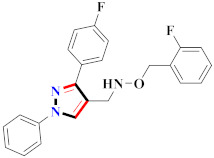	PI3K	IC_50_ = 0.28 µM
		lymph node cells	IC_50_ = 1.18 µM
**105**	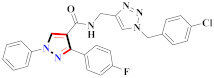	MIAPaCa-2	GI_50_ = 0.28 µM
		MCF-7	GI_50_ = 0.13 µM
		HeLa	GI_50_ = 0.21 µM
**106**	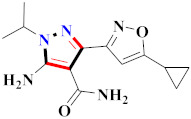	wild-type RET	IC_50_ = 44 nM
		gatekeeper mutant	IC_50_ = 252 nM
**107**	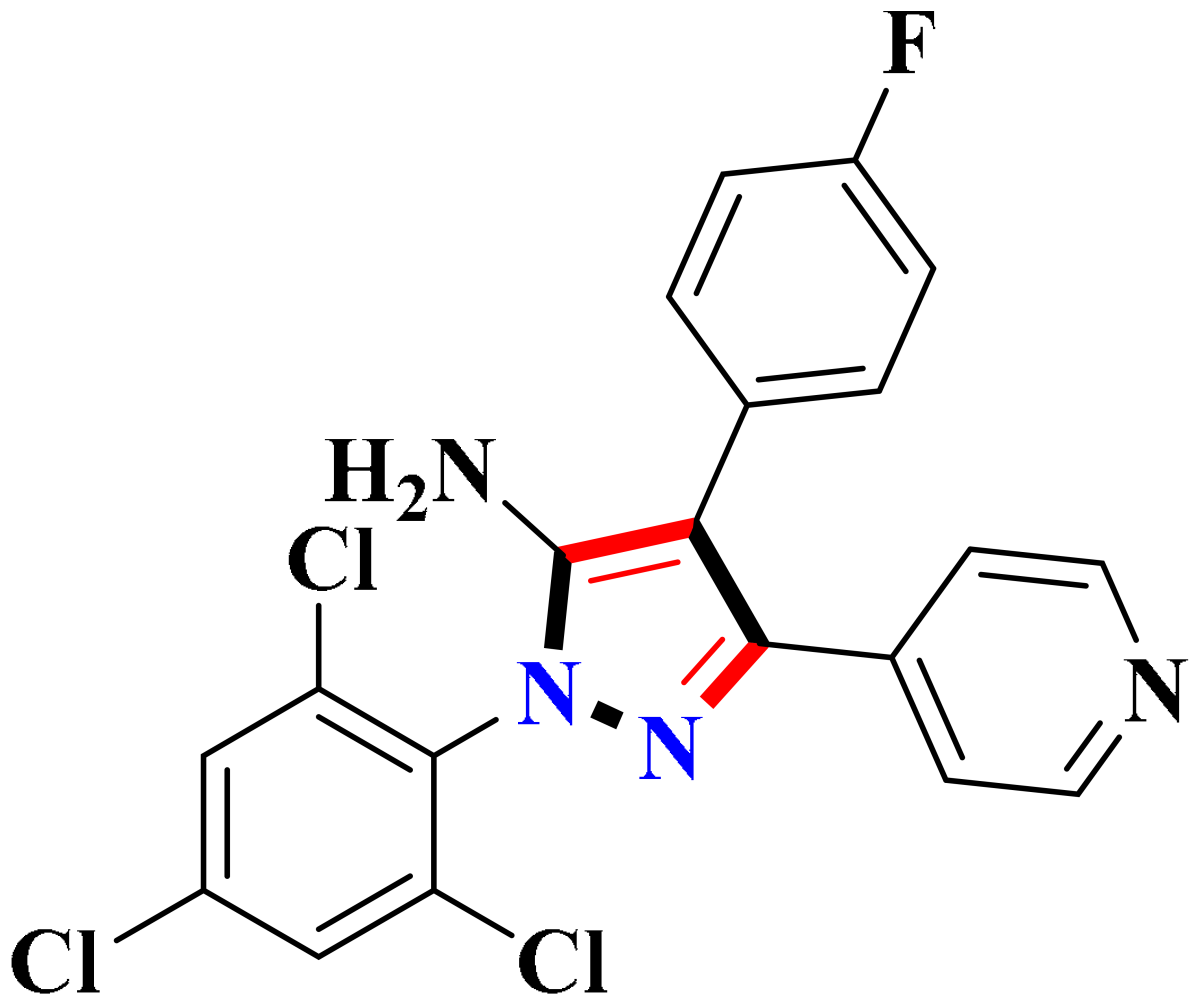	Raf V600E	IC_50_ = 0.59 µM
		Src	IC_50_ = 0.40 µM
		VEGFR-2	IC_50_ = 0.034 µM
**108**	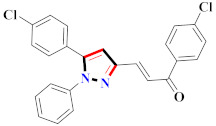	HCT-116	%GI = 42.4
**109**	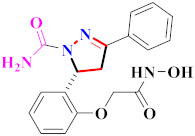	U93	IC_50_ = 54.2 µM
		PLC/PRF/5	IC_50_ = 116.5 µM
		K562	IC_50_ = 18.7 µM
		ES-2	IC_50_ = 156.0 µM
		PC-3	IC_50_ = 65.0 µM
		HepG2	IC_50_ = 65.0 µM
**110**	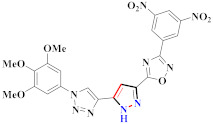	PC3	IC_50_ = 0.01 µM
		A549	IC_50_ = 0.45 µM
		MCF-7	IC_50_ = 0.081 µM
		DU-145	IC_50_ = 1.77 µM
**111**	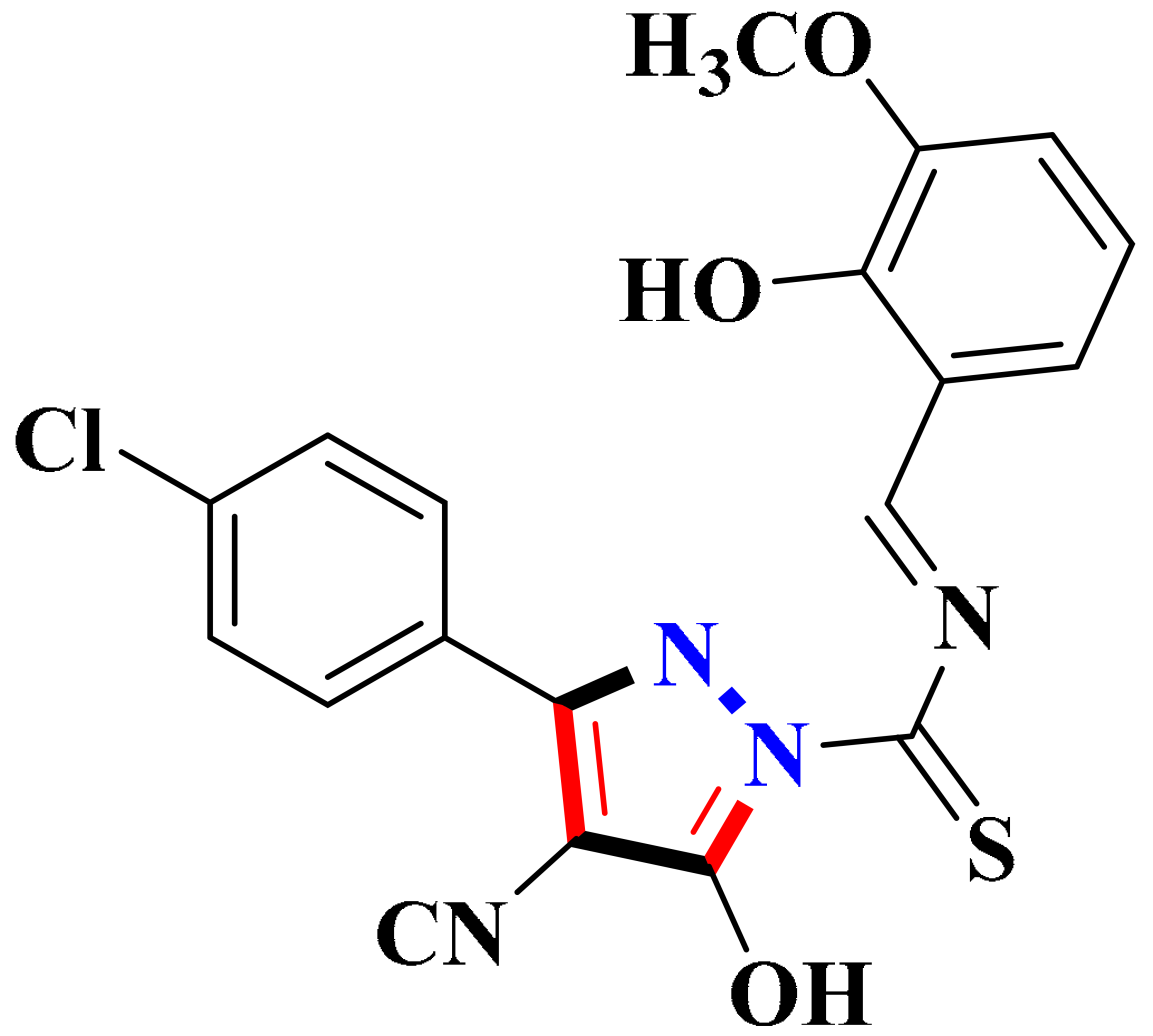	HL-60	IC_50_ = 56.04 nM
**112**	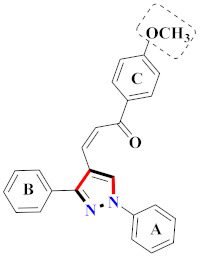	A549	IC_50_ = 4.94 µM
		SiHa	IC_50_ = 4.94 µM
		HepG2	IC_50_ = 2.09 µM
		COLO205	IC_50_ = 4.86 µM

**Table 9:** Representative compound structures **113** to **129** and their anti-inflammatory activity.

Compounds	Structure	Anti-Inflammatory Activity	
		Target Site	Biological Activity Value
**113**	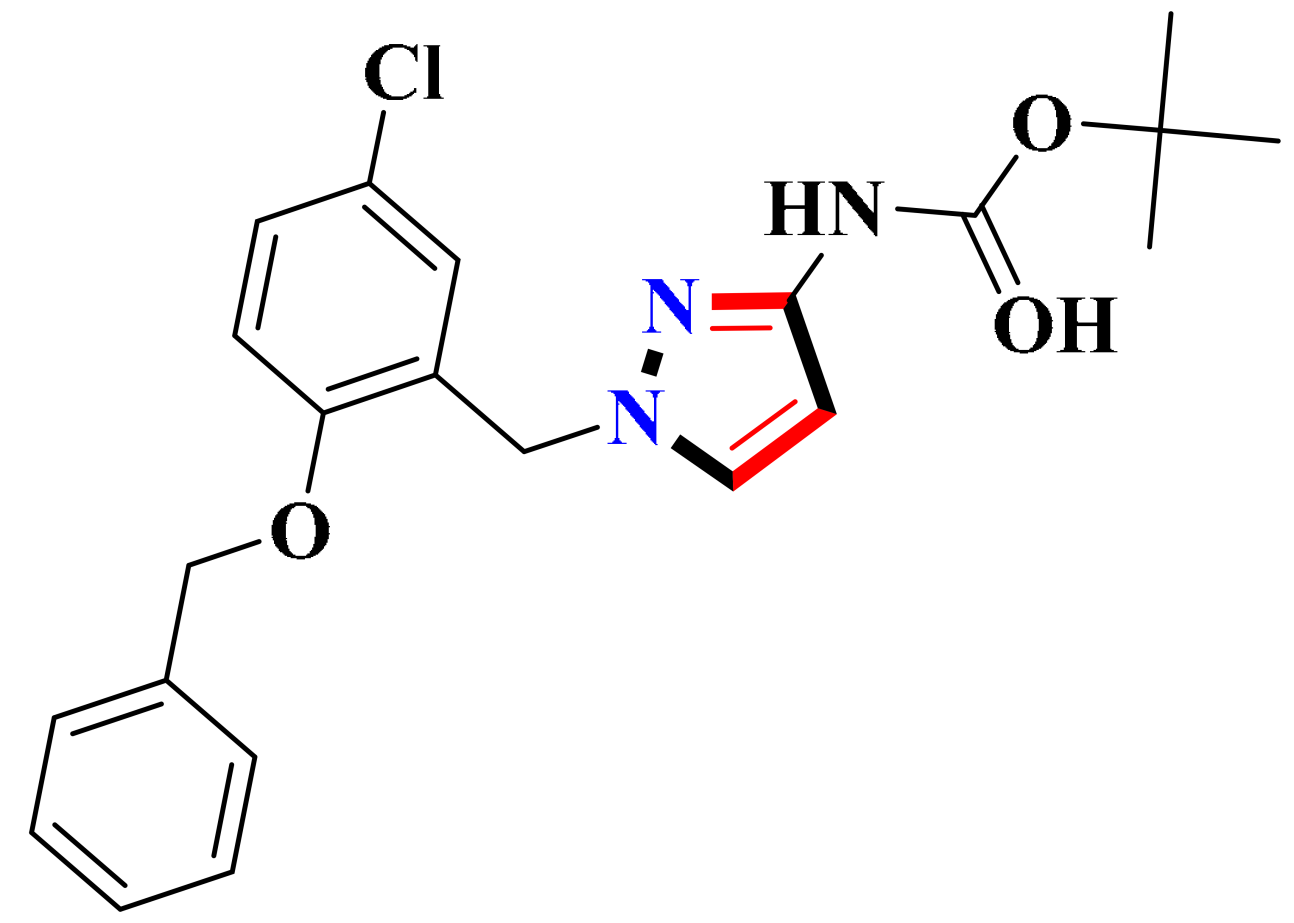	IL-6 inhibition	42% at 10 μM
**114**	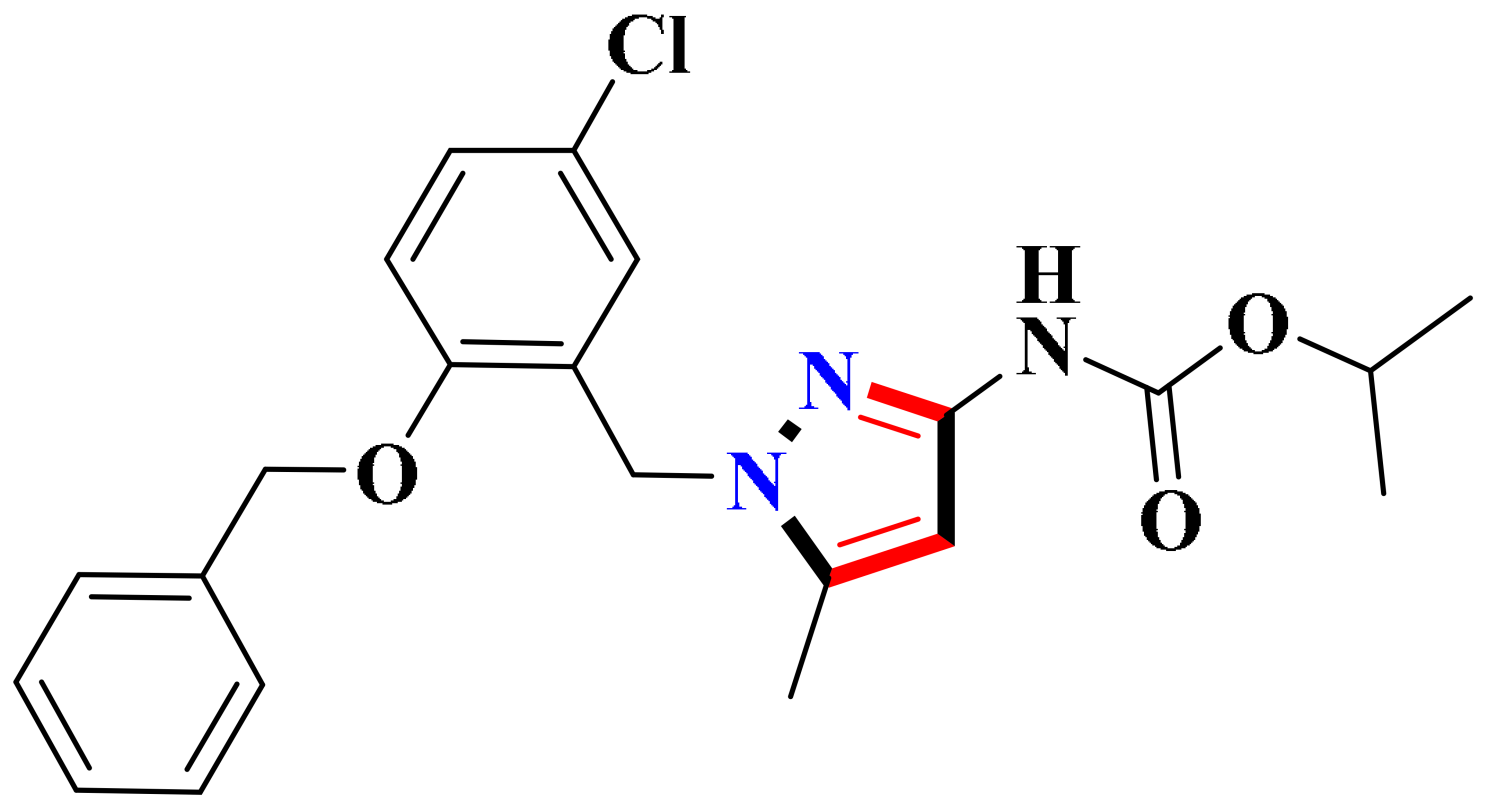	-	-
**115**	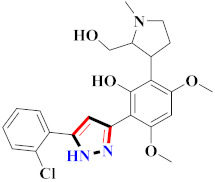	-	
**116**	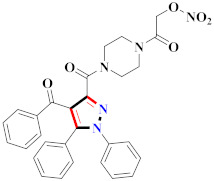	-	-
**117**	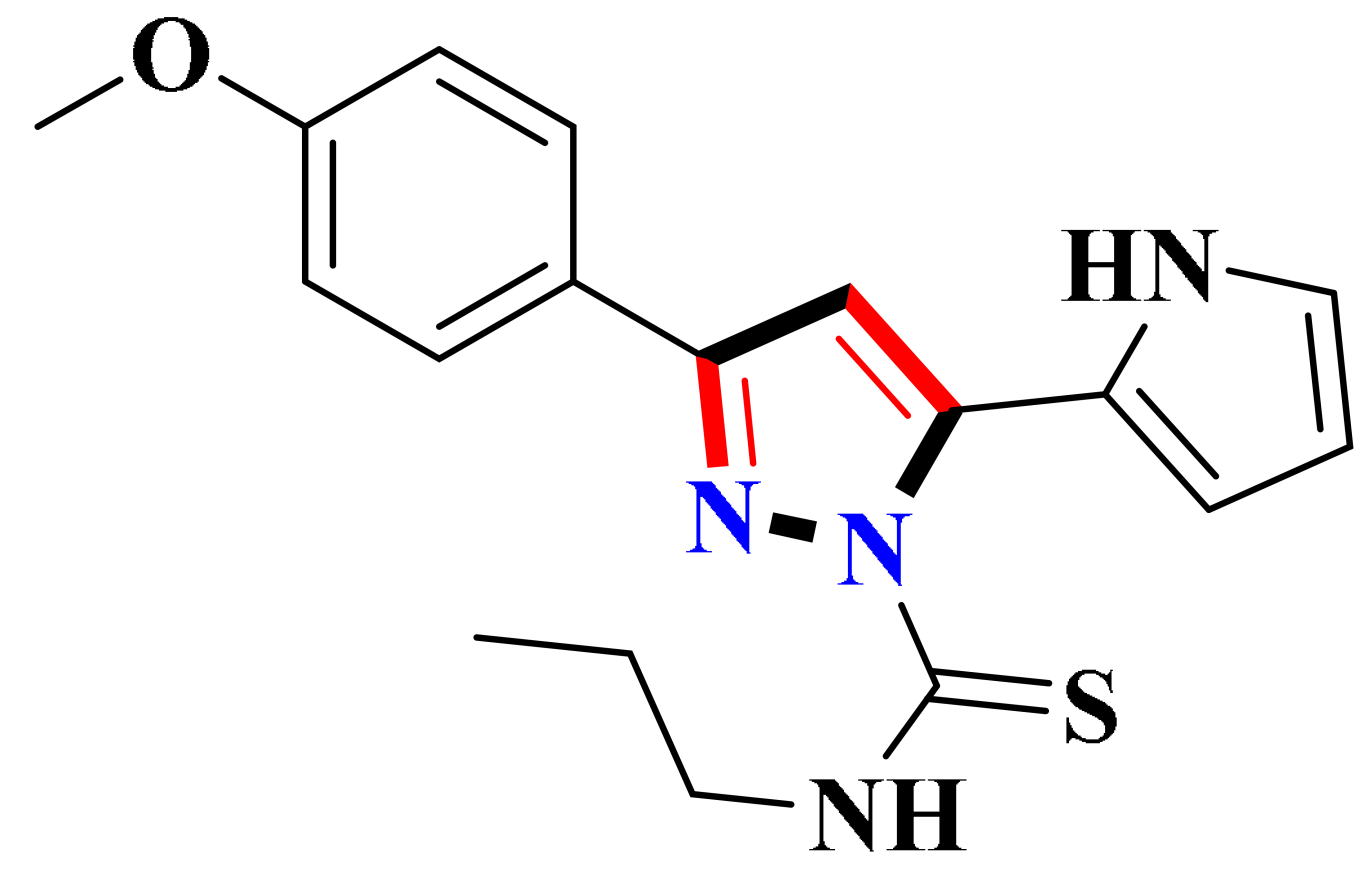	-	-
**118**	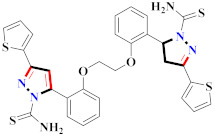	-	-
**119**	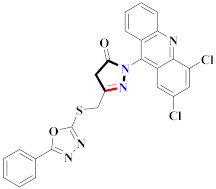	-	-
**120**	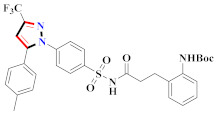	-	-
**121**	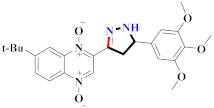	% Inhibition	56.1%
**122**	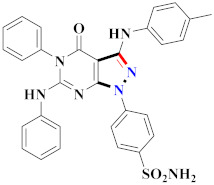	% Inhibition	60.24%
**123**	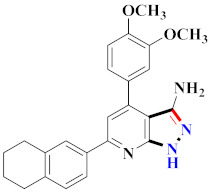	COX-2	IC_50_ = 15.70 µM
**124**	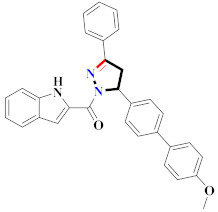	Lipoxygenase activity	IC_50_ = 48 µM
**125**	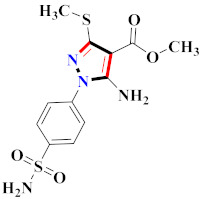	-	-
**126**	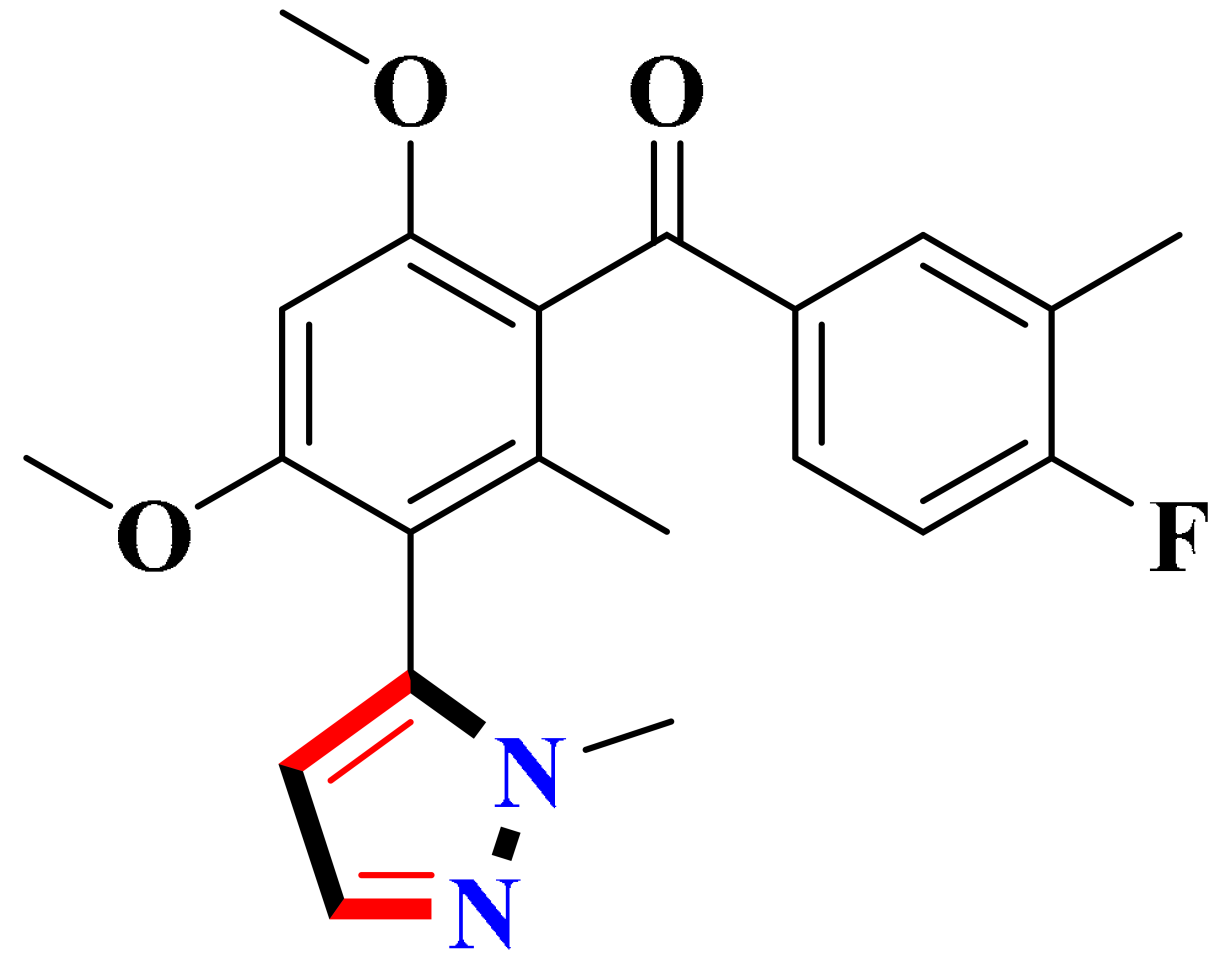	-	-
**127**	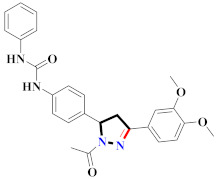	-	-
**128**	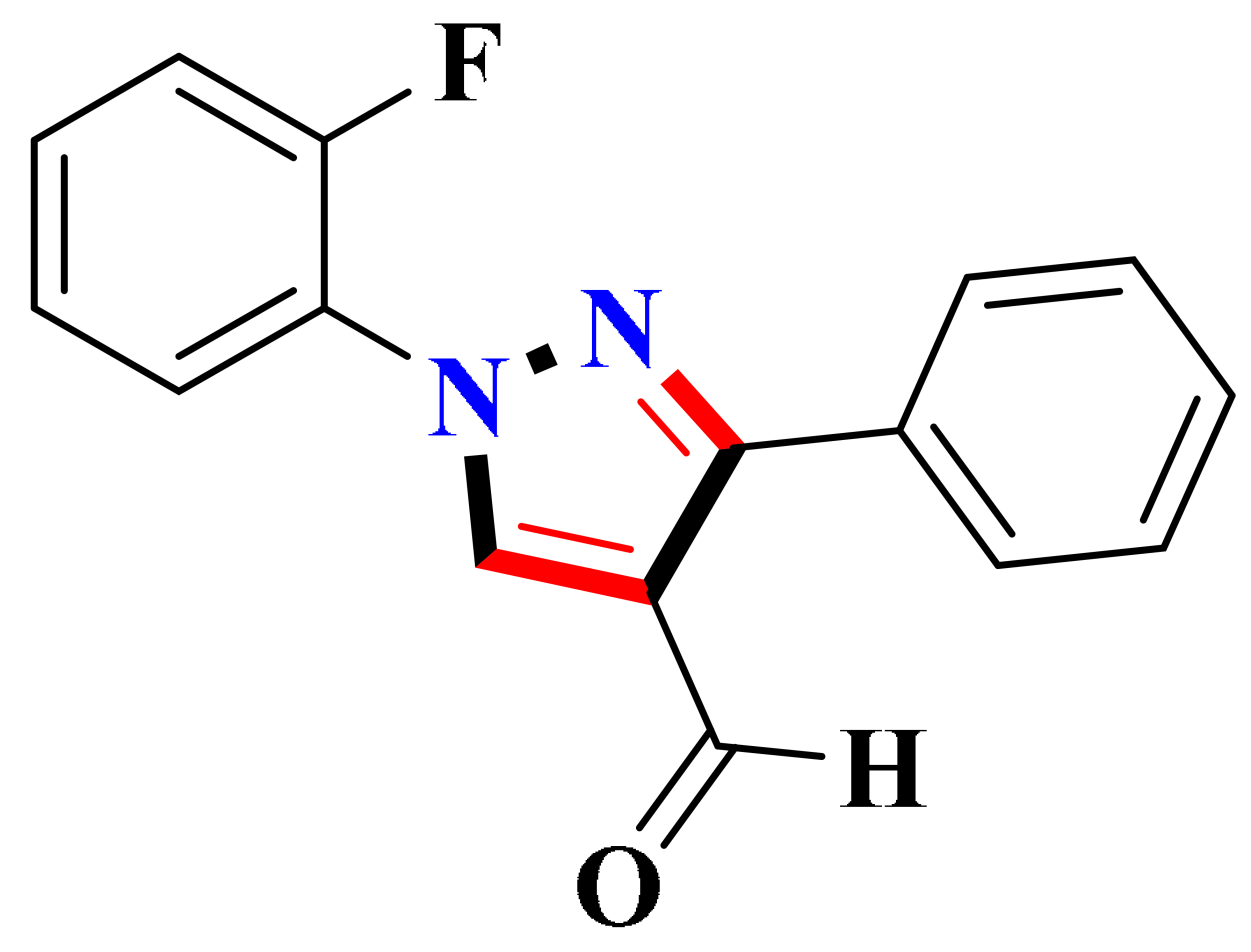	Percentage protection in paw edema	14%
**129**	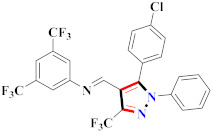	COX-2 inhibition	IC_50_ = 0.26 µM

**Table 10:** Representative compound structures **130** to **146** and their anti-inflammatory activity.

Compounds	Structure	Anti-Inflammatory Activity	
		Target Site	Biological Activity Value
**130**	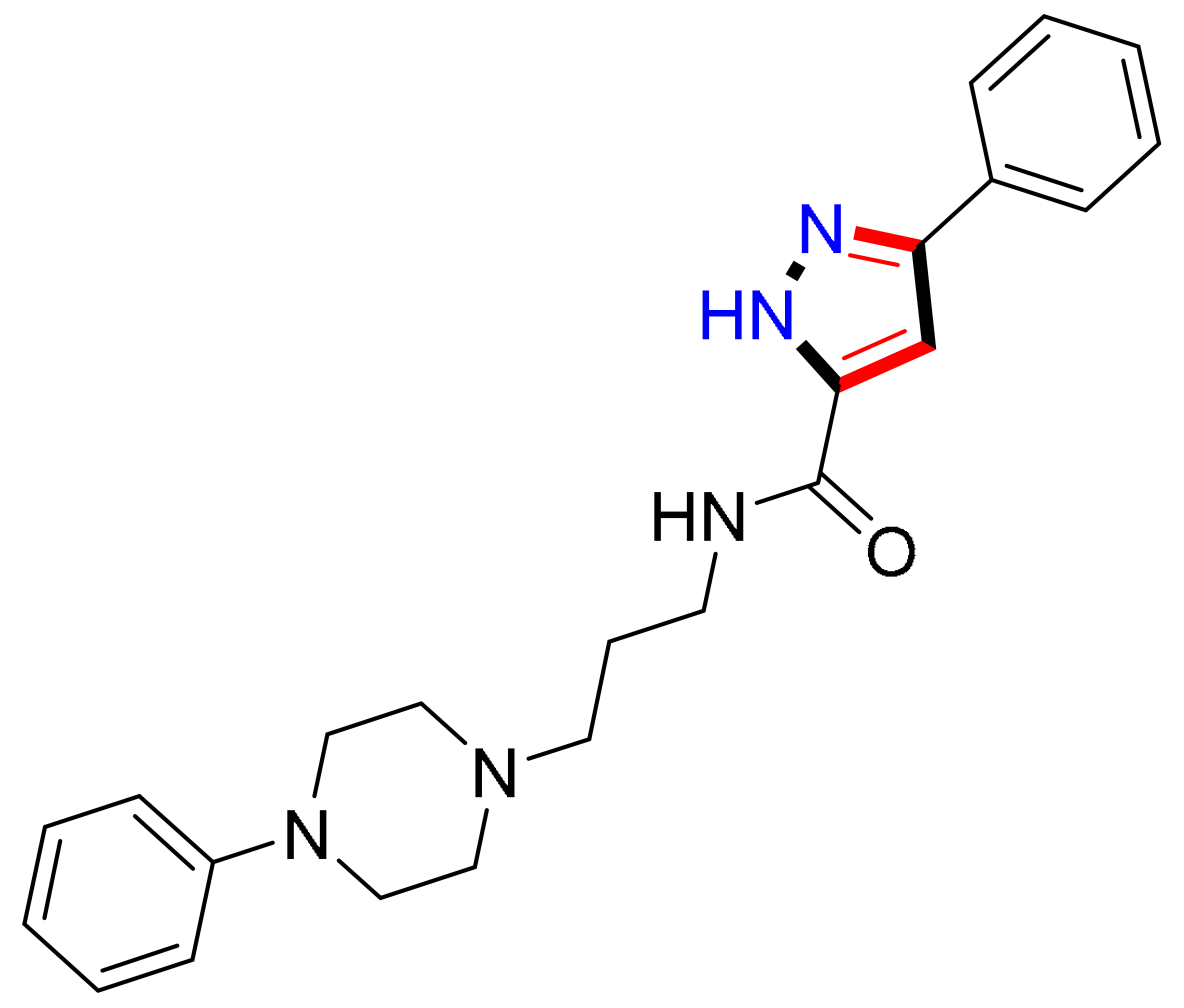	Increase in paw volume (mL)	0.20 mL
**131**	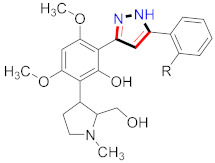	-	-
**132**	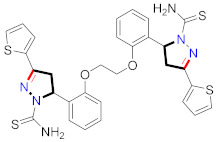	-	-
**133**	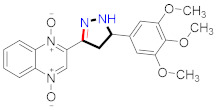	-	-
**134**	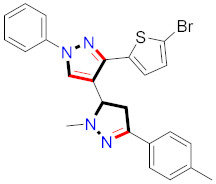	-	-
**135a**	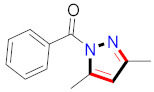	-	-
**135b**	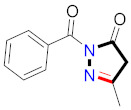	-	-
**136**	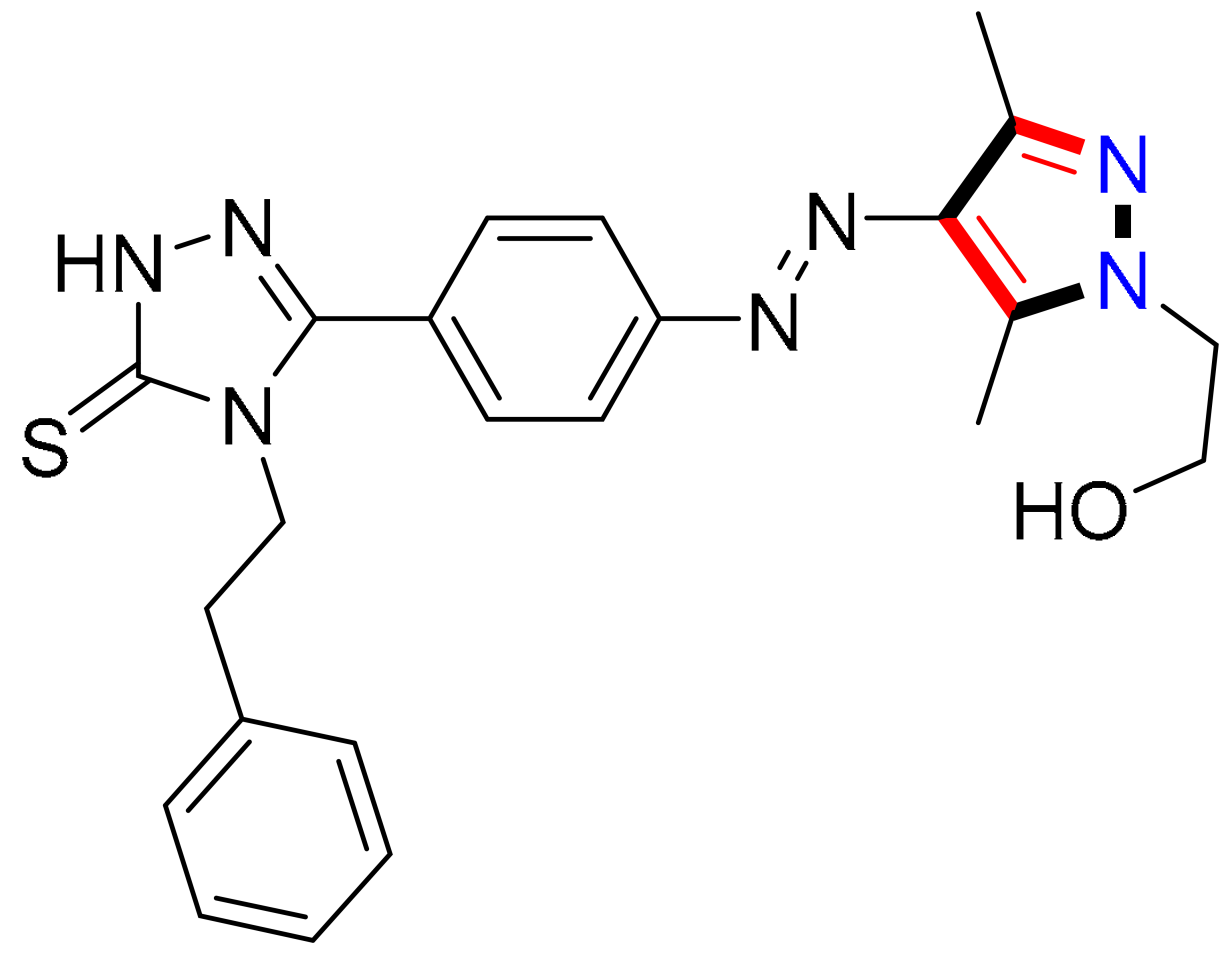	-	-
**137**	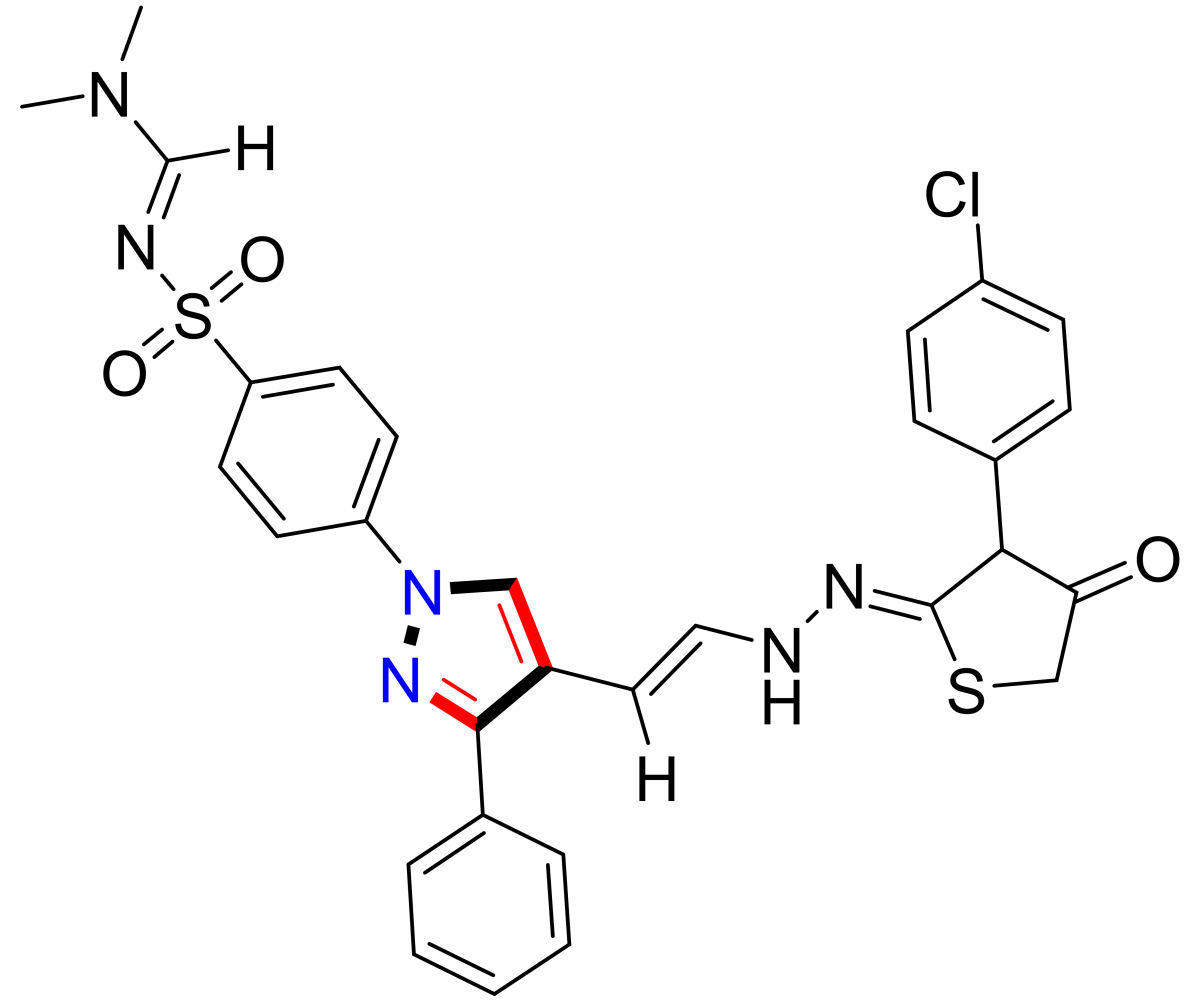	-	-
**138**	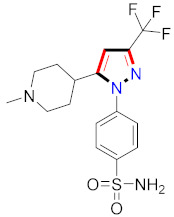	-	-
**139**	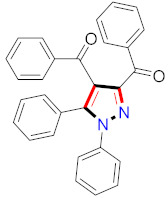	-	-
**140**	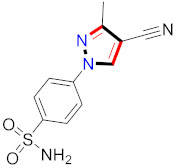	-	-
**141**	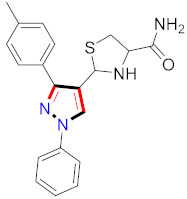	-	-
**142**	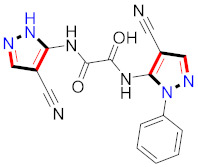	COX-1	IC_50_ = 11.2 µM
		COX-2	IC_50_ = 14.0 µM
**143**	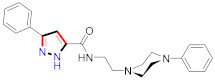	Increase in paw volume in mL	0.20 mL
**144**	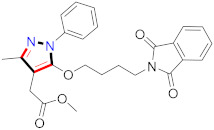	-	-
**145**	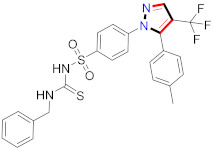	percent inhibitory activity	72.0%
**146**	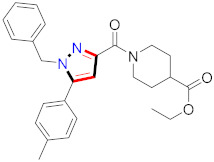	-	-

**Table 11:** Representative compound structures **147** to **161** and their anti-inflammatory activity.

Compounds	Structure	Anti-Inflammatory Activity	
		Target Site	Biological Activity Value
**147**	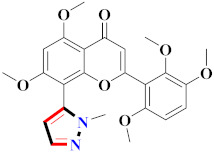	COX-1	IC_50_ = 45.67 µM
**148**	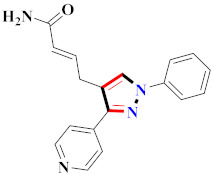	-	-
**149**	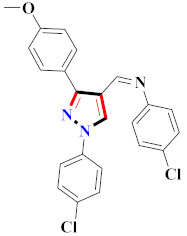	-	-
**150**	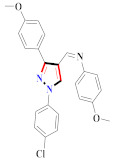	-	-
**151**	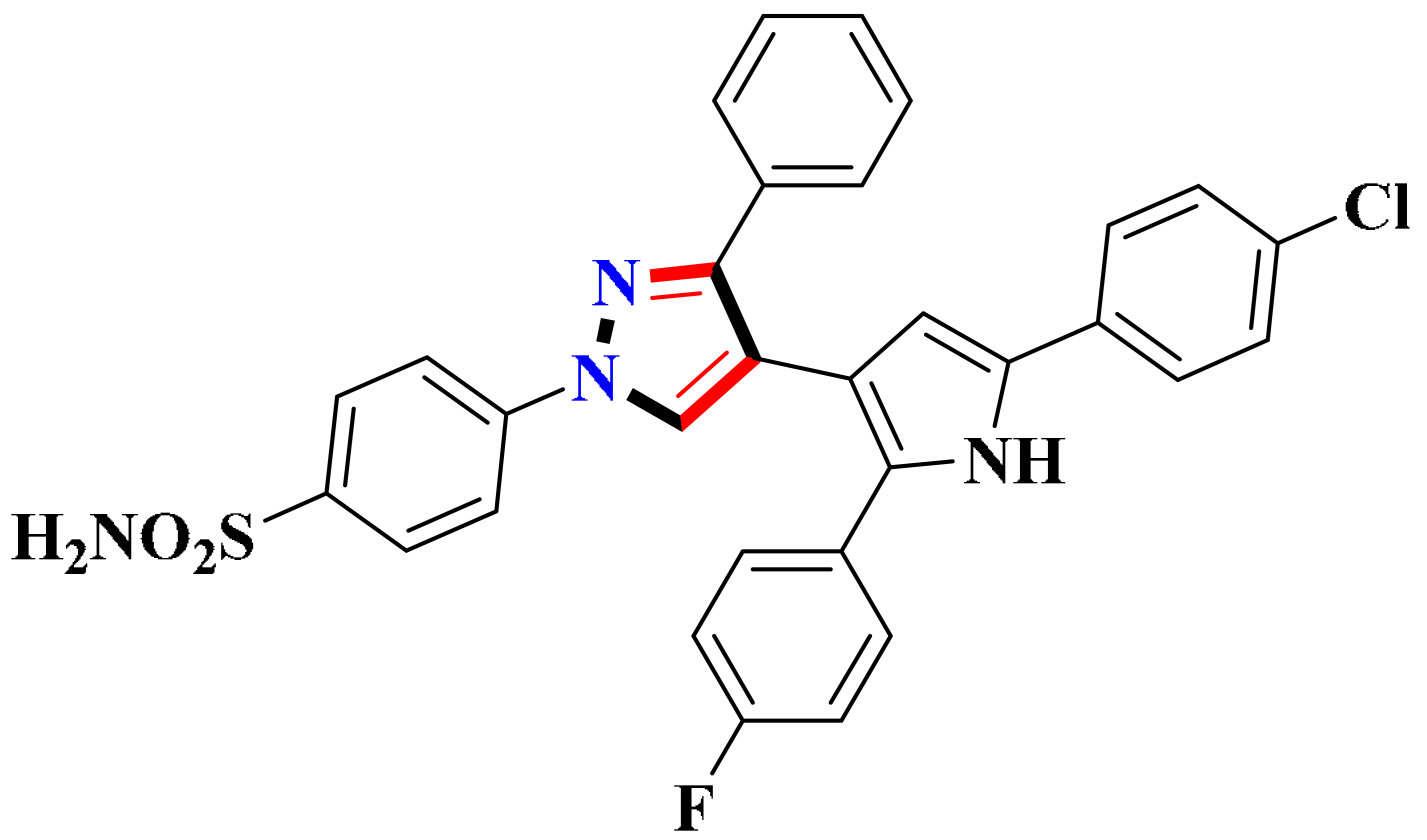	-	-
**152**	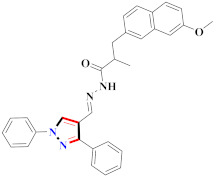		-
**153**	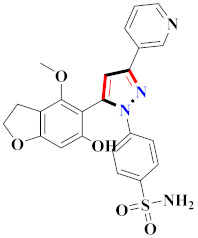	-	-
**154**	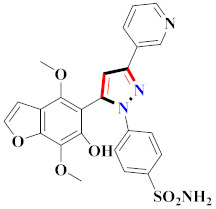	-	-
**155**	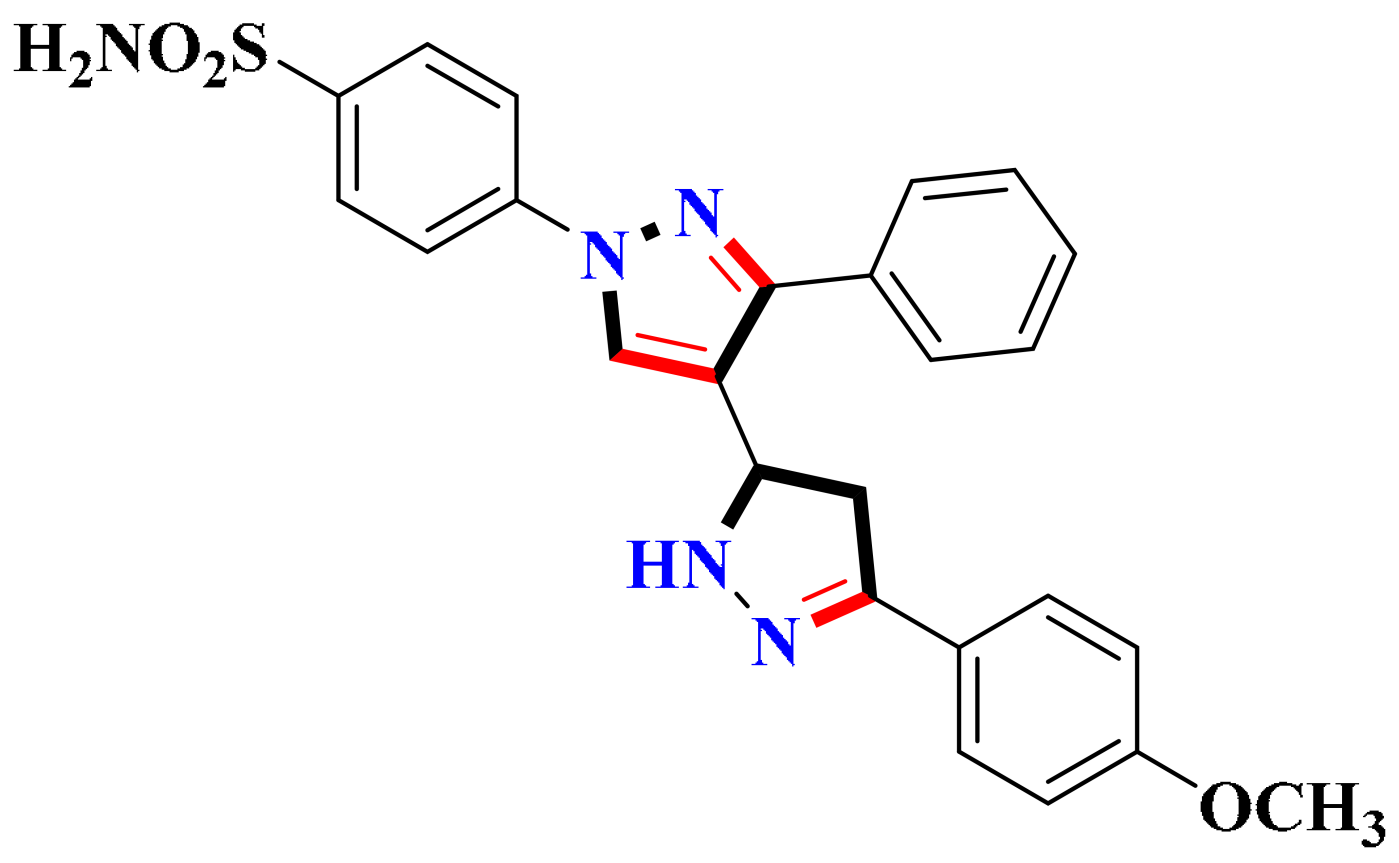	COX (I/II)	SI = 11.1
**156**	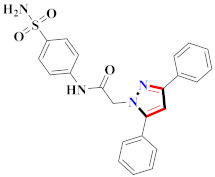	COX-2	IC_50_ = 0.44 µM
**157**	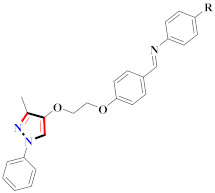	COX-2	IC_50_ = 0.51 µM
**158**	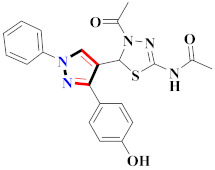		64.6% inhibition
**159**	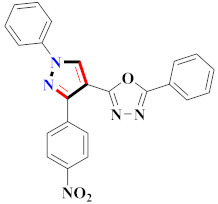	-	-
**160**	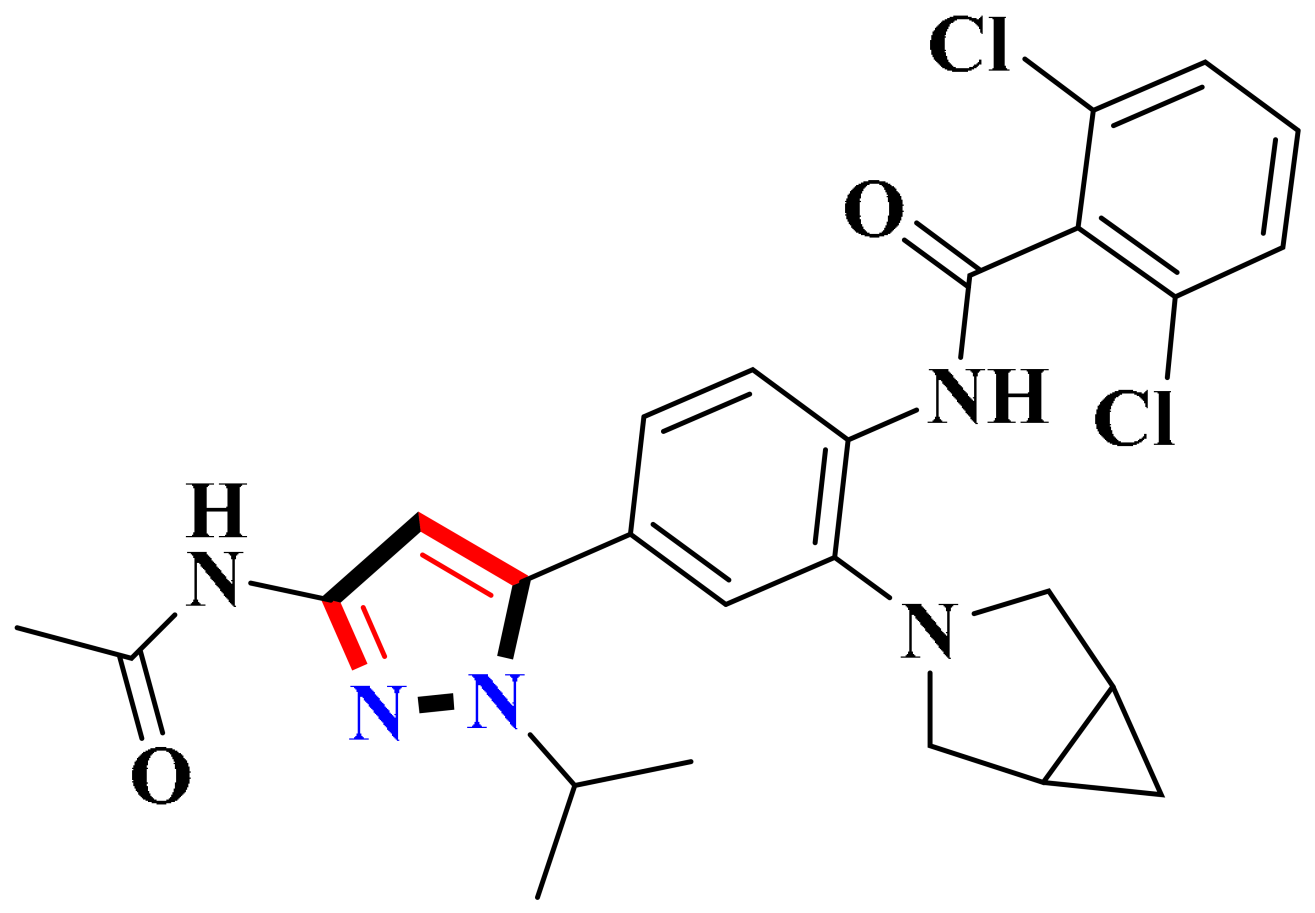	RORy Fret	IC_50_ = 3.0 nM
**161**	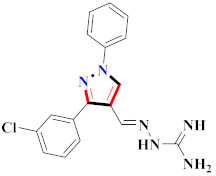	Inhibition	93.59%

**Table 12:** Representative compound structures **162** to **175** and their anti-inflammatory activity.

Compounds	Structure	Anti-Inflammatory Activity	
		Target Site	Biological Activity Value
**162**	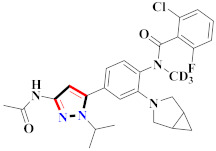	RORy Fret	IC_50_ = 3.0 nM
**163**	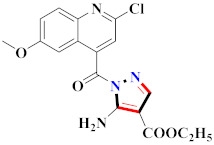	COX2	IC_50_ = 0.26 µM
		COX1	IC_50_ = >50 µM
**164**	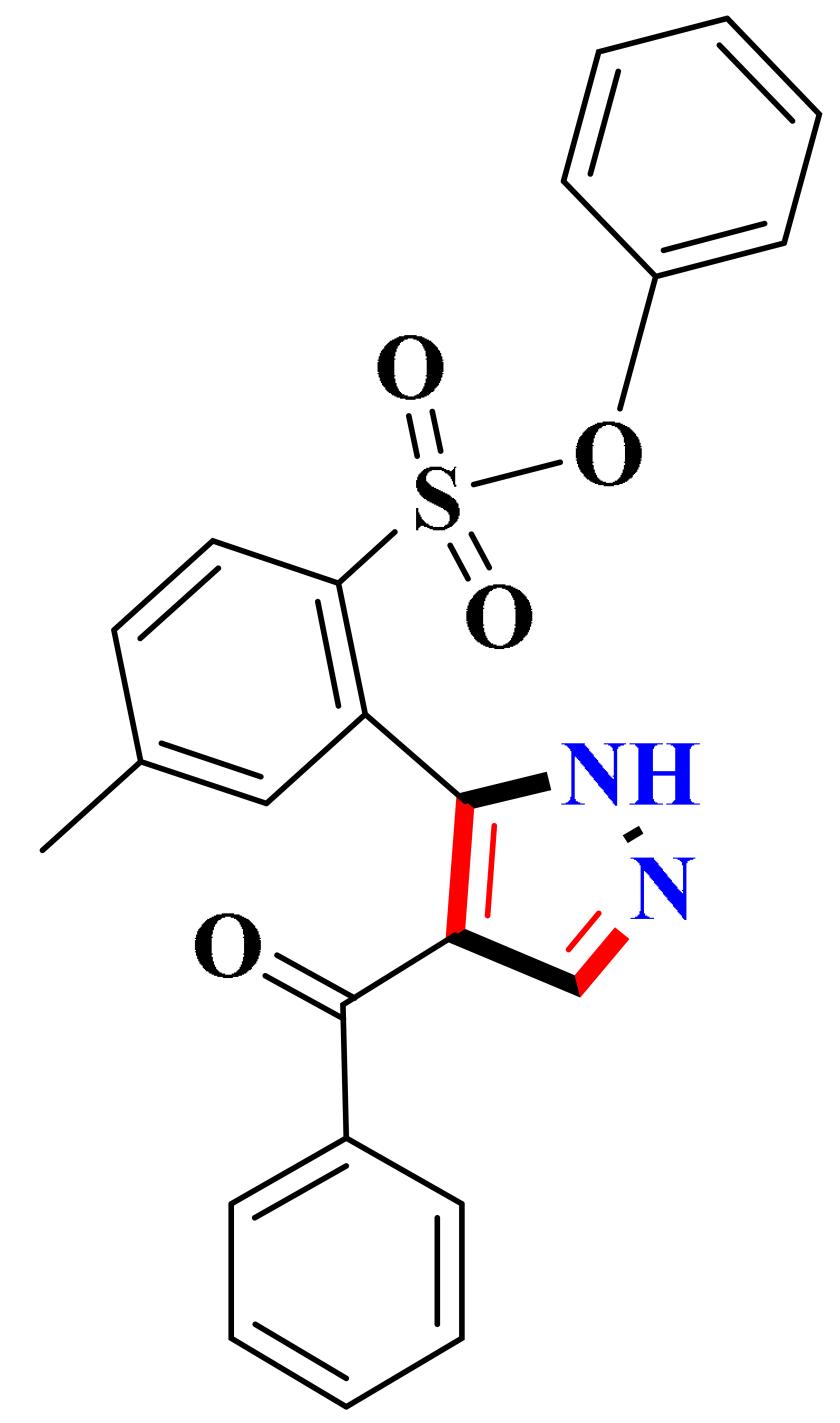	Anti-inflammatory activity	4.74%
**165**	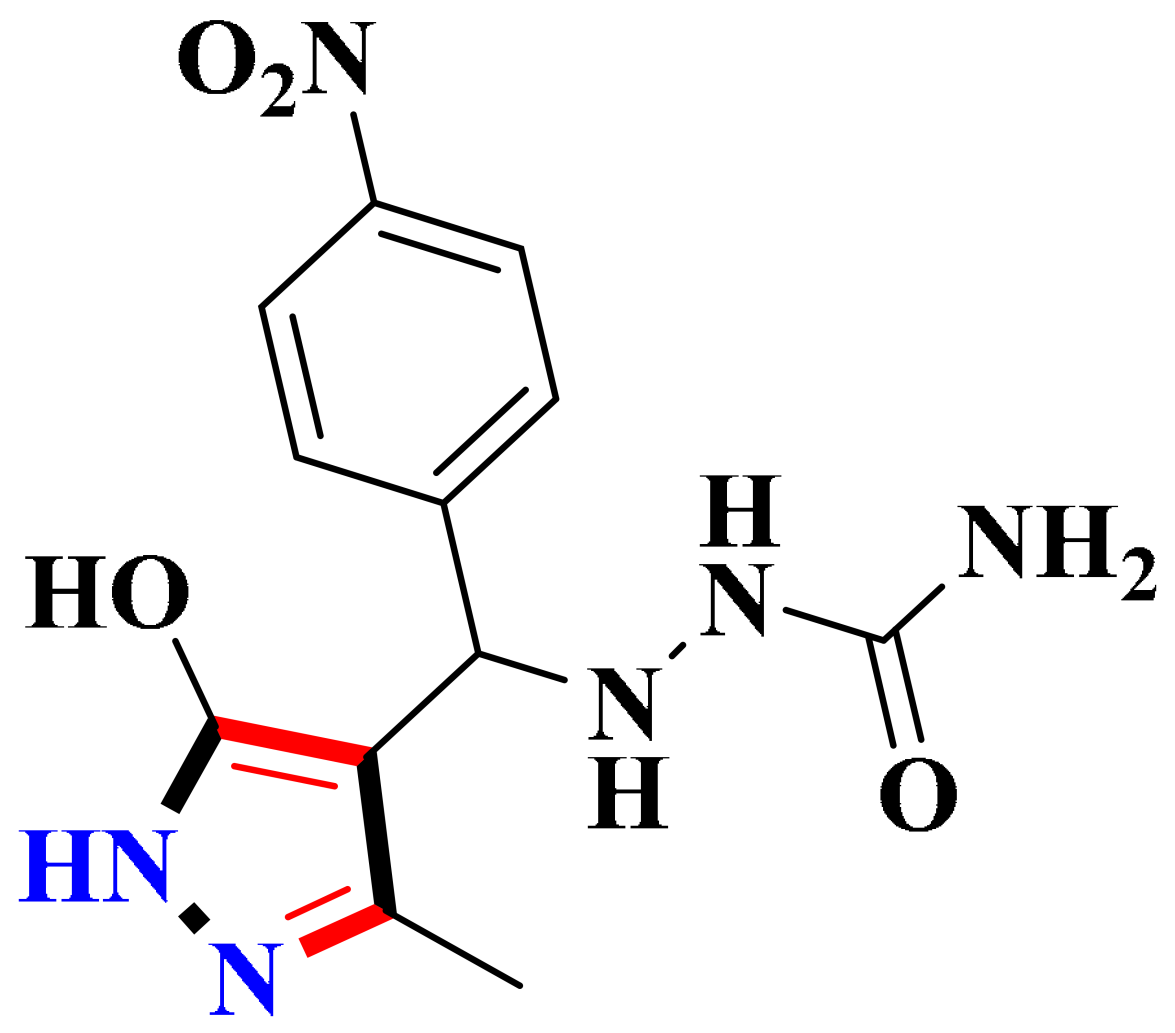	Percentage (%) of Activity, Dose (10 mg/kg)	82.1%
**166**	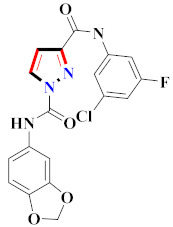	Lox-1	IC_50_ = 17 nM
**167**	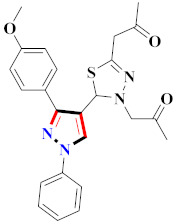	COX-2	IC_50_ = 1.33 µM
**168**	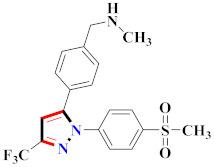		89% inhibition
**169**	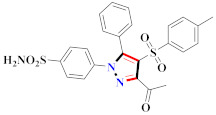		51 ± 0.7 mg/kg 51 ± 0.7 mg/kg
**170**	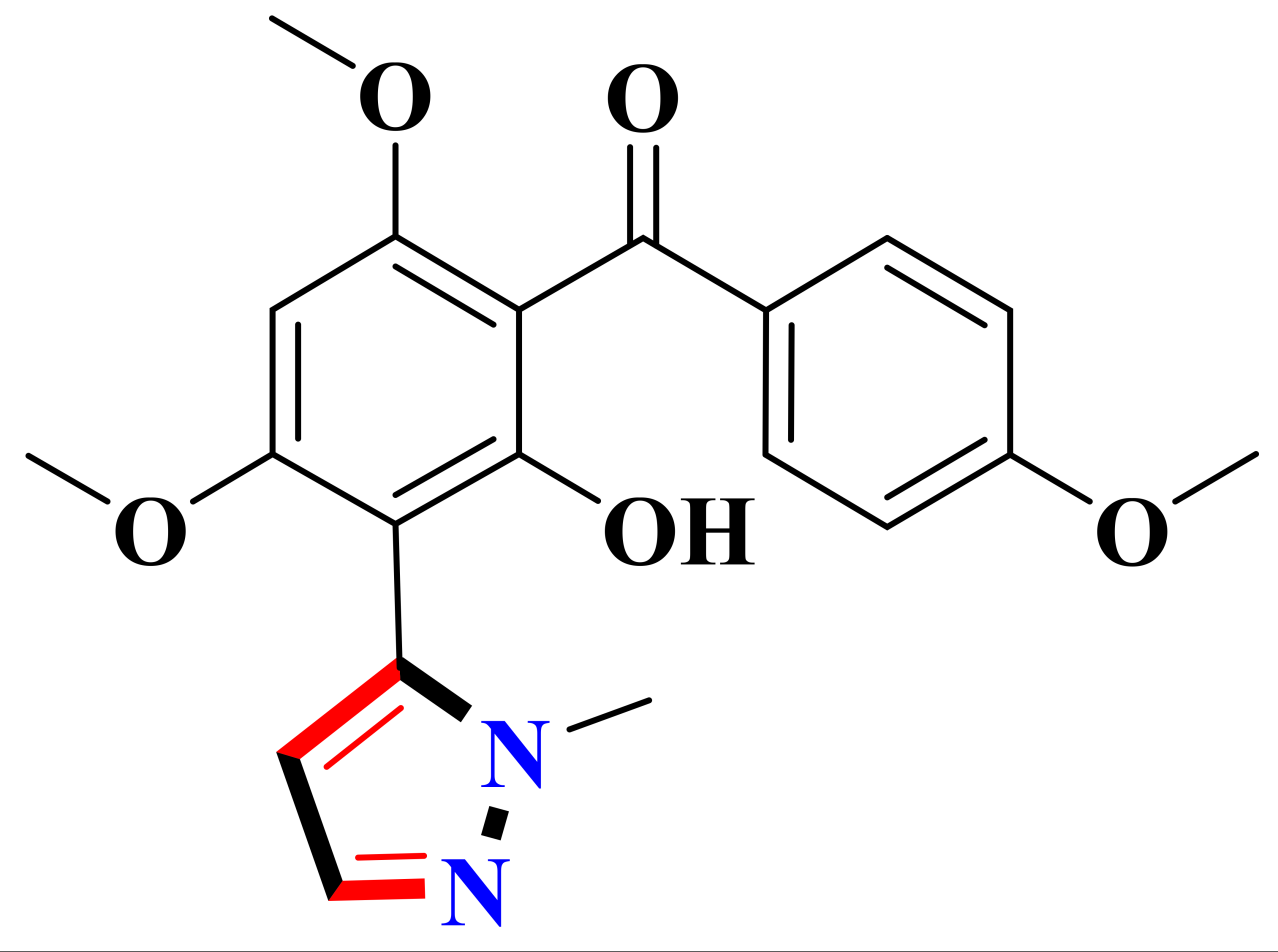	COX1	IC_50_ = 61.35 µM
		COX2	IC_50_ = 60.62 µM
**171**	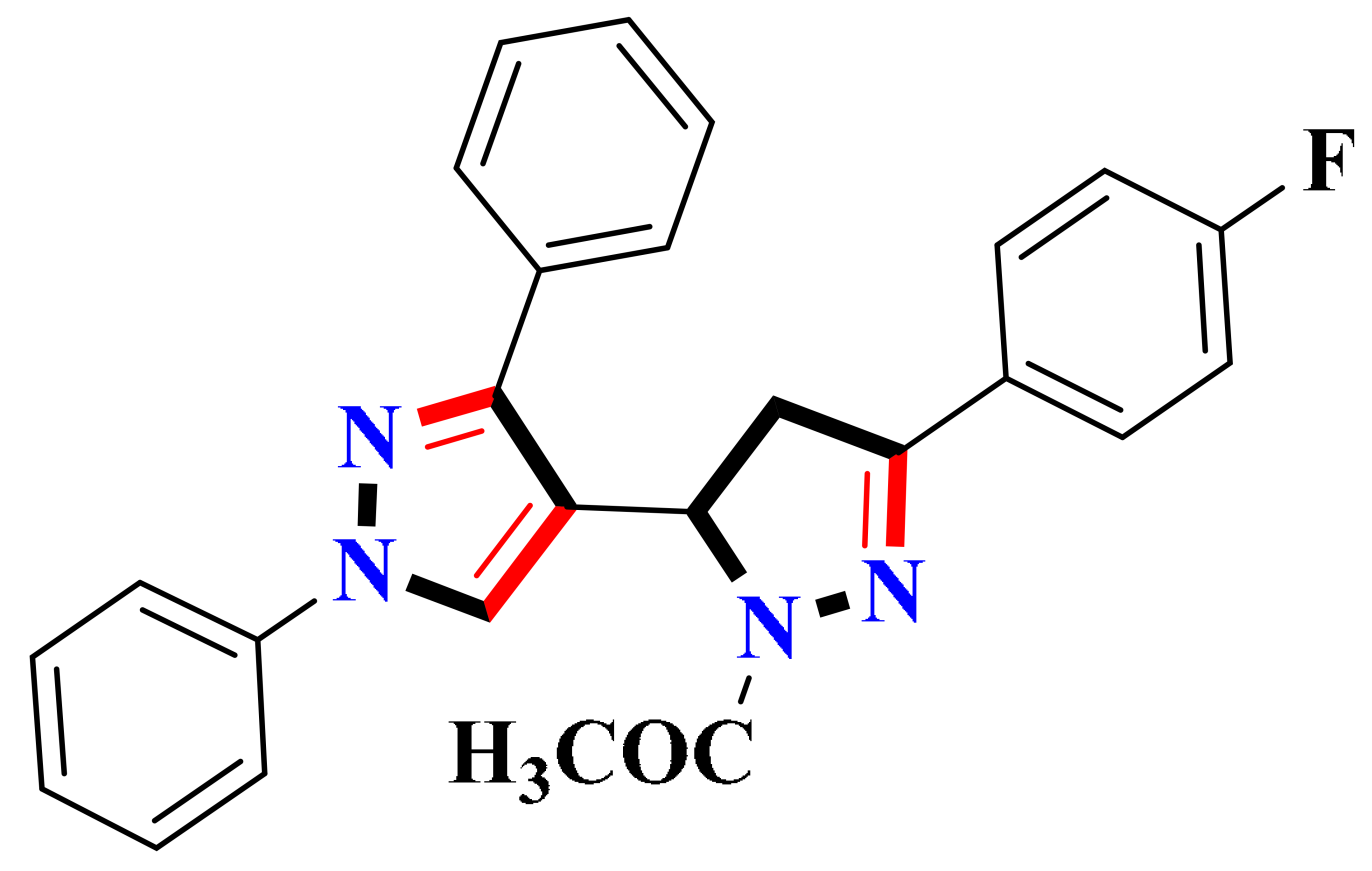	Anti-inflammatory activity Mean paw volume (mL) ± SEM	2.25 ± 0.45%
**172**	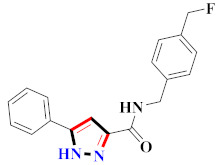	Anti-inflammatory activity Mean paw volume (mL) ± SEM	1.25 ± 0.03%
**173**	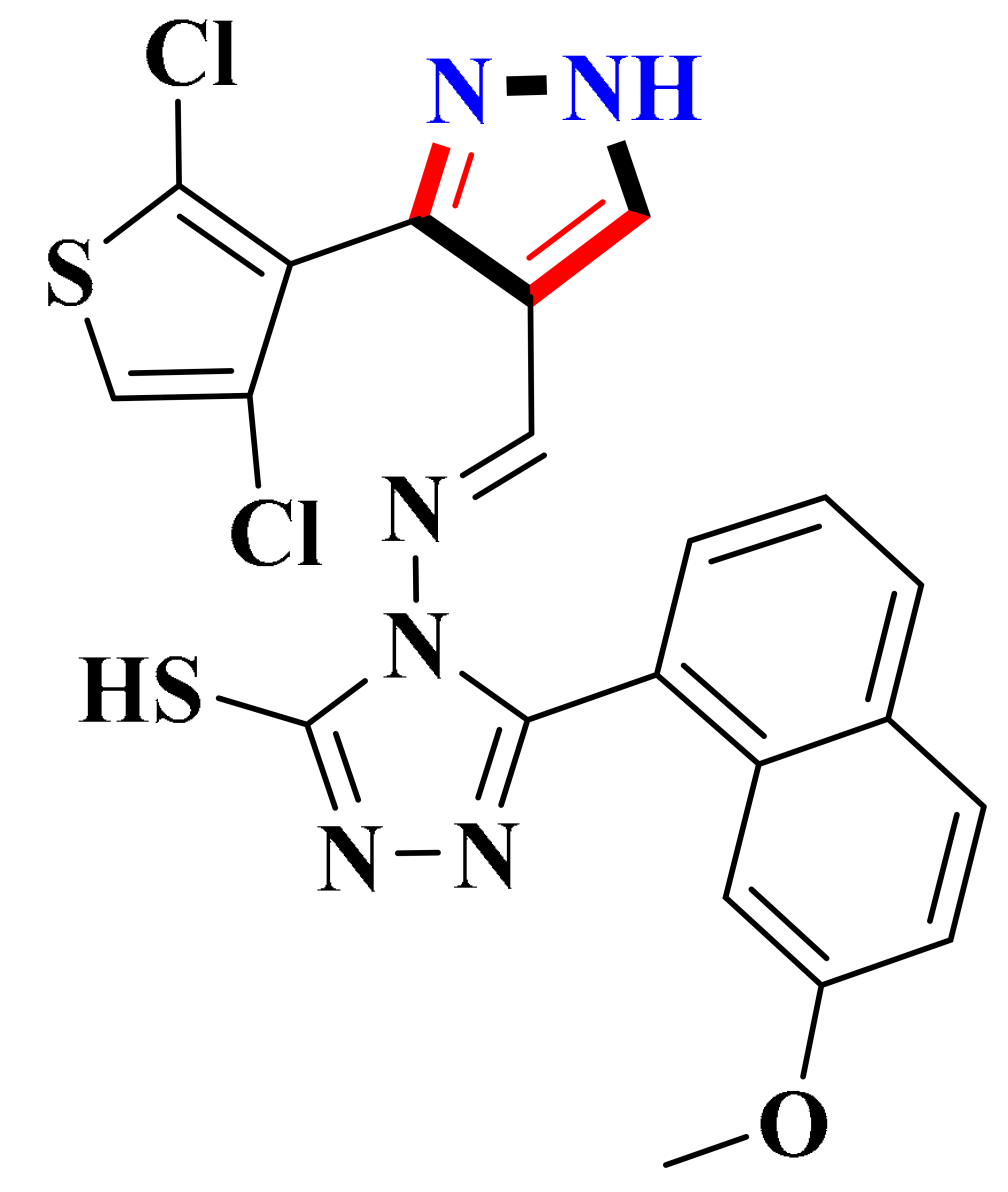	-	-
**174**	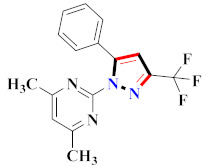	Anti-inflammatory	Volume of edema = 0.66 mL
**175**	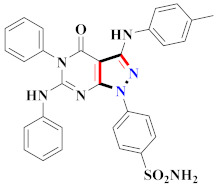	% Inhibition	68.85%

**Table 13:** Representative compound structures **176** to **187** and their anti-inflammatory activity.

Compounds	Structure	Anti-Inflammatory Activity	
		Target Site	Biological Activity Value
**176**	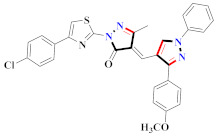	COX-1	IC_50_ = 20.78 µM
**177**	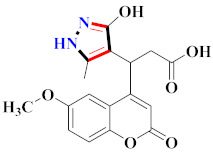	% inhibition	63.11%
**178**	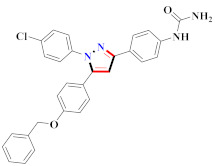	p38α MAPK inhibition	IC_50_ = 0.069 mM
**179**	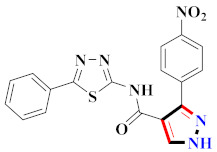	% inhibition	81.00%
**180**	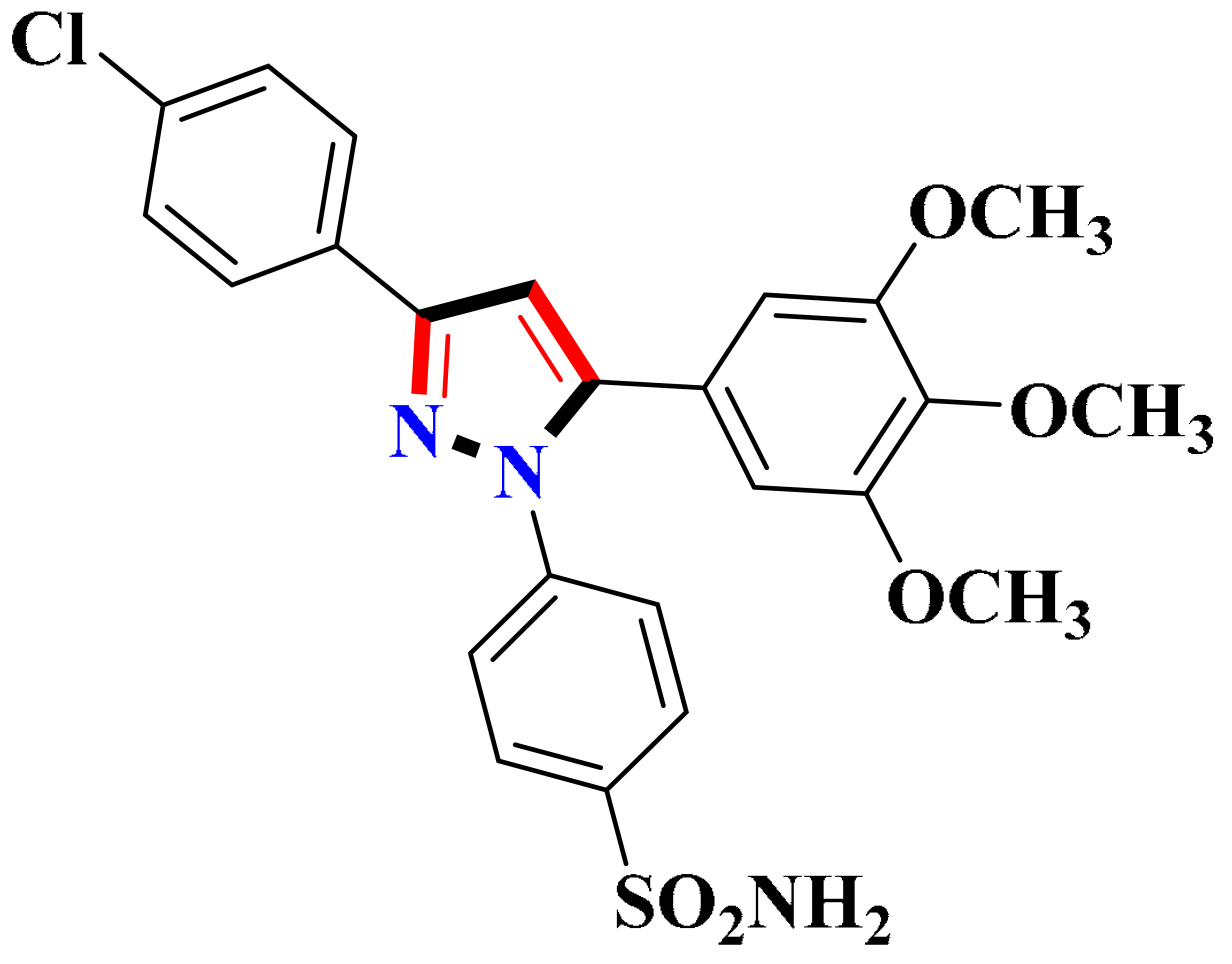	Anti-inflammatory	ED_50_ = 53.99 µmol/kg
**181**	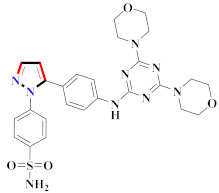	COX-2	IC_50_ = 0.74 µM
**182**	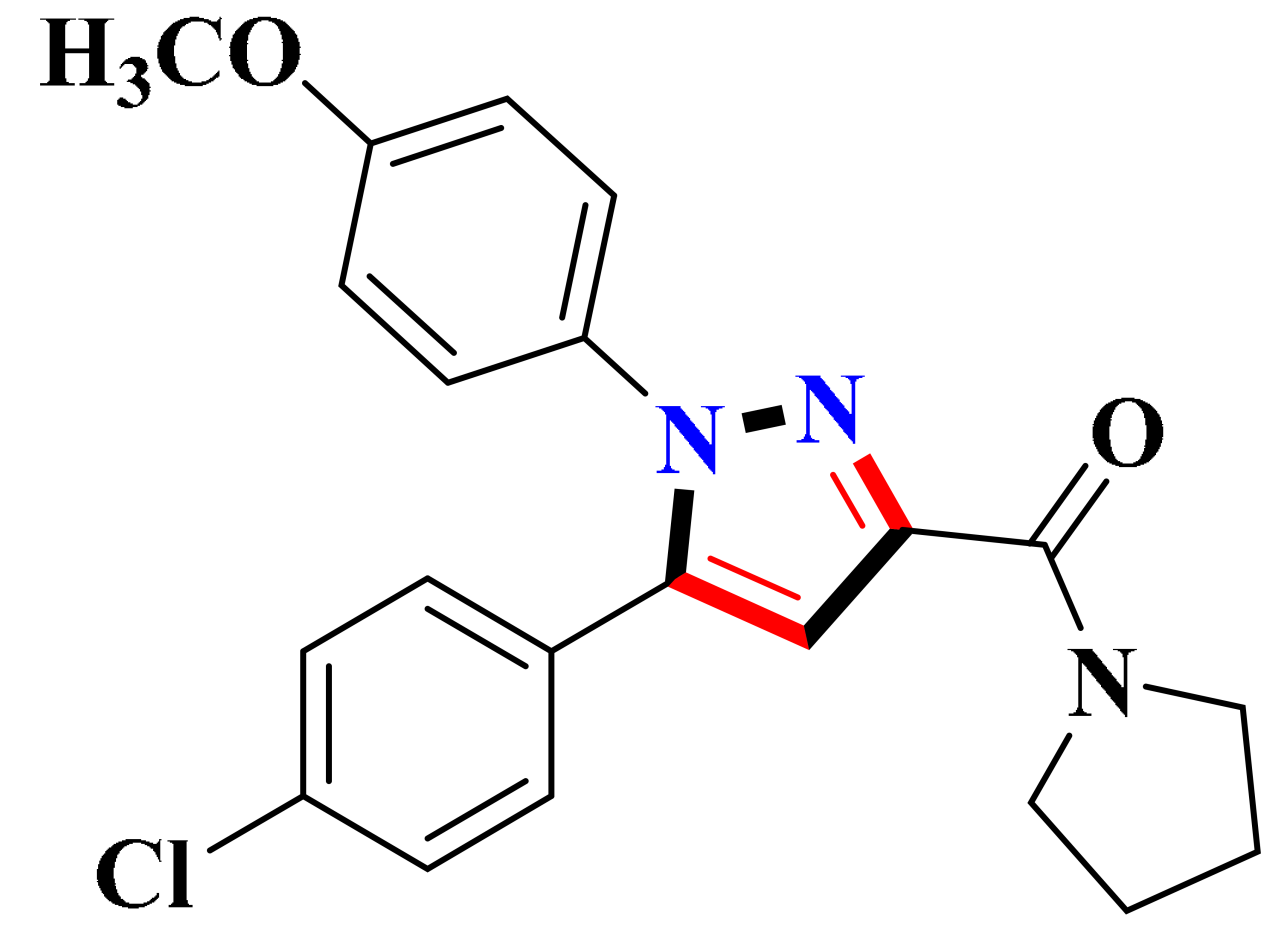	-	IC_50_ = 5.7–8.3 nM
**183**	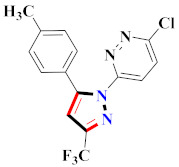	COX1/2	IC_50_ = 8 µM
**184**	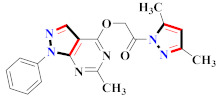	COX-2	IC_50_ = 1.11 µM
**185**	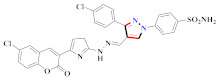	Anti-inflammatory	90.39% inhibition
**186**	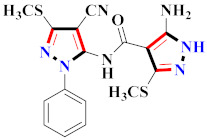	COX-2	IC_50_ = 11.2 µM
**187**	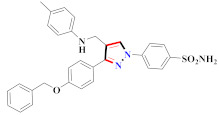	COX-2	IC_50_ = 1.79 µM

**Table 14:** Representative compound structures **188** to **191** and their anti-inflammatory activity.

Compounds	Structure	Anti-Inflammatory Activity	
		Target Site	Biological Activity Value
**188** (AD732)	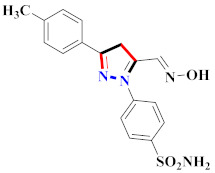	COX-2	IC_50_ = 0.57 µM
**189a**	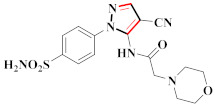	COX-2	IC_50_ = 39.43 nM
**189b**	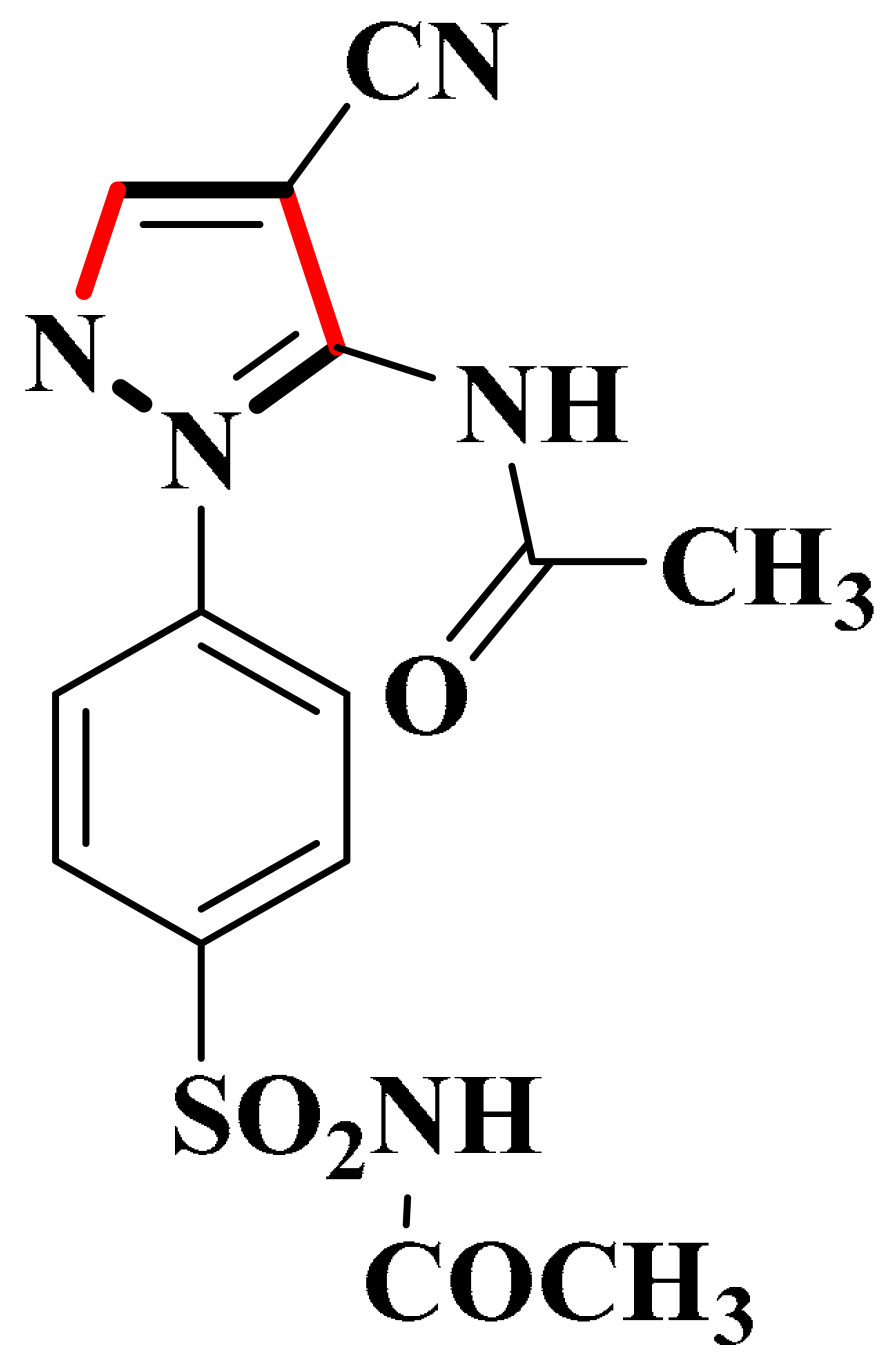	COX-2	IC_50_ = 61.2 nM
**189c**	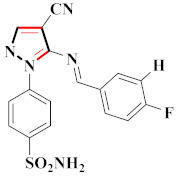	COX-2	IC_50_ = 38.73 nM
**189d**	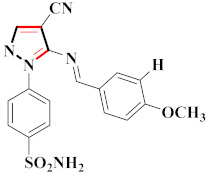	COX-2	IC_50_ = 39.14 nM
**190a**	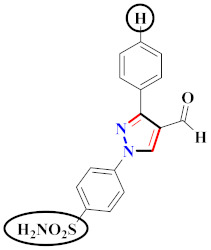	COX-2	IC_50_ = 0.017 µM
		COX-1	IC_50_ = 0.263 µM
**190b**	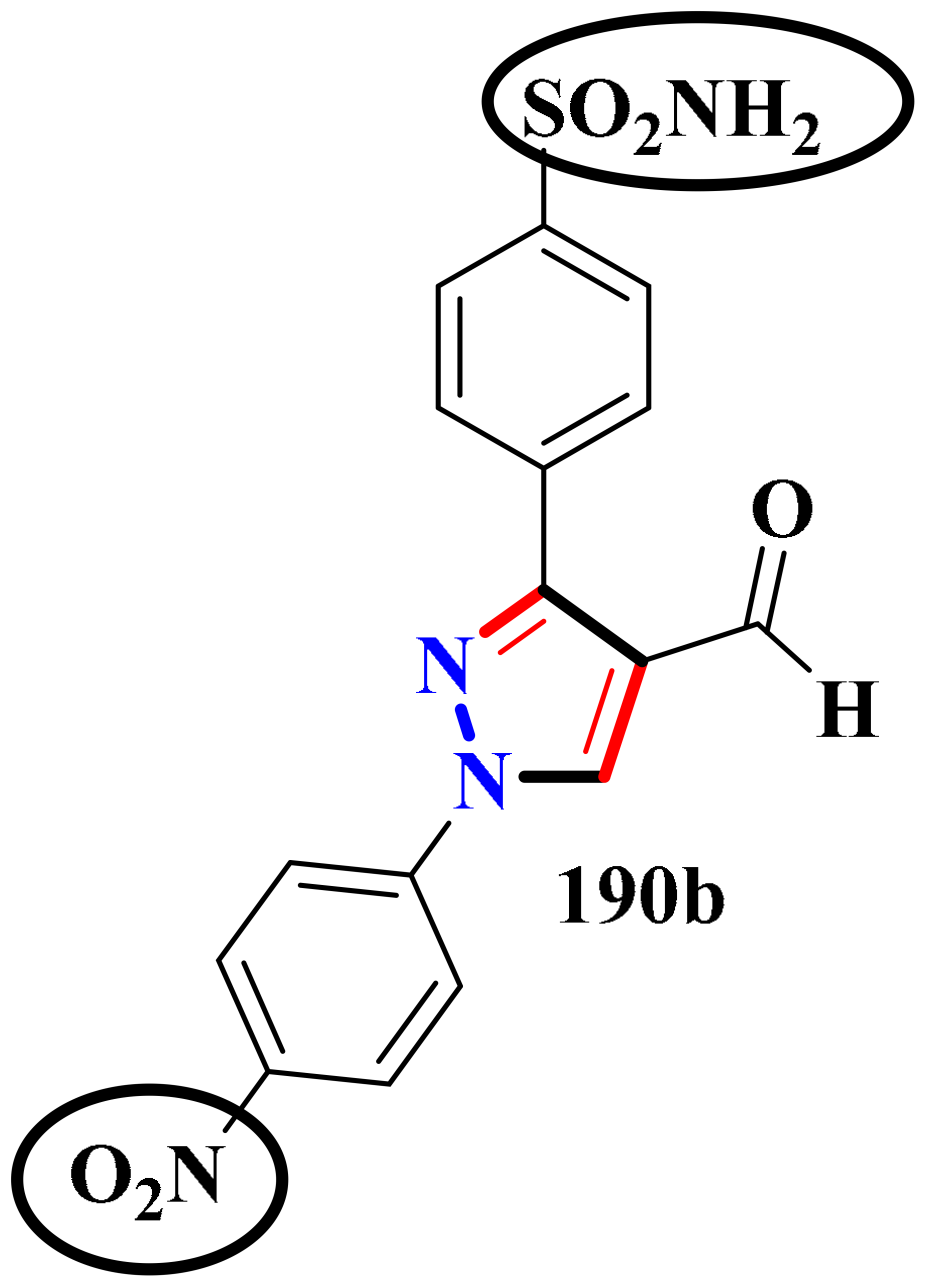	COX-1	IC_50_ = 0.012 µM
**191**	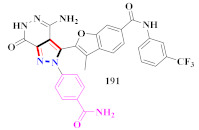	DDR1	IC_50_ = 10.6 nM

## Data Availability

Not applicable.
